# Supplementary material for: Predictive machine learning models for ascending aortic dilatation in patients with bicuspid and tricuspid aortic valves undergoing cardiothoracic surgery: a prospective, single-centre and observational study
Source: BMJ Open. 2024 Mar 20;14(3):e067977. doi: 10.1136/bmjopen-2022-067977 (PMC10961501; doi:10.1136/bmjopen-2022-067977)
Supplement: Supplementary data [file bmjopen-2022-067977supp001.pdf]

***Predictive Machine Learning Models for Ascending Aortic Dilatation in Patients with Bicuspid and Tricuspid Aortic Valves Undergoing Cardiothoracic Surgery: A prospective, single-center and observational study***

**Supplementary Appendix Table of Contents:**

**Tables**

**e-Table 1:** Characteristics of Patients with BAV versus TAV.
**e-Table 2.** Additional cardiovascular drugs including anti-hypertensive therapy.
**e-Table 3.** Performance of the algorithms.
**e-Table 4:** Predictors of ascending aortic dilatation in BAV and TAV patients, separately. Univariate and Multivariate logistic regression analysis: Imputed data.
**e-Table 5:** Baseline characteristics of included and excluded study participants.
**e-Table 6:** Contributors to the prediction of aortic dilatation in TAV.

**Figures**

**e-Figure 1:** Flowchart of the study
**e-Figure 2:** Plot of methodology used to choose variables.
**e-Figure 3:** The ROC curve for our risk prediction model across the predictive methods using imputed data.
**e-Figure 4.** Principal component analysis (PCA) plot of TAV patients with or without aneurysm stratified by AS
**e-Figure 5.** The ROC curve for our risk prediction model across the predictive methods without AS as predictor;

**Dataset selection:**

In order to gather maximum of information from the available measurement in the ASAP/DAVAACA cohort, we first created several blocks of meaningful variables: clinical data, family history of cardiovascular diseases, prevalent diseases, demographic characteristics, lifestyle habits data, blood sample data and medication. Then to select the most meaningful variables by avoiding collinearity within each bloc, we performed several analyses: (1) a hierarchical clustering, then (2) we ran Principal Component Analysis, (3) we lastly created the correlation matrix inside each cluster (and its correlation plot) (**Figure e-2**).

**e-Table 1:** Characteristics of Patients with BAV versus TAV

| | BAV N=543 | TAV N=491 | P value |
|---|---|---|---|
| **Gender: Male** | 397 (73.1%) | 311 (63.3%) | 0.001 |
| **Age** | 60.4 (12.4) | 70.4 (9.09) | <0.001 |
| **Height** | 175 (9.30) | 172 (9.80) | <0.001 |
| **Weight** | 81.9 (15.8) | 81.5 (15.9) | 0.697 |
| **BSA** | 1.98 (0.22) | 1.94 (0.22) | 0.012 |
| **BMI** | 26.6 (4.26) | 27.4 (4.77) | 0.002 |
| **Regular Smoker:** | | | 0.023 |
| **No** | 250 (46.6%) | 213 (43.6%) | |
| **Former** | 234 (43.7%) | 246 (50.4%) | |
| **Yes** | 52 (9.70%) | 29 (5.94%) | |
| **Systolic Blood Pressure** | 136 (18.6) | 142 (20.4) | <0.001 |
| **Diastolic Blood Pressure** | 80.1 (11.9) | 76.1 (13.2) | <0.001 |
| **PP** | 55.4 (17.6) | 66.4 (19.7) | <0.001 |
| **Leukocytes** | 5.80 [4.80;6.90] | 6.00 [5.03;7.40] | 0.004 |
| **hsCRP** | 1.10 [0.55;2.50] | 1.80 [0.79;3.80] | <0.001 |
| **Cholesterol** | 4.80 [4.20;5.60] | 4.60 [3.90;5.60] | 0.016 |
| **LDL** | 2.80 [2.20;3.60] | 2.60 [2.00;3.50] | 0.009 |
| **Aortic Stenosis:** | 404 (74.4%) | 306 (62.3%) | <0.001 |
| **Aortic Insufficiency:** | 158 (29.1%) | 160 (32.6%) | 0.252 |
| **Diameter of Aortic Anulus** | 24.8 (3.57) | 23.0 (3.08) | <0.001 |
| **Diameter of Valsalva Sinus** | 35.6 (5.68) | 34.9 (6.94) | 0.056 |
| **Diameter of Sinotubular Junction** | 30.5 (5.70) | 29.0 (6.49) | <0.001 |
| **Diameter of Ascending Aorta** | 39.3 (8.29) | 37.1 (10.1) | <0.001 |
| **Dilatation of Ascending Aorta:** | 297 (54.7%) | 349 (71.1%) | <0.001 |
| **Sibling MI history before 65 year** | 32 (6.24%) | 46 (10.0%) | 0.041 |
| **Mother MI history before 65 year** | 25 (4.84%) | 32 (6.97%) | 0.199 |
| **Father MI history before 65 year** | 69 (13.7%) | 63 (14.3%) | 0.874 |
| **Myocardial Infarction** | 19 (3.53%) | 42 (8.59%) | 0.001 |
| **Stroke** | 30 (5.55%) | 57 (11.7%) | 0.001 |
| **Abdominal Aorta Aneurysm** | 25 (4.63%) | 30 (6.19%) | 0.335 |
| **Angina Pectoris** | 35 (6.49%) | 61 (12.5%) | 0.001 |
| **Heart Failure** | 40 (7.50%) | 57 (11.7%) | 0.029 |
| **Hypertension** | 255 (47.4%) | 311 (64.4%) | <0.001 |
| **Diabetes** | 54 (10.0%) | 64 (13.1%) | 0.139 |
| **Anti-hypertensive drugs** | | | |
| **ACE inhibitor** | 103 (19.0%) | 142 (29.0%) | <0.001 |
| **Aspirin** | 132 (24.4%) | 193 (39.6%) | <0.001 |
| **Betablocker** | 217 (40.1%) | 255 (52.0%) | <0.001 |

**e-Table 2.** Additional cardiovascular drugs including anti-hypertensive therapy.

| | BAV patients (n=543) | | | TAV patients (n=491) | | |
|---|---|---|---|---|---|---|
| | **Dilated (n=246)** | **Non-Dilated (n=297)** | **P value** | **Dilated (n=142)** | **Non-Dilated (n=349)** | **P value** |
| **ACE inhibitor** | 46 (18.7%) | 57 (19.3%) | 0.941 | 45 (31.9%) | 97 (27.9%) | 0.434 |
| **Angiotensin II blocker** | 42 (17.1%) | 45 (15.3%) | 0.648 | 38 (27.0%) | 78 (22.5%) | 0.350 |
| **Beta blocker** | 92 (37.4%) | 125 (42.4%) | 0.277 | 76 (53.9%) | 179 (51.3%) | 0.672 |
| **Calcium antagonist** | 40 (16.3%) | 41 (13.9%) | 0.518 | 40 (28.4%) | 87 (24.9%) | 0.501 |
| **Loop diuretics** | 22 (8.94%) | 35 (11.9%) | 0.330 | 19 (13.5%) | 65 (18.6%) | 0.216 |
| **Thiazide diuretics*** | 1 (0.81%) | 1 (0.72%) | 0.931 | 2 (2.99%) | 5 (3.70%) | 0.793 |
| **Lipid lowering agent** | 79 (32.1%) | 107 (36.3%) | 0.356 | 45 (32.1%) | 193 (55.3%) | <0.001 |
| **Anticoagulant** | 28 (11.4%) | 26 (8.81%) | 0.396 | 12 (8.51%) | 50 (14.4%) | 0.107 |
| **Insulin** | 4 (1.63%) | 13 (4.41%) | 0.112 | 0 (0.00%) | 17 (4.87%) | 0.005 |
| **Peroral antidiabetics*** | 4 (3.25%) | 5 (3.60%) | 0.878 | 2 (2.99%) | 16 (11.9%) | 0.069 |

*Results concerning these variables should be precocious due to high rate of missing value (n=464).

**e-Table 3.** Performance of the algorithms

| Method | Accuracy | AUC | Sensitivity | Specificity | Positive Predictive Value | Negative Predictive Value |
|---|---|---|---|---|---|---|
| BAV | | | | | | |
| **Method** | **Accuracy** | **AUC** | **Sensitivity** | **Specificity** | **Positive Predictive Value** | **Negative Predictive Value** |
| **GLM** | 0.528 | 0.529 | 0.444 | 0.609 | 0.526 | 0.528 |
| **StepAIC** | 0.506 | 0.535 | 0.422 | 0.587 | 0.500 | 0.509 |
| **Lasso** | 0.484 | 0.507 | 0.422 | 0.544 | 0.475 | 0.490 |
| **Random Forest** | 0.517 | 0.540 | 0.467 | 0.565 | 0.512 | 0.520 |
| **Neural Network** | 0.473 | 0.518 | 0.489 | 0.457 | 0.468 | 0.477 |
| TAV | | | | | | |
| **Method** | **Accuracy** | **AUC** | **Sensitivity** | **Specificity** | **Positive Predictive Value** | **Negative Predictive Value** |
| **GLM** | 0.824 | 0.811 | 0.820 | 0.833 | 0.911 | 0.690 |
| **StepAIC** | 0.824 | 0.815 | 0.900 | 0.667 | 0.849 | 0.762 |
| **Lasso** | 0.824 | 0.854 | 0.860 | 0.750 | 0.878 | 0.720 |
| **Random Forest** | 0.797 | 0.882 | 0.840 | 0.708 | 0.857 | 0.680 |
| **Neural Network** | 0.770 | 0.825 | 0.820 | 0.667 | 0.837 | 0.640 |
| BAV (Imputed data) | | | | | | |
| **Method** | **Accuracy** | **AUC** | **Sensitivity** | **Specificity** | **Positive Predictive Value** | **Negative Predictive Value** |
| **GLM** | 0.570 | 0.586 | 0.689 | 0.426 | 0.593 | 0.531 |
| **StepAIC** | 0.563 | 0.526 | 0.676 | 0.426 | 0.588 | 0.520 |
| **Lasso** | 0.578 | 0.593 | 0.676 | 0.459 | 0.602 | 0.539 |
| **Random Forest** | 0.556 | 0.556 | 0.662 | 0.426 | 0.583 | 0.548 |
| **Neural Network** | 0.541 | 0.540 | 0.581 | 0.492 | 0.581 | 0.492 |
| TAV (Imputed data) | | | | | | |
| **Method** | **Accuracy** | **AUC** | **Sensitivity** | **Specificity** | **Positive Predictive Value** | **Negative Predictive Value** |
| **GLM** | 0.820 | 0.831 | 0.816 | 0.829 | 0.922 | 0.644 |
| **StepAIC** | 0.803 | 0.838 | 0.816 | 0.771 | 0.899 | 0.628 |
| **Lasso** | 0.812 | 0.842 | 0.793 | 0.857 | 0.932 | 0.625 |
| **Random Forest** | 0.820 | 0.857 | 0.828 | 0.800 | 0.911 | 0.651 |
| **Neural Network** | 0.812 | 0.799 | 0.862 | 0.686 | 0.872 | 0.667 |

**e-Table 4.** Predictors of aortopathy in BAV and TAV patients separately, Univariate and Multivariate logistic regression analysis: Imputed data

| | **BAV imputed (N= 543)** | | | | | | **TAV imputed (n= 491)** | | | | | |
|---|---|---|---|---|---|---|---|---|---|---|---|---|
| | ***unAdjusted Model*** | | ***Model 1*** | | ***Model 2*** | | ***unAdjusted Model*** | | ***Model 1*** | | ***Model 2*** | |
| | ***OR (95% IC)**** | ***p*** | ***OR (95% IC)**** | ***p*** | ***OR (95% IC)**** | ***p*** | ***OR (95% IC)**** | ***p*** | ***OR (95% IC)**** | ***p*** | ***OR (95% IC)**** | ***p*** |
| **Aortic Stenosis** | 0.58 (0.39-0.85) | 0.006 | 0.43 (0.27-0.67) | 0.002 | 0.42 (0.22-0.79) | 0.007 | 0.05 (0.03-0.07) | <0.001 | 0.04 (0.02-0.07) | <0.001 | 0.02 (0.01-0.05) | <0.001 |
| **Aortic Regurgitation** | 1.51 (1.04-2.19) | 0.031 | 1.89 (1.24-2.88) | 0.003 | 1.35 (0.75-2.44) | 0.316 | 7.99 (5.19-12.45) | <0.001 | 7.27 (4.56-11.77) | <0.001 | 0.51 (0.20-1.18) | 0.133 |
| **Pulse Pressure** | 0.99 (0.98-1.00) | 0.009 | 0.99 (0.98-1.00) | 0.013 | 0.98 (0.97-0.99) | <0.001 | 1.01 (1.00-1.02) | 0.088 | 1.01 (1.00-1.02) | 0.163 | 0.99 (0.98-1.00) | 0.132 |
| **Diabetes** | 0.42 (0.22-0.76) | 0.006 | 0.34 (0.18-0.65) | 0.001 | 0.39 (0.19-0.74) | 0.005 | 0.10 (0.02-028) | <0.001 | 0.10 (0.02-0.29) | <0.001 | 0.21 (0.05-0.71) | 0.022 |

* Odds ratio and 95% confidence interval limits were obtained by logistic regression and were adjusted for age, sex, body surface area (BSA), low-density Lipoprotein (LDL), high-sensitivity C-reactive protein (hsCRP) and Diabetes

**e-Table 5.** Baseline characteristics of included and excluded study participants

| | [ALL] N=1180 | Data N=1041 | NA N=139 | p.overall | N |
|---|---|---|---|---|---|
| **Gender: Male** | 807 (68.4%) | 708 (68.5%) | 99 (67.8%) | 0.947 | 1180 |
| **Age** | 64.9 (12.4) | 65.2 (12.0) | 62.9 (14.5) | 0.077 | 1180 |
| **Height** | 174 (9.60) | 174 (9.65) | 175 (9.15) | 0.263 | 1171 |
| **Weight** | 81.8 (15.6) | 81.7 (15.8) | 82.6 (14.4) | 0.514 | 1168 |
| **BSA** | 1.96 (0.21) | 1.96 (0.22) | 1.98 (0.19) | 0.305 | 1141 |
| **BMI** | 27.0 (4.56) | 27.0 (4.53) | 27.1 (4.80) | 0.746 | 1167 |
| **Regular Smoker:** | | | | 0.549 | 1168 |
| **No** | 535 (45.8%) | 463 (45.2%) | 72 (50.0%) | | |
| **Former** | 541 (46.3%) | 480 (46.9%) | 61 (42.4%) | | |
| **Yes** | 92 (7.88%) | 81 (7.91%) | 11 (7.64%) | | |
| **Cusps:** | | | | 0.951 | 1140 |
| **BAV** | 599 (52.5%) | 543 (52.5%) | 56 (52.8%) | | |
| **TAV** | 541 (47.5%) | 491 (47.5%) | 50 (47.2%) | | |
| **Raphe:** | | | | . | 1158 |
| **True BAV** | 50 (4.32%) | 47 (4.60%) | 3 (2.19%) | | |
| **Left-Non coronary** | 6 (0.52%) | 5 (0.49%) | 1 (0.73%) | | |
| **Right-Non coronary** | 99 (8.55%) | 90 (8.81%) | 9 (6.57%) | | |
| **Right-Left** | 432 (37.3%) | 388 (38.0%) | 44 (32.1%) | | |
| **Systolic Blood Pressure** | 138 (19.7) | 139 (19.8) | 136 (18.8) | 0.053 | 1172 |
| **Diastolic Blood Pressure** | 78.3 (12.7) | 78.2 (12.7) | 79.3 (12.7) | 0.305 | 1171 |
| **PP** | 60.1 (19.2) | 60.6 (19.4) | 56.2 (17.2) | 0.005 | 1171 |
| **Leukocytes** | 5.90 [4.90;7.10] | 5.90 [4.90;7.10] | 5.90 [4.90;6.90] | 0.665 | 1168 |
| **hsCRP** | 1.40 [0.64;3.20] | 1.40 [0.63;3.10] | 1.60 [0.72;3.52] | 0.153 | 1159 |
| **Cholesterol** | 4.80 [4.00;5.68] | 4.80 [4.00;5.60] | 5.10 [4.30;6.10] | 0.004 | 1162 |
| **LDL** | 2.80 [2.20;3.60] | 2.80 [2.10;3.50] | 3.20 [2.30;3.90] | 0.002 | 1146 |
| **Aortic Stenosis:** | 813 (69.1%) | 710 (68.7%) | 103 (72.0%) | 0.472 | 1177 |
| **Aortic Insufficiency:** | 361 (30.7%) | 318 (30.8%) | 43 (30.1%) | 0.944 | 1177 |
| **Diameter of Aortic Anulus** | 23.9 (3.40) | 24.0 (3.46) | 23.5 (2.87) | 0.148 | 1154 |
| **Diameter of Valsalva Sinus** | 35.4 (6.27) | 35.3 (6.32) | 36.2 (5.83) | 0.095 | 1157 |
| **Diameter of Sinotubular Junction** | 29.9 (6.05) | 29.8 (6.13) | 30.7 (5.34) | 0.083 | 1112 |
| **Diameter of Ascending Aorta** | 38.3 (9.24) | 38.2 (9.28) | 38.8 (8.09) | 0.686 | 1069 |
| **Dilatation of Ascending Aorta:** | 402 (37.6%) | 388 (37.5%) | 14 (40.0%) | 0.904 | 1069 |
| **Sibling MI history before 65-y** | 86 (7.76%) | 78 (8.02%) | 8 (5.93%) | 0.497 | 1108 |
| **Mother MI history before 65-y** | 63 (5.65%) | 57 (5.84%) | 6 (4.29%) | 0.583 | 1116 |
| **Father MI history before 65-y** | 151 (14.0%) | 132 (14.0%) | 19 (14.2%) | 0.959 | 1076 |
| **Myocardial Infarction** | 66 (5.64%) | 61 (5.94%) | 5 (3.47%) | 0.313 | 1171 |
| **Stroke** | 99 (8.43%) | 87 (8.45%) | 12 (8.33%) | 0.964 | 1174 |
| **Abdominal Aorta Aneurysm** | 58 (4.96%) | 55 (5.37%) | 3 (2.07%) | 0.132 | 1170 |
| **Angina Pectoris** | 103 (8.80%) | 96 (9.35%) | 7 (4.86%) | 0.105 | 1171 |
| **Heart Failure** | 112 (9.64%) | 97 (9.52%) | 15 (10.5%) | 0.828 | 1162 |
| **Hypertension** | 635 (54.5%) | 566 (55.4%) | 69 (47.6%) | 0.092 | 1166 |
| **Diabetes** | 136 (11.6%) | 118 (11.5%) | 18 (12.4%) | 0.852 | 1172 |
| **Anti-hypertensive drugs** | | | | | |
| **ACE inhibitor:** | 277 (23.6%) | 245 (23.8%) | 32 (22.4%) | 0.790 | 1173 |
| **Aspirin:** | 352 (30.1%) | 325 (31.6%) | 27 (18.9%) | 0.003 | 1171 |
| **Betablocker:** | 530 (45.1%) | 472 (45.8%) | 58 (40.6%) | 0.277 | 1174 |

**e-Table 6.** Contributors to the predictive classifiers in TAV

| GLM | | StepAIC (top 10) | | Lasso (top 10) | | RF (top 10) | | Neural network (top 10 ) | |
|---|---|---|---|---|---|---|---|---|---|
| **AS** | 100.000 | **AS** | 100.000 | **AS** | 100.000 | **AS** | 100.000 | **AS** | 100.00 |
| **age** | 27.762 | **AI** | 70.043 | **Diabetes** | 39.6265 | **AI** | 23.581 | **age** | 62.81 |
| **Diabetes** | 27.003 | **age** | 41.852 | **Renal Failure** | 26.2556 | **Creatinine** | 21.571 | **Diabetes** | 47.28 |
| **AI** | 21.226 | **LDL** | 31.027 | **Father Stroke o r Mi or TIA65** | 16.4437 | **age** | 21.181 | **Beta blocker** | 41.58 |
| **BSA** | 17.970 | **Hemoglobin** | 27.641 | **MI** | 15.3655 | **PP** | 16.418 | **Creatinine** | 38.18 |
| **Pulse BP** | 16.595 | **ASA** | 26.802 | **ALAT** | 14.7816 | **Leukocytes** | 14.558 | **FatherMI65** | 35.95 |
| **LDL** | 8.753 | **Cholesterol** | 26.657 | **Sibling AP65** | 14.7506 | **hsCRP** | 13.549 | **Diastolic BP** | 34.47 |
| **Gender** | 7.564 | **Diabetes** | 26.541 | **HF** | 12.8772 | **Systolic BP** | 13.540 | **Deep VTPEM** | 33.27 |
| **hsCRP** | 0.000 | **hsCRP** | 22.648 | **Father MI65** | 12.4264 | **Height** | 13.199 | **Father Stroke or Mi or TIA65** | 33.16 |

**e-Figure 1:** Flowchart of the study

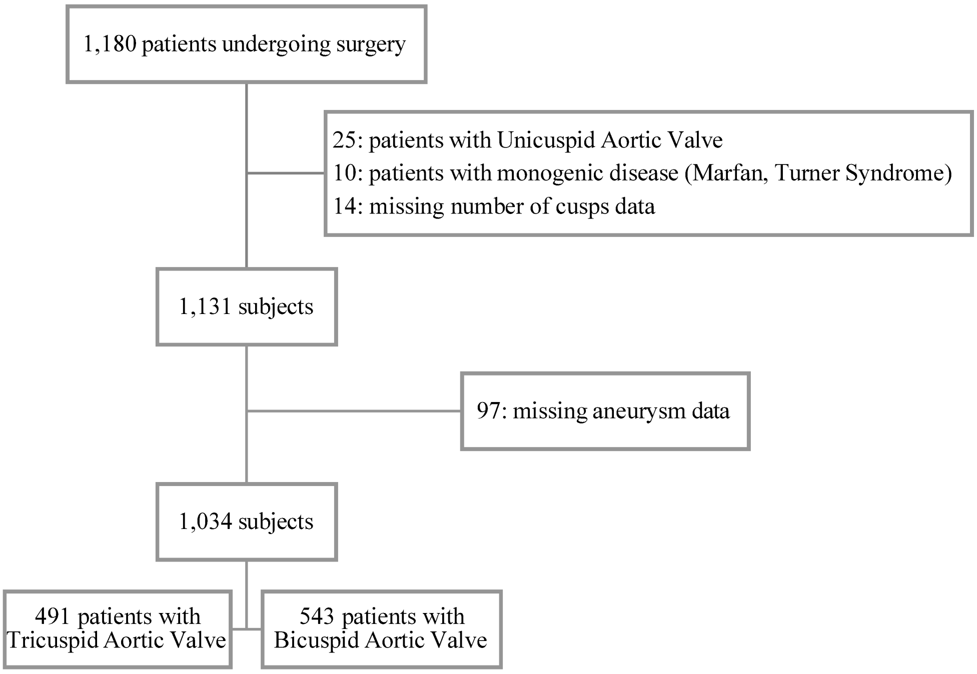

**e-Figure 2.** Plot of methodology used to choose variables

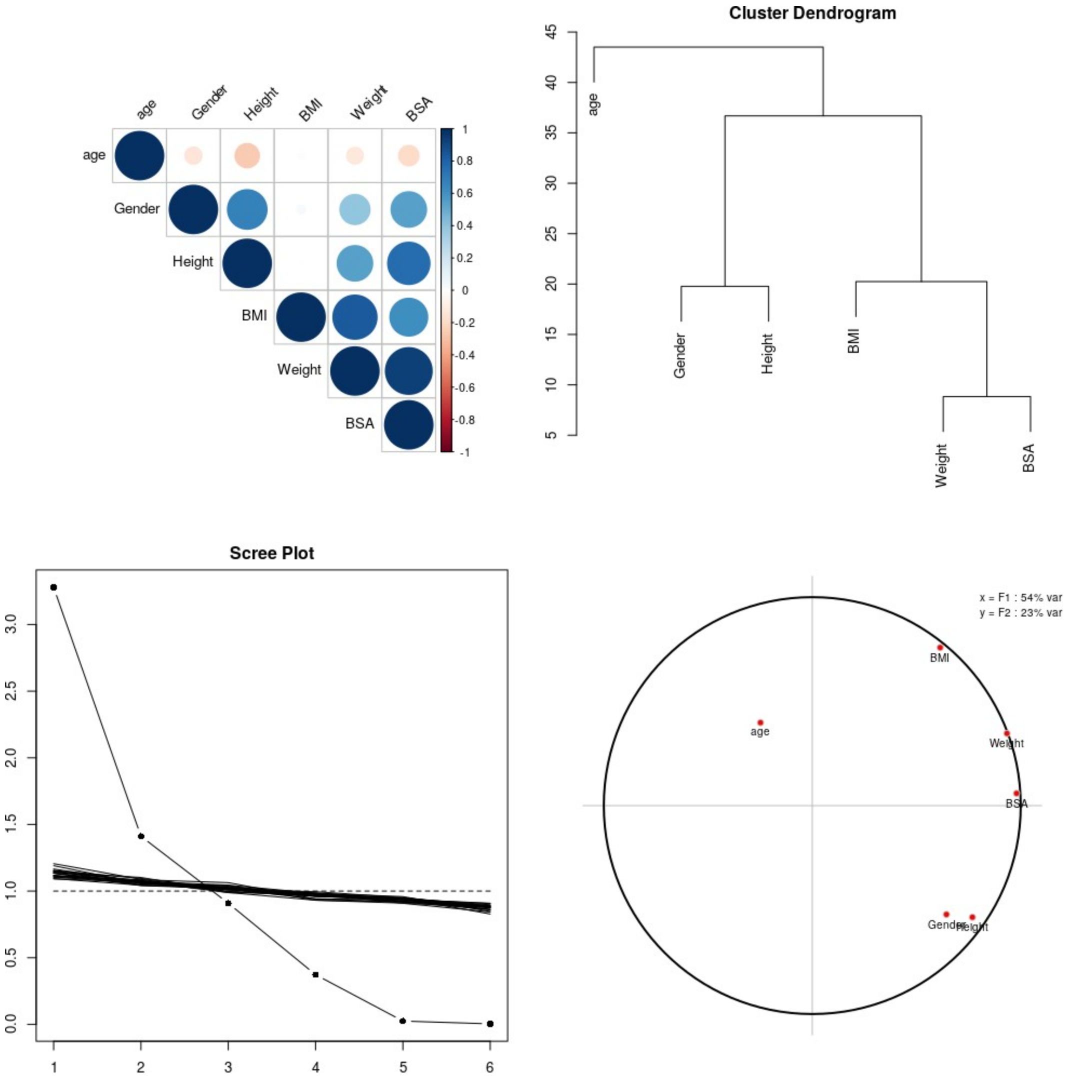

**e-Figure 3.** The ROC curve for our risk prediction model across the predictive methods using imputed data

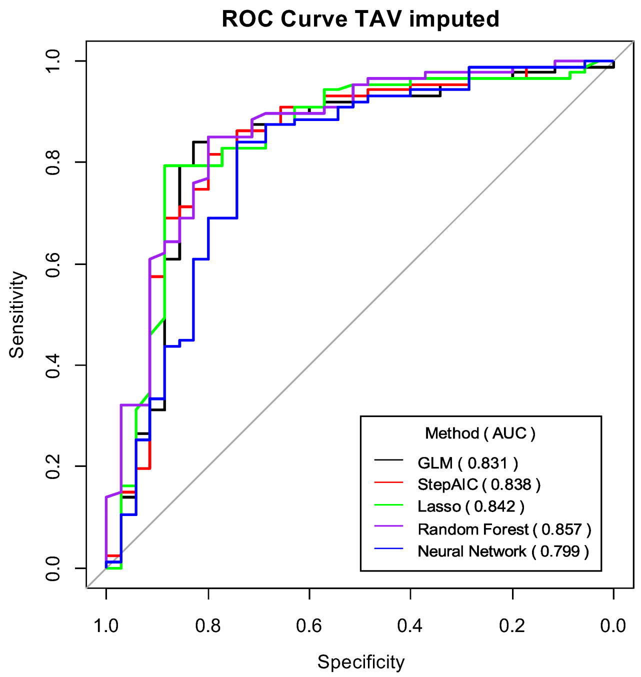


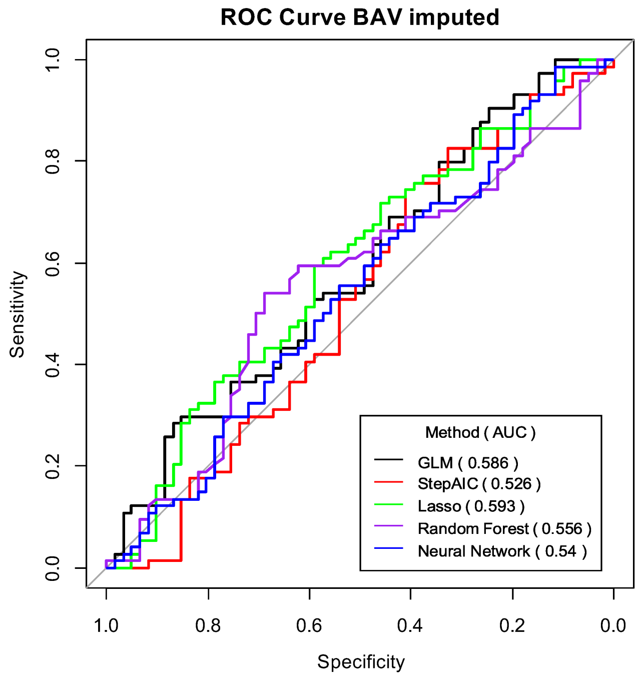

**e-Figure 4.** Principal component analysis (PCA) plot of TAV patients with or without aneurysm stratified by AS

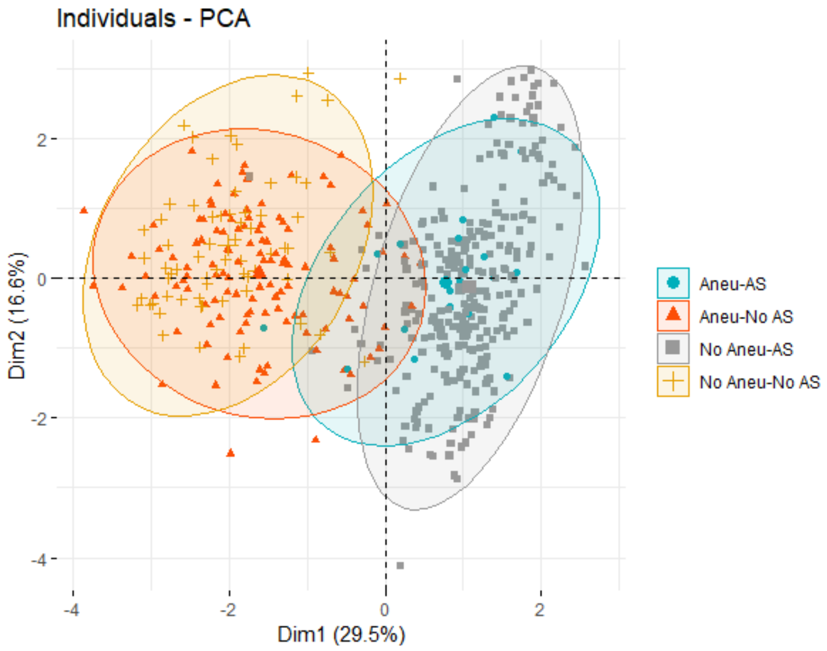

**e-Figure 5.** The ROC curve for our risk prediction model across the predictive methods without AS as predictor

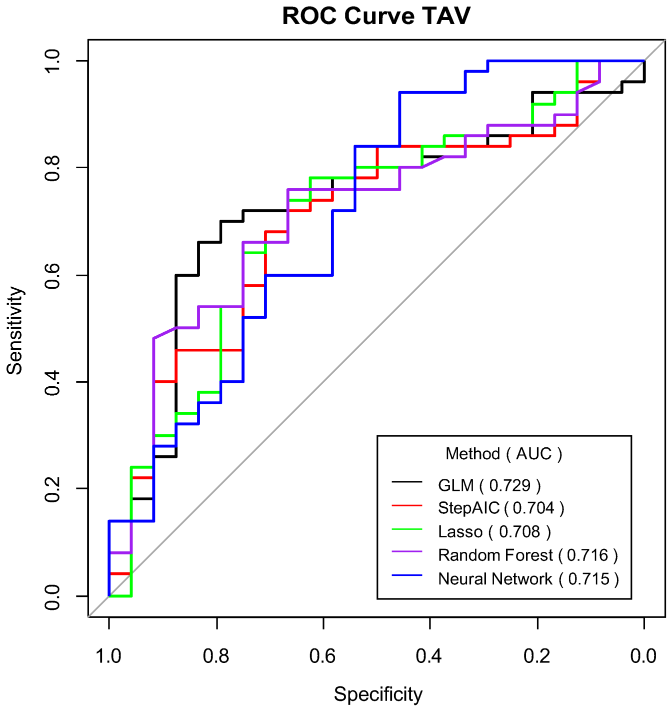


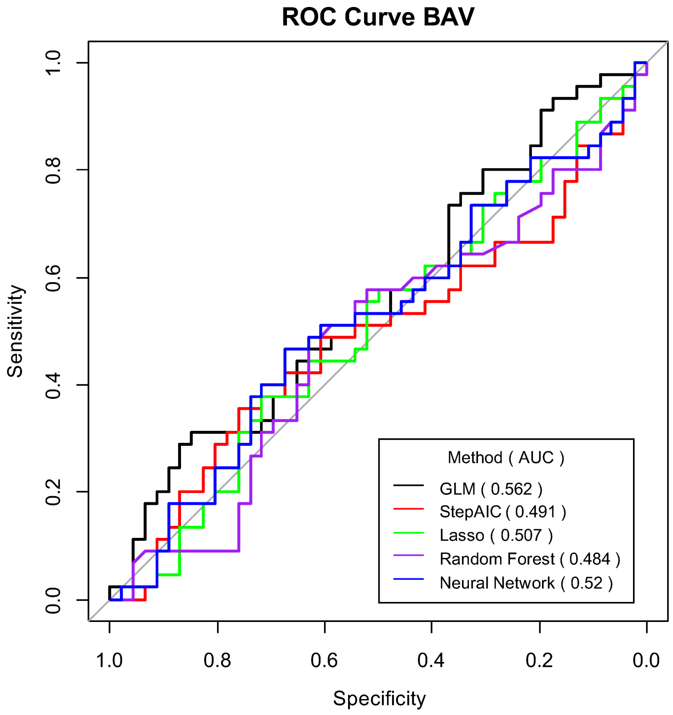

***Measurements and definitions***

Hypertension persons are defined as persons whose blood pressure measurement at the current year's health check-up is ≥ 140 mmHg systolic (1 mmHg = 0.1333 kPa) and/or 90 mmHg diastolic, or who have been diagnosed as hypertensive (1).

Diabetic patients are defined as patients whose fasting blood glucose is currently controlled at < 7.0 mmol/L or whose glycated haemoglobin is < 7% (2).

Dyslipidemia: those with triglycerides ≥ 2.26 mmol/L and/or total cholesterol ≥ 6.22 mmol/L and/or HDL cholesterol < 1.04 mmol/L and/or LDL cholesterol ≥ 4.14 mmol/L (3).

*1. Mancia G, Kreutz R, Brunström M, Burnier M, Grassi G, Januszewicz A, Muiesan ML, Tsioufis K, Agabiti-Rosei E, Algharably EAE, Azizi M, Benetos A, Borghi C, Hitij JB, Cifkova R, Coca A, Cornelissen V, Cruickshank JK, Cunha PG, Danser AHJ, Pinho RM, Delles C, Dominiczak AF, Dorobantu M, Doumas M, Fernández-Alfonso MS, Halimi JM, Járai Z, Jelaković B, Jordan J, Kuznetsova T, Laurent S, Lovic D, Lurbe E, Mahfoud F, Manolis A, Miglinas M, Narkiewicz K, Niiranen T, Palatini P, Parati G, Pathak A, Persu A, Polonia J, Redon J, Sarafidis P, Schmieder R, Spronck B, Stabouli S, Stergiou G, Taddei S, Thomopoulos C, Tomaszewski M, Van de Borne P, Wanner C, Weber T, Williams B, Zhang ZY, Kjeldsen SE. 2023 ESH Guidelines for the management of arterial hypertension The Task Force for the management of arterial hypertension of the European Society of Hypertension: Endorsed by the International Society of Hypertension (ISH) and the European Renal Association (ERA). J Hypertens. 2023 Dec 1;41(12):1874-2071. doi: 10.1097/HJH.0000000000003480. Epub 2023 Sep 26. PMID: 37345492.*

*2. Marx N, Federici M, Schütt K, Müller-Wieland D, Ajjan RA, Antunes MJ, Christodorescu RM, Crawford C, Di Angelantonio E, Eliasson B, Espinola-Klein C, Fauchier L, Halle M, Herrington WG, Kautzky-Willer A, Lambrinou E, Lesiak M, Lettino M, McGuire DK, Mullens W, Rocca B, Sattar N; ESC Scientific Document Group. 2023 ESC Guidelines for the management of cardiovascular disease in patients with diabetes. Eur Heart J. 2023 Oct 14;44(39):4043-4140. doi: 10.1093/eurheartj/ehad192. Erratum in: Eur Heart J. 2023 Dec 21;44(48):5060. PMID: 37622663.*

*3. Mach F, Baigent C, Catapano AL, Koskinas KC, Casula M, Badimon L, Chapman MJ, De Backer GG, Delgado V, Ference BA, Graham IM, Halliday A, Landmesser U, Mihaylova B, Pedersen TR, Riccardi G, Richter DJ, Sabatine MS, Taskinen MR, Tokgozoglu L, Wiklund O; ESC Scientific Document Group. 2019 ESC/EAS Guidelines for the management of dyslipidaemias: lipid modification to reduce cardiovascular risk. Eur Heart J. 2020 Jan 1;41(1):111-188. doi: 10.1093/eurheartj/ehz455. Erratum in: Eur Heart J. 2020 Nov 21;41(44):4255. PMID: 31504418.*
