# Supplementary material for: Predictive machine learning models for ascending aortic dilatation in patients with bicuspid and tricuspid aortic valves undergoing cardiothoracic surgery: a prospective, single-centre and observational study
Source: BMJ Open. 2024 Mar 20;14(3):e067977. doi: 10.1136/bmjopen-2022-067977 (PMC10961501; doi:10.1136/bmjopen-2022-067977)
Supplement: Author's manuscript [file bmjopen-2022-067977.draft_revisions.pdf]

BMJ Open is committed to open peer review. As part of this commitment we make the peer review history of every article we publish publicly available.

When an article is published we post the peer reviewers' comments and the authors' responses online. We also post the versions of the paper that were used during peer review. These are the versions that the peer review comments apply to.

The versions of the paper that follow are the versions that were submitted during the peer review process. They are not the versions of record or the final published versions. They should not be cited or distributed as the published version of this manuscript.

BMJ Open is an open access journal and the full, final, typeset and author-corrected version of record of the manuscript is available on our site with no access controls, subscription charges or pay-per-view fees (http://bmjopen.bmj.com).

If you have any questions on BMJ Open's open peer review process please email info.bmjopen@bmj.com

BMJ Open

Clinical classifiers to identify ascending aortic dilatation in patients with bicuspid versus tricuspid aortic valves

Journal:	*BMJ Open*
Manuscript ID	bmjopen-2022-067977
Article Type:	Original research
Date Submitted by the Author:	07-Sep-2022
Complete List of Authors:	Gaye, Bamba; INSERM U970, ; Universite de Paris Faculte de Sante,<br>Vignac, Maxime; Karolinska Institutet, Cardiovascular Medicine Unit, Center for Molecular Medicine, Department of Medicine<br>Gådin, Jesper R.; Karolinska Institutet, Cardiovascular Medicine Unit, Center for Molecular Medicine, Department of Medicine<br>Ladouceur, Magalie; INSERM; Georges Pompidou European Hospital Anatomical Pathology<br>Caidahl, Kenneth; Karolinska Institutet, Clinical Physiology Unit, Department of Molecular Medicine and Surgery<br>Olsson, Christian; Karolinska Institutet, Cardiothoracic Surgery Unit, Department of Molecular Medicine and Surgery<br>Franco-Cereceda, Anders; Karolinska Institutet, Cardiothoracic Surgery Unit, Department of Molecular Medicine and Surgery<br>Eriksson, Per ; Karolinska Institutet, Cardiovascular Medicine Unit, Center for Molecular Medicine, Department of Medicine<br>Björck, Hanna; Karolinska Institutet, Cardiovascular Medicine Unit, Center for Molecular Medicine, Department of Medicine
Keywords:	Valvular heart disease < CARDIOLOGY, EPIDEMIOLOGY, Cardiology < INTERNAL MEDICINE

SCHOLARONE™
Manuscripts

I, the Submitting Author has the right to grant and does grant on behalf of all authors of the Work (as defined in the below author licence), an exclusive licence and/or a non-exclusive licence for contributions from authors who are: i) UK Crown employees; ii) where BMJ has agreed a CC-BY licence shall apply, and/or iii) in accordance with the terms applicable for US Federal Government officers or employees acting as part of their official duties; on a worldwide, perpetual, irrevocable, royalty-free basis to BMJ Publishing Group Ltd ("BMJ") its licensees and where the relevant Journal is co-owned by BMJ to the co-owners of the Journal, to publish the Work in this journal and any other BMJ products and to exploit all rights, as set out in our licence.

The Submitting Author accepts and understands that any supply made under these terms is made by BMJ to the Submitting Author unless you are acting as an employee on behalf of your employer or a postgraduate student of an affiliated institution which is paying any applicable article publishing charge ("APC") for Open Access articles. Where the Submitting Author wishes to make the Work available on an Open Access basis (and intends to pay the relevant APC), the terms of reuse of such Open Access shall be governed by a Creative Commons licence – details of these licences and which Creative Commons licence will apply to this Work are set out in our licence referred to above.

Other than as permitted in any relevant BMJ Author's Self Archiving Policies, I confirm this Work has not been accepted for publication elsewhere, is not being considered for publication elsewhere and does not duplicate material already published. I confirm all authors consent to publication of this Work and authorise the granting of this licence.

For peer review only

Clinical classifiers to identify ascending aortic dilatation in patients with bicuspid versus tricuspid aortic valves

Bamba Gaye[1*] (PhD), Maxime Vignac[1] (MSc), Jesper R. Gådin[1] (PhD), Magalie Ladouceur[2,3] (MD), Kenneth Caidahl[4] (MD, PhD), Christian Olsson[5] (MD, PhD), Anders Franco-Cereceda[5] (MD), Per Eriksson[1] (PhD), Hanna M. Björck[1*] (PhD)

Running Title: Clinical Classifiers in BAV versus TAV

Total Word Count: 3315

Affiliations:
[1]Cardiovascular Medicine Unit, Center for Molecular Medicine, Department of Medicine, Karolinska Institutet, Stockholm, Karolinska University Hospital, Solna, Sweden
[2]INSERM, U970, Paris Cardiovascular Research Center / Université Paris Descartes, Sorbonne Paris Cité, Faculté de Médecine, Paris, France
[3]AP-HP, Georges Pompidou European Hospital, Cardiology Department, Paris, France
[4]Clinical Physiology Unit, Department of Molecular Medicine and Surgery, Karolinska Institutet, Stockholm, Sweden
[5]Cardiothoracic Surgery Unit, Department of Molecular Medicine and Surgery, Karolinska Institutet, Stockholm, Sweden

Disclosure: All authors have nothing to disclose.

***Address for Correspondence:**
Dr. Bamba Gaye (email: bamba.gaye@inserm.fr)
Dr. Hanna Bjorck (email: hanna.bjorck@ki.se)
Cardiovascular Medicine Unit, BioClinicum J8:20, Visionsgatan 4, Karolinska University Hospital, SE-171 64 Solna, Sweden

Key Points

Questions: Neither the underlying pathophysiology nor a valid prediction model of the development of the aneurysm is available in patients with bicuspid aortic valves (BAV).

Findings: Using automated machine learning algorithms and classic logistic regression models we demonstrated that clinical predictors can predict ascending aortic dilatation in patients with tricuspid aortic valves (TAV) but not in patients with bicuspid aortic valves (BAV).

Meaning: The lack of good models to develop clinical classifiers of BAV-associated ascending aortic dilatation strengthen the focus of genetics and/or flow as important contributing factors to aneurysm development in these individuals.

For peer review only

Abstract

Objective: We aimed to develop clinical classifiers to identify prevalent ascending aortic dilatation in patients with BAV and tricuspid aortic valve (TAV).

Methods: This study included BAV (n=543) and TAV (n=491) patients with aortic valve disease and/or ascending aortic dilatation but devoid of coronary artery disease undergoing cardiothoracic surgery. We applied machine learning algorithms and classic logistic regression models, using multiple variable selection methodologies to identify predictors of high risk of ascending aortic dilatation (ascending aorta with a diameter above 40 mm). Analyses included comprehensive multidimensional data (i.e., valve morphology, clinical data, family history of cardiovascular diseases, prevalent diseases, demographic, lifestyle and medication).

Results: BAV patients were younger (60.4 ± 12.4 years) than TAV patients (70.4±9.1 years), and had a higher frequency of aortic dilatation (45.3% vs. 28.9% for BAV and TAV, respectively. P<0.001). The aneurysm prediction models showed mean AUC values above 0.8 for TAV patients, with the absence of aortic stenosis being the main predictor, followed by diabetes and high sensitivity C-Reactive Protein. Using the same clinical meaures in BAV patients our prediction model resulted in AUC values between 0.5-0.55, not useful for prediction of aortic dilatation. The classiffication results were consistent for all machine learning algorithms and classic logistic regression models.

Conclusions: Cardiovascular risk profiles appear to be more predictive of aortopathy in TAV patients than in patients with BAV. This adds evidence to the fact that BAV- and TAV-associated aortopathy involve different pathways to aneurysm formation and highlights the need for specific aneurysm preventions in these patients. Further, our results highlight that machine learning approaches do not outperform classical prediction methods in addressing complex interactions and non-linear relations between variables.

Keywords: Bicuspid Aortic Valve; Tricuspid Aortic Valve; Ascending Aortic Aneurysm; Aortic Dilation

For peer review only

Abbreviations

AAA	Abdominal Aortic Aneurysm
AR	Aortic Regurgitation
AS	Aortic Stenosis
ASAP	Advanced Study of Aortic Pathology
BAV	Bicuscpid Aortic Valve
BMI	Body Mass Index
BSA	Body Surface Area
CVD	Cardiovascular Disease
DAVAACA	Disease of the Aortic Valve Ascending Aorta and Coronary Arteries
FFR	Fractional Flow Reserve
Hs-CRP	High Sensitivity C-Reactive Protein
LDL	Low Density Lipoprotein
LN	Left- and Non-Coronary Cusps
MCA	Multiple Correspondence Analysis
PCA	Principal ComponentAanalysis
PP	Pulse Pressure
TAA	Thoracic Aortic Aneurysm
TAV	Tricuspid Aortic Valve
TEE	Transesophageal Echocardiography
TTE	Transthoracic Echocardiography

For peer review only

Introduction

Thoracic aortic aneurysm (TAA) is a silent disease characterized by medial degradation and pathological widening of the intrathoracic aorta. Individuals with a bicuspid aortic valve (BAV) are at higher risk of developing ascending aortic aneurysm than individuals born with a normal tricuspid aortic valve (TAV).[1,2] BAV is the most common congenital cardiac abnormality with an incidence of about 13 per 1000 live births and more than one third of all BAV patients develop aneurysm or aortic dissection later in life.[3–5] The underlying mechanism is not known although a turbulent flow, caused by the abnormal bicuspid valve anatomy, and/or a genetic defect have been proposed as contributing factors. The complications of BAV have led to a number of unanswered clinical questions such as: (1) what mechanisms are underlying the development of aortopathy in adults with BAV, (2) how to predict the development of aortopathy in individuals with BAV, and (3) will prediction models performance and predictors be different in patients with BAV compared to patients with TAV. Echocardiography is effective to show the degree of the sinus valsava and the annulus dilatation. However, it is more difficult to know the degree of dilation of the sinotubular junction and the ascending aorta even though they are the more frequently dilatated sections in patients with BAV[6].

We aim was to identify clinical classifiers of prevalent aortopathy using comprehensive multidimensional clinical data. Automated machine learning methods and traditional regression models was performed on a total of 1034 subjects with either BAV or TAV.

For peer review only

METHODS

Clinical cohorts

The Advanced Study of Aortic Pathology (ASAP) study design and description have been previously published.[1,7] Briefly, the ASAP cohort is a single-center, observational cohort study of consecutive patients with aortic valve and/or ascending aortic disease undergoing elective open-heart surgery at the Cardiothoracic Surgery Unit, Karolinska University Hospital in Stockholm, Sweden. Inclusion criteria were patients aged 18 or above with aortic valve disease (i.e., aortic stenosis; AS or aortic regurgitation; AR) and/or ascending aorta dilatation (aneurysm or ectasia) but devoid of significant coronary artery disease (stenosis >70% or Fractional Flow Reserve (FFR) <0.80) and primarily not planned for another concomitant valve surgery. The Disease of the Aortic Valve Ascending Aorta and Coronary Arteries (DAVAACA) study was set up in the continuity of the ASAP study. The ASAP/DAVAACA study started in February 2007 and include multidimensional data (blood analyses, genetic and clinical data, family history of cardiovascular diseases, prevalent diseases data, demographic characteristics, lifestyle habits data and medication). In the present study, 1180 operated patients were included (June 28, 2017). Exclusion of patients with unicuspid aortic valves, patients with missing data for cuspidity and aortic dilatation and patients with syndromic forms of TAA resulted in a final study population of 1034 subjects (e-Figure 1). Data collection and classification of AS and AR have been previously described.[1] Aortic valve function and aortic dimensions were assessed according to the standards outlined by the American Society of Echocardiography.[8–10]

For peer review only - http://bmjopen.bmj.com/site/about/guidelines.xhtml

Echocardiography

Preoperative transthoracic echocardiography (TTE) was used to determine aortic valve function. Transesophageal echocardiography (TEE) was performed on the operating table, prior to surgery, under general anaesthesia and as previously described.[11] The echocardiographic evaluation has been already pulished elsewhere.[1] TEE evaluation was used to assess valvular function, aortic root morphology and ascending aortic diameter; measurements were performed according to standards outlined by the ASE. Diameter of the ascending aorta was measured four centimeters above the aortic sinotubular junction. An ascending aorta with a diameter above 40 mm was classified as arotic dilatation in both women and men. Body surface area (BSA) was calculated using Du Bois method.[12] Aortic stenosis and aortic regurgitation were difined according to standard guidelines for valve surgery.[13]

Aortic valve morphology

The morphology of the aortic valve was determined by inspection of the valve during surgery. Based on appearance, the valve was classified according to number of cusps and commissures. Three cusps and three commissures denote a TAV; two cusps and two commissures denote a BAV (if a remnant commissural raphe was present) or true BAV (if no raphe was present). The BAV was further classified according to cusps fusion; right- and left coronary cusps (RL), right- and non-coronary cusps (RN) or left- and non-coronary cusps (LN).[1] The Sievers classification[14], based on intraoperative analysis of the aortic valve morphology, was used to diagnose and type the bicuspid aortic valve.

Statistical analysis

Characteristics of the population were described using ANOVA or Chi-square tests when appropriate. Several meaningful blocks of variables were chosen (clinical data, family history

For peer review only - http://bmjopen.bmj.com/site/about/guidelines.xhtml

of cardiovascular diseases, prevalent diseases, demographic characteristics, lifestyle habits data and medication). Principal component analysis (PCA) and Multiple correspondence analysis (MCA) were used to explain the variance-covariance structure of the variables combined. To identify variables that separated dilated versus non-dilated BAV and TAV patients, logistic regression models were used and automated machine learning algorithms were applied.

Logistic Regression

For logistic regression, a set of variables were selected to be included in the model. Since model performance can rely heavily on variable selection, different variable selection methods were tested prior to the logistic regression analysis (e-Figure 2). Firstly, variables were selected based on prior knowledge and/or biologic plausibility. Then, two automated variable selection methods were considered: (i) backward and forward elimination to optimize Akaike Information Criteria[11], (ii) and selection operator (LASSO).[12]

Machine Learning Algorithms

We used two machine learning algorithms, Random Forests and Artificial Neural Network, which are among the most widely and successfully used for clinical data.[15,16] Each one of them represents a different algorithm "family", with different internal algorithm structures.[17] Since it was not known beforehand which kind of algorithm would perform best, algorithms with different internal structures were chosen to increase the probability of good discriminative performance. A 10-fold cross validation method to our logistic regression was also applied, allowing to split the training data into five subsets. Then, five times iteration over the five subsets were performed, such that the subset with the index same as the iteration number was used as the validation test and the remaining 4 subsets were training tests. Sensitivity,

For peer review only - http://bmjopen.bmj.com/site/about/guidelines.xhtml

specificity, and positive and negative predictive values for each predictive model were also assessed, applying a leave one out cross validation method (e-Figure 3, e-Figure 4).

Patient and Public Involvement statement

It was not possible to involve patients or the public in the design, or conduct, or reporting, or dissemination plans of our research.

RESULTS

Characteristics of BAV and TAV patients

The study population included a total of 1034 patients (52% BAV and 48% TAV) (e-Table 1, e-Figure 1). BAV patients were significantly younger (60.4 (SD 12.40) years vs. 70.4 (SD 9.09) years in TAV patients), and mainly men (73.1%) compared to TAV patients (63.3%). Furthermore, BAV patients had significantly higher body surface area (BSA) but, in contrast, a lower body mass index (BMI). AS was more common in BAV compared to TAV whereas no significant difference in the proportion of AR was found, even if the prevalence of AR was slightly higher in TAV patients. Finally, a higher prevalence of cardiovascular disease (CVD) history was observed in TAV individuals (hypertension, coronary artery disease, heart failure and stroke) and a higher percentage of family history of CVD (sibling history of myocardial infarction before 65 years of age).

For peer review only

Table 1: Baseline Characteristics of Patients with BAV or TAV (dilated vs. non-dilated ascending aorta).

	BAV patients (n=543)			TAV patients (n=491)		
	Dilated (n=246)	Non-Dilated (n=297)	P value	Dilated (n=142)	Non-Dilated (n=349)	P value
**Gender: Male**	182 (74.0%)	215 (72.4%)	0.749	89 (62.7%)	222 (63.6%)	0.927
**Age**	61.1 (11.2)	59.8 (13.3)	0.204	67.2 (10.1)	71.8 (8.28)	<0.001
**Height**	177 (9.14)	174 (9.29)	0.002	174 (9.76)	172 (9.77)	0.043
**Weight**	83.1 (15.6)	80.9 (15.8)	0.100	81.4 (16.3)	81.6 (15.7)	0.909
**BSA**	2.00 (0.21)	1.96 (0.22)	0.052	1.95 (0.22)	1.94 (0.22)	0.479
**BMI**	26.5 (4.15)	26.6 (4.36)	0.916	26.9 (4.41)	27.7 (4.90)	0.087
**Regular Smoker:**			0.176			0.537
**No**	108 (44.1%)	142 (48.8%)		61 (43.3%)	152 (43.8%)	
**Former**	117 (47.8%)	117 (40.2%)		69 (48.9%)	177 (51.0%)	
**Yes**	20 (8.16%)	32 (11.0%)		11 (7.80%)	18 (5.19%)	
**Raphe:**			0.568			
**True BAV**	24 (9.96%)	23 (7.96%)				
**Left-Non coronary**	1 (0.41%)	4 (1.38%)				
**Right-Non coronary**	43 (17.8%)	47 (16.3%)				
**Right-Left**	173 (71.8%)	215 (74.4%)				
**Systolic Blood Pressure**	135 (18.2)	136 (18.9)	0.299	144 (17.4)	142 (21.5)	0.299
**Diastolic Blood Pressure**	81.3 (12.3)	79.0 (11.6)	0.026	75.1 (13.0)	76.5 (13.3)	0.290
**Pulse Pressure (PP)**	53.3 (16.5)	57.2 (18.3)	0.009	68.7 (18.1)	65.4 (20.3)	0.076
**Leukocytes**	5.70 [4.70;6.80]	5.90 [4.97;7.00]	0.052	6.00 [5.00;7.30]	6.10 [5.10;7.40]	0.386
**hsCRP**	0.93 [0.50;2.15]	1.20 [0.59;2.80]	0.056	2.10 [0.98;4.05]	1.60 [0.71;3.80]	0.050
**Cholesterol**	4.90 [4.20;5.70]	4.80 [4.20;5.60]	0.566	4.80 [4.12;5.40]	4.60 [3.90;5.60]	0.175
**LDL**	2.90 [2.20;3.60]	2.80 [2.30;3.60]	0.813	2.90 [2.20;3.50]	2.60 [1.90;3.40]	0.064
**Aortic Stenosis:**	169 (68.7%)	235 (79.1%)	0.008	23 (16.2%)	283 (81.1%)	<0.001
**Aortic Regurgitation:**	83 (33.7%)	75 (25.3%)	0.038	93 (65.5%)	67 (19.2%)	<0.001
**Diameter Aortic Anulus**	25.5 (3.41)	24.2 (3.58)	<0.001	23.5 (3.17)	22.8 (3.03)	0.042
**Diameter Sinus Valsalva**	38.1 (5.38)	33.6 (5.11)	<0.001	40.1 (6.98)	32.8 (5.71)	<0.001
**Diameter STJ**	33.6 (5.63)	28.2 (4.51)	<0.001	35.9 (6.76)	26.6 (4.36)	<0.001
**Diameter Ascend Aorta**	46.9 (5.02)	33.0 (4.27)	<0.001	50.5 (7.90)	31.6 (4.03)	<0.001
**Sibling MI before 65 year**	13 (5.56%)	19 (6.81%)	0.688	11 (8.09%)	35 (10.8%)	0.474
**Mother MI before 65 year**	15 (6.20%)	10 (3.64%)	0.250	7 (5.26%)	25 (7.67%)	0.474
**Father MI before 65 year**	24 (10.6%)	45 (16.3%)	0.087	18 (14.1%)	45 (14.4%)	0.922
**Myocardial Infarction**	8 (3.25%)	11 (3.77%)	0.930	9 (6.43%)	33 (9.46%)	0.367
**Stroke**	15 (6.10%)	15 (5.08%)	0.746	14 (10.0%)	43 (12.3%)	0.571
**AAA**	23 (9.39%)	2 (0.68%)	<0.001	25 (18.2%)	5 (1.44%)	<0.001
**Angina Pectoris**	9 (3.66%)	26 (8.87%)	0.023	9 (6.43%)	52 (14.9%)	0.015
**Heart Failure**	15 (6.15%)	25 (8.65%)	0.354	15 (10.6%)	42 (12.2%)	0.747
**Hypertension**	115 (47.1%)	140 (47.6%)	0.979	88 (62.9%)	223 (65.0%)	0.730
**Diabetes**	15 (6.10%)	39 (13.3%)	0.009	3 (2.16%)	61 (17.5%)	<0.001
**ACE inhibitor**	46 (18.7%)	57 (19.3%)	0.941	45 (31.9%)	97 (27.9%)	0.434
**Aspirin**	48 (19.5%)	84 (28.5%)	0.021	38 (27.0%)	155 (44.8%)	0.001
**Betablocker**	92 (37.4%)	125 (42.4%)	0.277	76 (53.9%)	179 (51.3%)	0.672

Chi square test was used for categorical variables; ANOVA for normal-distributed continuous variables; Kruskal-Wallis test for non-normal continuous variables. BSA, body surface area; BMI, body mass index; hsCRP, high sensitive C-reactive protein; LDL, low density lipoprotein; STJ, sinotubular junction; AAA, abdominal aortic aneurysm.

For peer review only - http://bmjopen.bmj.com/site/about/guidelines.xhtml

Aortic dilatation in BAV and TAV patients

Globally, PCA showed no separation among BAV patients with (BAV-D) or without (BAV-ND) ascendic aortic dilatation (Figure 1, Panel A), using general clinical characteristics, family history of CVD, prevalent diseases and demographics as input. By contrast, TAV patients with a dilated (TAV-D) ascending aorta clearly separated from TAV individuals with non-dilated (TAV-ND) ascending aortas (Figure 1, Panel B), indicating a more pronounced difference between these groups.

Analysis of patient descriptive data showed a pattern of significantly different associated traits between BAV-ND vs. BAV-D and TAV-ND vs. TAV-D (Table 1) patients. Specifically, TAV-D patients were younger than TAV-ND patients while in the BAV group age did not differ. BAV-D patients, on the other hand, had a higher BSA and diastolic blood pressure, and a lower pulse pressure (PP) than BAV-ND patients. In both BAV and TAV patients, AR was more commonly associated with aortic dilatation, whilst AS was more prevalent in patients with non-dilated aorta. It was also noted that aortic dilatation was more localized to the tubular part of the ascending aorta in BAV patients, whereas in TAV patients the dilatation more often included the aortic root (Table 1). A positive association between dilatation and abdominal aortic aneurysm (AAA), and negative associations between dilatation and diabetes, and dilatation and angina was found in both BAV and TAV patients (Table 1).

To further analyze BAV- and TAV-associated aortopathy, a multivariable logistic regression model was performed, including age, sex, BSA, low density lipoprotein (LDL) cholesterol and high sensitivity C-Reactive Protein (hs-CRP) as covariates (Table 2). Independent variables were choosen based on previous analyses with significant differences within groups. Covariates were selected based on stepwise selection and decision trees. The results showed that in BAV

For peer review only - http://bmjopen.bmj.com/site/about/guidelines.xhtml

patients, AS, PP and diabetes were negatively associated with dilatation (adjusted models for age, sex, BSA, LDL and hsCRP were with AS: OR=0.44 (0.28-0.70), P=0.001; PP: OR=0.99 (0.98-1.0), P=0.025, and diabetes: OR=0.32 (0.16-0.62), P=0.001). Similarly, in TAV patients, dilatation was negatively associated with AS: OR=0.03 (0.02-0.06), P<0.001 and diabetes OR=0.11 (0.03-0.32), P<0.001. The magnitude of the association with AS was however 15-fold higher in TAV than in BAV patients. Aortic regurgitation was positively associated with aortic dilation in both BAV and TAV with a 5-fold higher magnitude in TAV compared to BAV patients. (Table 2).

For peer review only

For peer review only - http://bmjopen.bmj.com/site/about/guidelines.xhtml

Table 2. Predictors of ascending aortic dilatation in BAV and TAV patients, separately. Univariate and Multivariate logistic regression analysis.

	BAV						TAV					
	unAdjusted Model		Model 1		Model 2		unAdjusted Model		Model 1		Model 2	
	OR (95% IC)*	p	OR (95% IC)*	p	OR (95% IC)*	p	OR (95% IC)*	p	OR (95% IC)*	p	OR (95% IC)*	p
**Aortic Stenosis**	0.58 (0.39-0.85)	0.006	0.44 (0.28-0.70)	0.001	0.46 (0.24-0.88)	0.020	0.05 (0.03-0.07)	<0.001	0.03 (0.02-0.06)	<0.001	0.02 (0.01-0.05)	<0.001
**Aortic Regurgitation**	1.51 (1.04-2.19)	0.031	1.92 (1.25-2.99)	0.003	1.42 (0.76-2.66)	0.270	7.99 (5.19-12.45)	<0.001	7.47 (4.62-12.27)	<0.001	0.58 (0.23-1.36)	0.229
**Pulse Pressure**	0.99 (0.98-1.00)	0.011	0.99 (0.98-1.00)	0.025	0.98 (0.97-0.99)	0.001	1.01 (1.00-1.02)	0.091	1.00 (0.99-1.02)	0.385	0.99 (0.97-1.00)	0.096
**Diabetes**	0.42 (0.22-0.77)	0.007	0.32 (0.16-0.62)	0.001	0.36 (0.18-0.72)	0.005	0.1 (0.03-0.29)	<0.001	0.11 (0.03-0.32)	<0.001	0.26 (0.06-0.87)	0.048

* Odds ratio and 95% confidence interval limits were obtained by logistic regression
Model 1: adjusted for Sex_Male + Age + BSA + LDL + hsCRP
Model 2: adjusted for Sex_Male + Age + BSA + LDL+ hsCRP + the 3 other variables (i.e: Aortic Regurgitation, Pulse Pressure and Diabetes for Aortic Stenosis)

For peer review only

Clinical classifiers of aortic dilatation

In order to answer the question if the prevalence of aortopathy in TAV and BAV patients may be identified using clinical characteristics, automated machine learning algorithms, including Random Forests and Artificial Neural Network, and classic logistic regression models were used. This showed that the discrimination between TAV-ND and TAV-D patients was good for all models tested, with mean AUCs ranging from 0.81 to 0.88 (Figure 2, e-Table 2). The best discrimination was obtained using Random Forest (mean AUC: 0.88 (95% CI: 0.78-0.96)) and GLM (mean AUC: 0.81 (95% CI: 0.68-0.93)) (Figure 2, e-Table 2). Generalized Linear Regressions included AS, AR, PP, prevalence of diabetes, age, sex, BSA, LDL, hsCRP, BMI as variables. It was not possible to predict aortic dilatation in BAV individuals that are at high risk of developing aneurysm, as indicated by poor discrimination by all classifications models used (Figure 2, e-Table 2). This is in line with the lack of separation in the PCA.

Of note, the robustness of our classification models is supported by the results of the sensitivity analyses using imputed data (e-Table 3, e-Table 4). Supplemental e-Table 4 indicates that the main characteristics between subjects with and without missing data did not differ substantially, minimizing selection bias.

For peer review only

Discussion

In our analysis of 1034 Swedish subjects with bicuspid and tricuspid aortic valves with and without prelevant aortopathy but devoid of coronary artery disease and primarily not planned for another concomitant valve surgery, we report two key findings. Firstly, prediction of aortic dilatation using automated machine learning methods and traditional regression models on multidimensional clinical data was possible only among TAV individuals, and *not* in patients with BAV. This suggests that general clinical cardiovascular risk profiles play more important

For peer review only - http://bmjopen.bmj.com/site/about/guidelines.xhtml

roles during aortic dilation in TAV patients than in patients with a BAV, and further supports that aortopathy associated with BAV and TAV, respectively, are clearly distinct with different underlying etiologies. Secondly, our study shows that the classification results were consistent for all machine learning algorithms and classic logistic regression models. This suggest that machine learning approaches might not outperform classical prediction methods in addressing complex interactions and non-linear relations between variables.

The strongest predictor for ascending aortic dilatation in TAV was the absence of aortic stenosis. This is in accordance with our previous observations that surgical patients with AS and ascending aortic dilatation almost exclusively have a BAV[18]. Whether this imply that biological processes associated with the development of AS in TAV also contributes to ascending aortic stability needs to be further elucidated. AS is commonly caused by progressive calcification of the aortic valve and increase in prevalence with age. In our cohort, TAV patients with dilated ascending aortas were significantly younger than TAV patients with non-dilated ascending aortas, which may be one contributing factor to the observed association and possibly reflects the surgical nature of the cohort. However, omitting patients with AS from the analysis still resulted in significant predictive values, although with worse discrimination, indicating that age alone cannot explain the association. Other strong contributors to the prediction model were diabetes and hsCRP. A negative association between diabetes and aneurysm in the abdominal aorta is well documented.[19] It has also been suggested that metformin prescription may associate with decreased risk of aortic dilatation and that the molecular mechanism involves a metformin-induced reduction in aortic inflammation.[20,21] Similarly, an elevated hsCRP has been described in AAA patients and found to be an independent risk factor for AAA.[22,23] Moreover, we have previously shown an inflammatory gene expression profile in dilated aortic tissue from TAV but not BAV patients.[7] In the present study, there was a

For peer review only

borderline significance of reduced hsCRP levels in BAV patients with dilated aorta. This together implies that aortic dilatation in TAV, in these aspects, may be more similar to aneurysm of the abdominal aorta than BAV aortic dilatation.

The lack of a good model for risk prediction of BAV-associated aortopathy raises the question, which other contributing factors may be of importance for aneurysm formation and development in these individuals? Two main hypotheses have been put forth in the literature.

Firstly, an altered flow in the proximal part of the ascending aorta due to the valve malformation itself has been suggested to provoke aortic dilatation. Also, different BAV morphotypes, i.e. cusp fusion pattern, have been shown to cause flow disturbances that affect the aorta in morphotype-dependent ways. [24] In our study, flow characteristics were not included in the predition model, which possibly could have influenced the results. However, we could not see any difference in the precense of aortic dilatation between different morphotypes, i.e. true BAV, LN-, RN- or RL cusp fucion (Table 1). Of note, other factors may also influence aortic flow patterns, e.g. eccentricity of valve opening due to valve disease, or vessel stiffnes. Indeed, it has been suggested that AS significantly alters aortic hemodynamic and wall shear stress, independent of aortic valve phenotype.

Secondly, the genetic contribution may override the influence of traditional risk factors to aneurysm development in patients with BAV. Although specific gene(s) and/or mutation(s) underlying BAV and BAV-associated aortopathy are still to be unravelled, several genes have been shown to be associated with both BAV formation and concomitant aortopathy in mice and humans.[25,26,27(p2)] Moreover, a high heratibility of BAV and/or other cardiovascular malformations have been demonstrated using segregation patterns in families, with a

For peer review only - http://bmjopen.bmj.com/site/about/guidelines.xhtml

heritability of BAV and BAV together with other cardiovascular malformations being as high as 89% and 75%, respectively.[28] A third, most likely, possibility is that both genetic factors and abnormal hemodynamic burden play central roles in BAV-associated aortopathy, interacting with each other and therby contribute to aortic dilatation.

Further dissecting differences between patients with non-dilated and dilated ascending aortas we found that, among others, PP, AS, AR and diabetes were associated with dilatation in BAV. PP is a well known risk factor for cardiovascular disease and the clinical manifestation of increased vascular stiffness.[29] Surprisingly, PP was higher in BAV patients with a non-dilated aorta, which may seem counterintuative since AR is associated with both increased PP and aortic dilatation. However, it may be speculated that the higher PP seen in these patients may rely on structural changes due to an increased hemodynamic burden associated with a BAV. In line with this, we have previously showed that BAV patients have a qualitative collagen defect in their ascending aorta, signified by a different collagen glycation compared with TAV patients and suggestive of an altered non-enzymatic collagen crosslinking.[30] Interestingly, we have also shown that dilated ascending aorta of BAV patients display an increased collagen-related stiffness compared with TAV patients.[31] Furthermore, The Strong Heart Study could also show that in patients free of prevalent coronary heart disease, aortic root dilatation was, at a given diastolic blood pressure and stroke volume, associated with lower pulse pressure.[32]

Additionally, accelerated vascular aging and increased arterial stiffness has previously been described in diabetic patients, the proportion of which was higher among BAV patients with non-dilated aortas.[33] However, we and others have previously demonstrated an increased vascular inflammation in dilated aorta in TAV- but not BAV patients, suggesting that other mechanisms could be involved in the protective role of diabetes on aneurysm formation in BAV

For peer review only - http://bmjopen.bmj.com/site/about/guidelines.xhtml

patients.[7,34] The association between dilatation and valve disease was not as pronounced in BAV- as in TAV patients, although AS was also negatively associated with dilatation in BAV, as previously found.[35] It may be speculated that the presence of AS increases flow velocities and blood pressure in the ascending aorta, thereby stimulating vascular remodeling and strengthening of the aortic wall. Whether this hamper the process of dilatation remains to be answered. The relation between degree of stenosis and width of the ascending aorta is complex, and a previous study found mid-ascending dilatation proportional to valve gradient when patients with small aortas were excluded.[35]

Our findings raise the issue of how to identify and implement prevention of aortopathy in BAV patients in a clinical setting. So far, clinicians have focused on aortic valve function and aortic dimensions to indicate cardiac surgery and recommend annual follow-up in asymptomatic patients to screen for associated aortopathy.[36] High importance has been given to the morphology of the valve, although in our study, dilatation in BAV did not show any significant association with valve morphology. Of note, previous studies establishing an association between BAV cusp fusion and clinical outcomes relied on small sample size based on imaging diagnostic rather than anatomic diagnosis.[37]

Study strengths and limitations

In this study, comprehensive clinical data, including blood sampling as well as epidemiological data was used in the analysis of in total 1034 individuals (543 BAV and 491 TAV). The morphology of aortic valves was evaluated by visual inspection during open-heart surgery, which is a major strength compared to only echocardiography in terms of reliability.

For peer review only

A few limitations must however be highlighted. Firstly, by design, only individuals devoid of significant coronary artery disease were included, which may introduce a selection bias and an overestimation of the prevalence of subjects with aneurysm. Secondly, as this is a population based surgical cohort, it is possible that our study population included BAV and TAV patients with worse outcomes compared to their counterparts of similar age. This should however not affect the associations between valve type and aortic dilatation. Lastly, TAV patients with non-dilated aortas were significantly older than TAV indivuduals with a dilated aorta, which could possibly explain the higher degree of patients with aortic stenosis in this group.

To conclude, using automated machine learning algorithms and classic logistic regression models we demonstrated that in TAV patients, cardiovascular risk profiles appear to be more predictive of aortopathy than in BAV patients. The good performance of the TAV classifier also after exclusion of AS offers important implications for better targeting TAV individuals that are of a high risk of developing aneurysm. The lack of good models to develop clinical classifiers of BAV-associated aortopathy strengthen the focus of genetics and/or flow as important contributing factors to aneurysm development in these individuals.

For peer review only

Contributorship statement

Author Contributions: Gaye, Franco-Cereceda, Eriksson, and Björck had full access to all the data in the study and take responsibility for the integrity of the data and the accuracy of the data analysis.

Study concept and design: Gaye, Eriksson

Acquisition, analysis, or interpretation of data: Gaye, Vignac, Gådin, Ladouceur, Caidahl, Olsson, Franco-Cereceda, Eriksson, Björck

Drafting of the manuscript: Gaye

Critical revision of the manuscript for important intellectual content: Gaye, Vignac, Gådin, Ladouceur, Caidahl, Olsson, Franco-Cereceda, Eriksson, Björck

Statistical analysis: Gaye and Vignac

Study supervision: Gaye, Eriksson

Competing interests

The authors declare no competing interests

Funding

The work was supported by the Swedish Research Council [12660]; the Swedish Heart-Lung Foundation [20180451, 20170669]; the Stockholm County Council [20180072]; La Marató de TV3 [20151330]; and a donation by Fredrik Lundberg.

Data sharing statement

The clinical trial data of this article are available upon reasonable request to the corresponding author.

For peer review only

Ethics Approval Statement

The study was approved by the Regional Ethics Review Board (application number 2006/784-31/1; 2012/1633-31/4), Stockholm, Sweden. Oral and written informed consent were obtained from all patients according to the declaration of Helsinki.

For peer review only

References

1. Jackson V, Petrini J, Caidahl K, Eriksson MJ, Liska J, Eriksson P, Franco-Cereceda A. Bicuspid aortic valve leaflet morphology in relation to aortic root morphology: a study of 300 patients undergoing open-heart surgery. *Eur J Cardiothorac Surg*. Published online May 2011:S1010794011004623. doi:10.1016/j.ejcts.2011.04.014
2. Messner B, Bernhard D. Bicuspid aortic valve-associated aortopathy: Where do we stand? *J Mol Cell Cardiol*. 2019;133:76-85. doi:10.1016/j.yjmcc.2019.05.023
3. Ward C. Clinical significance of the bicuspid aortic valve. *Heart*. 2000;83(1):81-85. doi:10.1136/heart.83.1.81
4. Williams DS. Bicuspid aortic valve. *J Insur Med N Y N*. 2006;38(1):72-74.
5. Losenno KL, Goodman RL, Chu MWA. Bicuspid Aortic Valve Disease and Ascending Aortic Aneurysms: Gaps in Knowledge. *Cardiol Res Pract*. 2012;2012:1-16. doi:10.1155/2012/145202
6. Verma S, Siu SC. Aortic dilatation in patients with bicuspid aortic valve. *N Engl J Med*. 2014;370(20):1920-1929. doi:10.1056/NEJMra1207059
7. Folkersen L, Wågsäter D, Paloschi V, Jackson V, Petrini J, Kurtovic S, Maleki S, Eriksson MJ, Caidahl K, Hamsten A, Michel J-B, Liska J, Gabrielsen A, Franco-Cereceda A, Eriksson P. Unraveling Divergent Gene Expression Profiles in Bicuspid and Tricuspid Aortic Valve Patients with Thoracic Aortic Dilatation: The ASAP Study. *Mol Med*. 2011;17(11-12):1365-1373. doi:10.2119/molmed.2011.00286
8. Lang RM, Bierig M, Devereux RB, Flachskampf FA, Foster E, Pellikka PA, Picard MH, Roman MJ, Seward J, Shanewise JS, Solomon SD, Spencer KT, St John Sutton M, Stewart WJ. Recommendations for Chamber Quantification: A Report from the American Society of Echocardiography's Guidelines and Standards Committee and the Chamber Quantification Writing Group, Developed in Conjunction with the European Association of Echocardiography, a Branch of the European Society of Cardiology. *J Am Soc Echocardiogr*. 2005;18(12):1440-1463. doi:10.1016/j.echo.2005.10.005
9. Zoghbi W. Recommendations for evaluation of the severity of native valvular regurgitation with two-dimensional and doppler echocardiography. *J Am Soc Echocardiogr*. 2003;16(7):777-802. doi:10.1016/S0894-7317(03)00335-3
10. Baumgartner H, Hung J, Bermejo J, Chambers JB, Evangelista A, Griffin BP, Iung B, Otto CM, Pellikka PA, Quiñones M. Echocardiographic Assessment of Valve Stenosis: EAE/ASE Recommendations for Clinical Practice. *J Am Soc Echocardiogr*. 2009;22(1):1-23. doi:10.1016/j.echo.2008.11.029
11. Walter S, Tiemeier H. Variable selection: current practice in epidemiological studies. *Eur J Epidemiol*. 2009;24(12):733-736. doi:10.1007/s10654-009-9411-2
12. Tibshirani R. The lasso method for variable selection in the Cox model. *Stat Med*. 1997;16(4):385-395. doi:10.1002/(sici)1097-0258(19970228)16:4<385::aid-sim380>3.0.co;2-3
13. Nishimura Rick A., Otto Catherine M., Bonow Robert O., Carabello Blase A., Erwin John P., Guyton Robert A., O'Gara Patrick T., Ruiz Carlos E., Skubas Nikolaos J., Sorajja Paul, Sundt Thoralf M., Thomas James D. 2014 AHA/ACC Guideline for the Management of Patients With Valvular Heart Disease: Executive Summary. *Circulation*. 2014;129(23):2440-2492. doi:10.1161/CIR.0000000000000029
14. Sievers H-H, Schmidtke C. A classification system for the bicuspid aortic valve from 304 surgical specimens. *J Thorac Cardiovasc Surg*. 2007;133(5):1226-1233. doi:10.1016/j.jtcvs.2007.01.039
15. Singal AG, Mukherjee A, Elmunzer JB, Higgins PDR, Lok AS, Zhu J, Marrero JA, Waljee AK. Machine Learning Algorithms Outperform Conventional Regression Models in Predicting Development of Hepatocellular Carcinoma: *Am J Gastroenterol*.

For peer review only - http://bmjopen.bmj.com/site/about/guidelines.xhtml

2013;108(11):1723-1730. doi:10.1038/ajg.2013.332
16. Lambin P, van Stiphout RGPM, Starmans MHW, Rios-Velazquez E, Nalbantov G, Aerts HJWL, Roelofs E, van Elmpt W, Boutros PC, Granone P, Valentini V, Begg AC, De Ruysscher D, Dekker A. Predicting outcomes in radiation oncology—multifactorial decision support systems. *Nat Rev Clin Oncol*. 2013;10(1):27-40. doi:10.1038/nrclinonc.2012.196
17. Fern'andez-Delgado M, Cernadas E, Barro S, Amorim D. Do we need hundreds of classifiers to solve real world classification problems? *J Mach Learn Res*. 2014;15(1).
18. Jackson V, Petrini J, Eriksson MJ, Caidahl K, Eriksson P, Franco-Cereceda A. Aortic dimensions in relation to bicuspid and tricuspid aortic valve pathology. *J Heart Valve Dis*. 2014;23(4):463-472.
19. Takagi H, Umemoto T, for the ALICE (All-Literature Investigation of Cardiovascular Evidence) Group. Negative association of diabetes with rupture of abdominal aortic aneurysm. *Diab Vasc Dis Res*. 2016;13(5):341-347. doi:10.1177/1479164116651389
20. Yu X, Jiang D, Wang J, Wang R, Chen T, Wang K, Durgahee MSA, Wei X, Cao S. Metformin prescription and aortic aneurysm: systematic review and meta-analysis. *Heart*. 2019;105(17):1351-1357. doi:10.1136/heartjnl-2018-314639
21. Li L, Mamputu J-C, Wiernsperger N, Renier G. Signaling Pathways Involved in Human Vascular Smooth Muscle Cell Proliferation and Matrix Metalloproteinase-2 Expression Induced by Leptin: Inhibitory Effect of Metformin. *Diabetes*. 2005;54(7):2227-2234. doi:10.2337/diabetes.54.7.2227
22. Vainas T, Lubbers T, Stassen FRM, Herngreen SB, van Dieijen-Visser MP, Bruggeman CA, Kitslaar PJEHM, Schurink GWH. Serum C-Reactive Protein Level Is Associated With Abdominal Aortic Aneurysm Size and May Be Produced by Aneurysmal Tissue. *Circulation*. 2003;107(8):1103-1105. doi:10.1161/01.CIR.0000059938.95404.92
23. Shangwei Z, Yingqi W, Jiang X, Zhongyin W, Juan J, Dafang C, Yonghua H, Wei G. Serum High-Sensitive C-Reactive Protein Level and CRP Genetic Polymorphisms Are Associated with Abdominal Aortic Aneurysm. *Ann Vasc Surg*. 2017;45:186-192. doi:10.1016/j.avsg.2017.05.024
24. McNally A, Madan A, Sucosky P. Morphotype-Dependent Flow Characteristics in Bicuspid Aortic Valve Ascending Aortas: A Benchtop Particle Image Velocimetry Study. *Front Physiol*. 2017;8. doi:10.3389/fphys.2017.00044
25. Yang B, Zhou W, Jiao J, Nielsen JB, Mathis MR, Heydarpour M, Lettre G, Folkersen L, Prakash S, Schurmann C, Fritsche L, Farnum GA, Lin M, Othman M, Hornsby W, Driscoll A, Levasseur A, Thomas M, Farhat L, Dubé M-P, Isselbacher EM, Franco-Cereceda A, Guo D, Bottinger EP, Deeb GM, Booher A, Kheterpal S, Chen YE, Kang HM, Kitzman J, Cordell HJ, Keavney BD, Goodship JA, Ganesh SK, Abecasis G, Eagle KA, Boyle AP, Loos RJF, Eriksson P, Tardif J-C, Brummett CM, Milewicz DM, Body SC, Willer CJ. Protein-altering and regulatory genetic variants near GATA4 implicated in bicuspid aortic valve. *Nat Commun*. 2017;8(1):15481. doi:10.1038/ncomms15481
26. Baylor-Hopkins Center for Mendelian Genomics, MIBAVA Leducq Consortium, Gould RA, Aziz H, Woods CE, Seman-Senderos MA, Sparks E, Preuss C, Wünnemann F, Bedja D, Moats CR, McClymont SA, Rose R, Sobreira N, Ling H, MacCarrick G, Kumar AA, Luyckx I, Cannaerts E, Verstraeten A, Björk HM, Lehsau A-C, Jaskula-Ranga V, Lauridsen H, Shah AA, Bennett CL, Ellinor PT, Lin H, Isselbacher EM, Lino Cardenas CL, Butcher JT, Hughes GC, Lindsay ME, Mertens L, Franco-Cereceda A, Verhagen JMA, Wessels M, Mohamed SA, Eriksson P, Mital S, Van Laer L, Loeys BL, Andelfinger G, McCallion AS, Dietz HC. ROBO4 variants predispose individuals to bicuspid aortic valve and thoracic aortic aneurysm. *Nat Genet*. 2019;51(1):42-50. doi:10.1038/s41588-018-0265-y
27. Pasipoularides A. Clinical-pathological correlations of BAV and the attendant thoracic aortopathies. Part 2: Pluridisciplinary perspective on their genetic and molecular origins. *J*

For peer review only - http://bmjopen.bmj.com/site/about/guidelines.xhtml

Mol Cell Cardiol. 2019;133:233-246. doi:10.1016/j.yjmcc.2019.05.022
28. Cripe L, Andelfinger G, Martin LJ, Shooner K, Benson DW. Bicuspid aortic valve is heritable. *J Am Coll Cardiol*. 2004;44(1):138-143. doi:10.1016/j.jacc.2004.03.050
29. Georgakis MK, Gill D, Malik R, Protogerou AD, Webb AJS, Dichgans M. Genetically Predicted Blood Pressure Across the Lifespan: Differential Effects of Mean and Pulse Pressure on Stroke Risk. *Hypertension*. 2020;76(3):953-961. doi:10.1161/HYPERTENSIONAHA.120.15136
30. Wågsäter D, Paloschi V, Hanemaaijer R, Hultenby K, Bank RA, Franco-Cereceda A, Lindeman JHN, Eriksson P. Impaired Collagen Biosynthesis and Cross-linking in Aorta of Patients With Bicuspid Aortic Valve. *J Am Heart Assoc*. 2013;2(1). doi:10.1161/JAHA.112.000034
31. Forsell C, Björck HM, Eriksson P, Franco-Cereceda A, Gasser TC. Biomechanical Properties of the Thoracic Aneurysmal Wall: Differences Between Bicuspid Aortic Valve and Tricuspid Aortic Valve Patients. *Ann Thorac Surg*. 2014;98(1):65-71. doi:10.1016/j.athoracsur.2014.04.042
32. de Simone G, Roman MJ, De Marco M, Bella JN, Izzo R, Lee ET, Devereux RB. Hemodynamic Correlates of Abnormal Aortic Root Dimension in an Adult Population: The Strong Heart Study. *J Am Heart Assoc*. 2015;4(10). doi:10.1161/JAHA.115.002309
33. Schram MT, Kostense PJ, van Dijk RAJM, Dekker JM, Nijpels G, Bouter LM, Heine RJ, Stehouwer CDA. Diabetes, pulse pressure and cardiovascular mortality: the Hoorn Study: *J Hypertens*. 2002;20(9):1743-1751. doi:10.1097/00004872-200209000-00017
34. Grewal N, Gittenberger-de Groot AC, Poelmann RE, Klautz RJM, Lindeman JHN, Goumans M-J, Palmen M, Mohamed SA, Sievers H-H, Bogers AJJC, DeRuiter MC. Ascending aorta dilation in association with bicuspid aortic valve: A maturation defect of the aortic wall. *J Thorac Cardiovasc Surg*. 2014;148(4):1583-1590. doi:10.1016/j.jtcvs.2014.01.027
35. Della Corte A, Bancone C, Quarto C, Dialetto G, Covino FE, Scardone M, Caianiello G, Cotrufo M. Predictors of ascending aortic dilatation with bicuspid aortic valve: a wide spectrum of disease expression☆. *Eur J Cardiothorac Surg*. 2007;31(3):397-405. doi:10.1016/j.ejcts.2006.12.006
36. Baumgartner H, Falk V, Bax JJ, De Bonis M, Hamm C, Holm PJ, Iung B, Lancellotti P, Lansac E, Rodriguez Muñoz D, Rosenhek R, Sjögren J, Tornos Mas P, Vahanian A, Walther T, Wendler O, Windecker S, Zamorano JL, ESC Scientific Document Group, Roffi M, Alfieri O, Agewall S, Ahlsson A, Barbato E, Bueno H, Collet J-P, Coman IM, Czerny M, Delgado V, Fitzsimons D, Folliguet T, Gaemperli O, Habib G, Harringer W, Haude M, Hindricks G, Katus HA, Knuuti J, Kolh P, Leclercq C, McDonagh TA, Piepoli MF, Pierard LA, Ponikowski P, Rosano GMC, Ruschitzka F, Shlyakhto E, Simpson IA, Sousa-Uva M, Stepinska J, Tarantini G, Tchétché D, Aboyans V, Windecker S, Aboyans V, Agewall S, Barbato E, Bueno H, Coca A, Collet J-P, Coman IM, Dean V, Delgado V, Fitzsimons D, Gaemperli O, Hindricks G, Iung B, Jüni P, Katus HA, Knuuti J, Lancellotti P, Leclercq C, McDonagh T, Piepoli MF, Ponikowski P, Richter DJ, Roffi M, Shlyakhto E, Simpson IA, Zamorano JL, Kzhdryan HK, Mascherbauer J, Samadov F, Shumavets V, Camp GV, Lončar D, Lovric D, Georgiou GM, Linhartova K, Ihlemann N, Abdelhamid M, Pern T, Turpeinen A, Srbinovska-Kostovska E, Cohen A, Bakhutashvili Z, Ince H, Vavuranakis M, et al. 2017 ESC/EACTS Guidelines for the management of valvular heart disease. *Eur Heart J*. 2017;38(36):2739-2791. doi:10.1093/eurheartj/ehx391
37. Schaefer BM, Lewin MB, Stout KK, Gill E, Prueitt A, Byers PH, Otto CM. The bicuspid aortic valve: an integrated phenotypic classification of leaflet morphology and aortic root shape. *Heart*. 2008;94(12):1634-1638. doi:10.1136/hrt.2007.132092

For peer review only - http://bmjopen.bmj.com/site/about/guidelines.xhtml

Figure 1. Principal component analysis (PCA) plot of BAV versus TAV patients (with or without aneurysm).

A, BAV patients

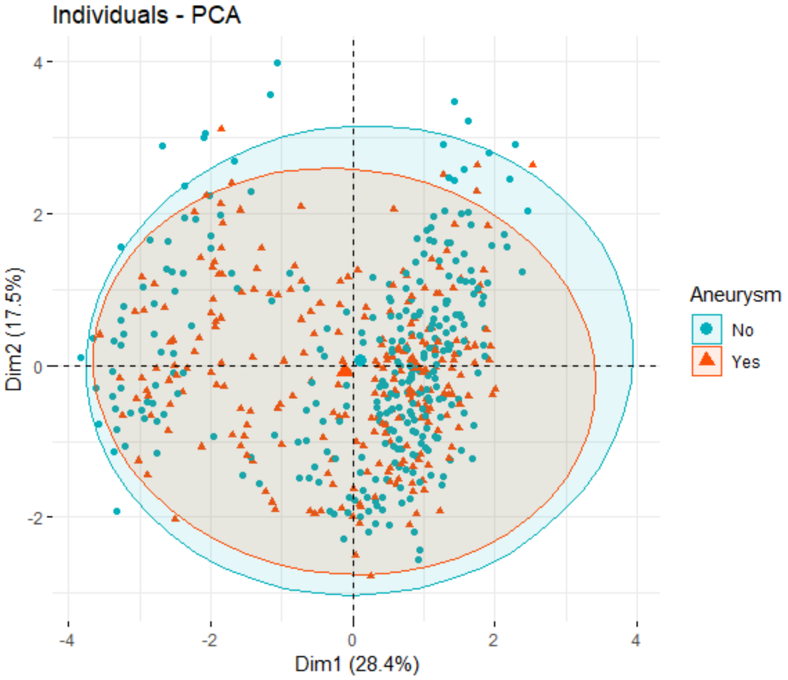

B, TAV patients

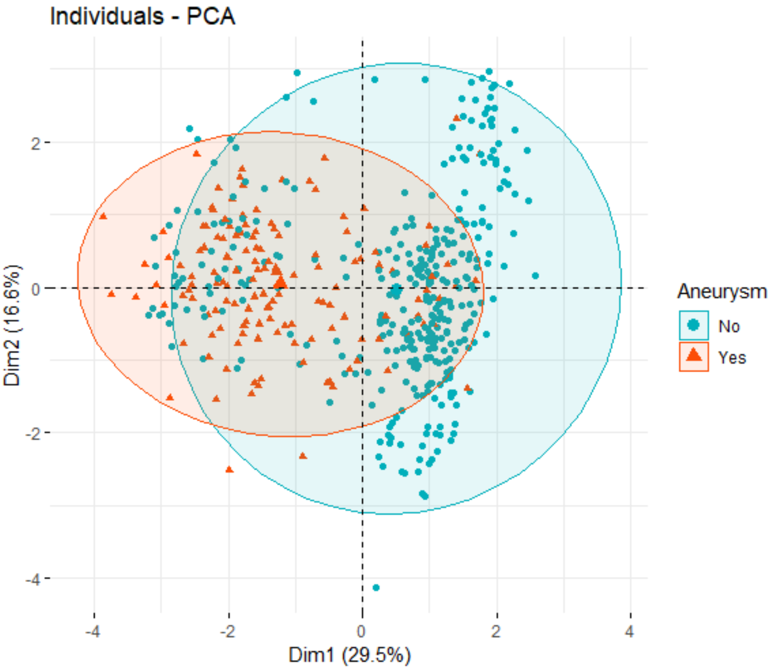

Figure 2. The ROC curve for risk prediction model across the predictive methods used.

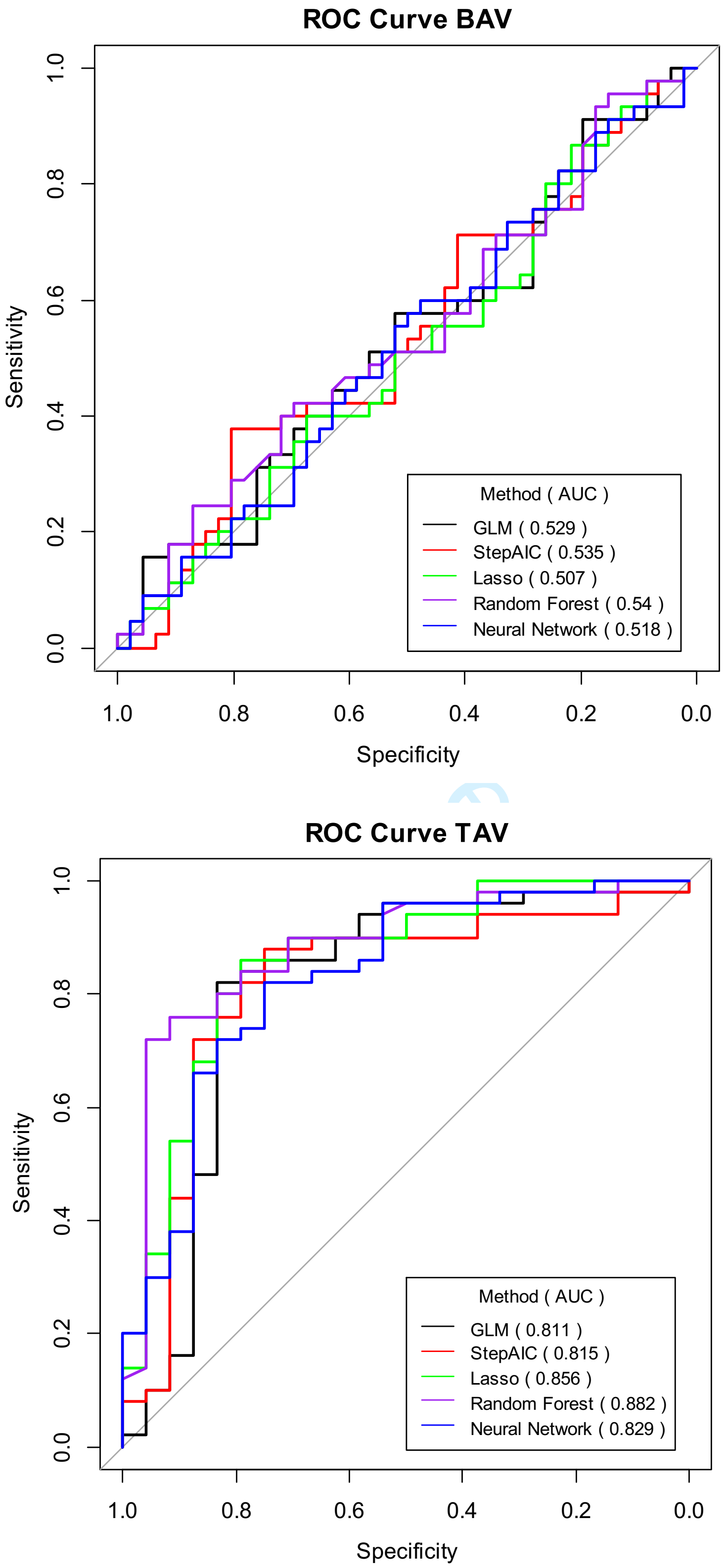

Clinical classifiers to identify ascending aortic dilatation in patients with bicuspid versus tricuspid aortic valves

Supplementary Appendix Table of Contents:

Tables

e-Table 1: Characteristics of Patients with BAV vs. TAV
e-Table 2. Performance of the algorithms
e-Table 3: Predictors of ascending aortic dilatation in BAV and TAV patients, separately. Univariate and Multivariate logistic regression analysis: Imputed data
e-Table 4: Baseline characteristics of included and excluded study participants.

e-Table 5: Contributors to the prediction of aortic dilatation in TAV

Figures

e-Figure 1: Flowchart of the study
e-Figure 2: Plot of methodology used to choose variables
e-Figure 3: The ROC curve for our risk prediction model across the predictive methods using imputed data
e-Figure 4. Principal component analysis (PCA) plot of TAV patients with or without aneurysm stratified by AS
e-Figure 5. The ROC curve for our risk prediction model across the predictive methods without AS as predictor

Dataset selection:

In order to gather maximum of information from the available measurement in the ASAP/DAVAACA cohort, we first created several blocks of meaningful variables: clinical data, family history of cardiovascular diseases, prevalent diseases, demographic characteristics, lifestyle habits data, blood sample data and medication. Then, to select the most meaningful variables by avoiding collinearity within each bloc, we performed several analyses: (1) a hierarchical clustering, then (2) we ran Principal Component Analysis, (3) we lastly created the correlation matrix inside each cluster (and its correlation plot) (**Figure e-2**).

For peer review only

e-Table 1: Characteristics of Patients with BAV vs. TAV

	BAV N=543	TAV N=491	P value
**Gender: Male**	397 (73.10%)	311 (63.30%)	0.001
**Age**	60.4 (12.40)	70.4 (9.09)	<0.001
**Height**	175 (9.30)	172 (9.80)	<0.001
**Weight**	81.9 (15.80)	81.5 (15.90)	0.697
**BSA**	1.98 (0.22)	1.94 (0.22)	0.012
**BMI**	26.6 (4.26)	27.4 (4.77)	0.002
**Regular Smoker:**			0.023
**No**	250 (46.6%)	213 (43.6%)	
**Former**	234 (43.7%)	246 (50.4%)	
**Yes**	52 (9.70%)	29 (5.94%)	
**Systolic Blood Pressure**	136 (18.6)	142 (20.4)	<0.001
**Diastolic Blood Pressure**	80.1 (11.9)	76.1 (13.2)	<0.001
**PP**	55.4 (17.6)	66.4 (19.7)	<0.001
**Leukocytes**	5.80 [4.80;6.90]	6.00 [5.03;7.40]	0.004
**hsCRP**	1.10 [0.55;2.50]	1.80 [0.79;3.80]	<0.001
**Cholesterol**	4.80 [4.20;5.60]	4.60 [3.90;5.60]	0.016
**LDL**	2.80 [2.20;3.60]	2.60 [2.00;3.50]	0.009
**Aortic Stenosis:**	404 (74.4%)	306 (62.3%)	<0.001
**Aortic Insufficiency:**	158 (29.1%)	160 (32.6%)	0.252
**Diameter of Aortic Anulus**	24.8 (3.57)	23.0 (3.08)	<0.001
**Diameter of Valsalva Sinus**	35.6 (5.68)	34.9 (6.94)	0.056
**Diameter of Sinotubular Junction**	30.5 (5.70)	29.0 (6.49)	<0.001
**Diameter of Ascending Aorta**	39.3 (8.29)	37.1 (10.1)	<0.001
**Dilatation of Ascending Aorta:**	297 (54.7%)	349 (71.1%)	<0.001
**Sibling MI history before 65 year**	32 (6.24%)	46 (10.0%)	0.041
**Mother MI history before 65 year**	25 (4.84%)	32 (6.97%)	0.199
**Father MI history before 65 year**	69 (13.7%)	63 (14.3%)	0.874
**Myocardial Infarction**	19 (3.53%)	42 (8.59%)	0.001
**Stroke**	30 (5.55%)	57 (11.7%)	0.001
**Abdominal Aorta Aneurysm**	25 (4.63%)	30 (6.19%)	0.335
**Angina Pectoris**	35 (6.49%)	61 (12.5%)	0.001
**Heart Failure**	40 (7.50%)	57 (11.7%)	0.029
**Hypertension**	255 (47.4%)	311 (64.4%)	<0.001
**Diabetes**	54 (10.0%)	64 (13.1%)	0.139
**ACE inhibitor**	103 (19.0%)	142 (29.0%)	<0.001
**Aspirin**	132 (24.4%)	193 (39.6%)	<0.001
**Betablocker**	217 (40.1%)	255 (52.0%)	<0.001

For peer review only

e-Table 2. Performance of the algorithms

BAV						
**Method**	**Accuracy**	**AUC**	**Sensitivity**	**Specificity**	**Positive Predictive Value**	**Negative Predictive Value**
**GLM**	0.528	0.529	0.444	0.609	0.526	0.528
**StepAIC**	0.506	0.535	0.422	0.587	0.500	0.509
**Lasso**	0.484	0.507	0.422	0.544	0.475	0.490
**Random Forest**	0.517	0.540	0.467	0.565	0.512	0.520
**Neural Network**	0.473	0.518	0.489	0.457	0.468	0.477
TAV						
**Method**	**Accuracy**	**AUC**	**Sensitivity**	**Specificity**	**Positive Predictive Value**	**Negative Predictive Value**
**GLM**	0.824	0.811	0.820	0.833	0.911	0.690
**StepAIC**	0.824	0.815	0.900	0.667	0.849	0.762
**Lasso**	0.824	0.854	0.860	0.750	0.878	0.720
**Random Forest**	0.797	0.882	0.840	0.708	0.857	0.680
**Neural Network**	0.770	0.825	0.820	0.667	0.837	0.640
BAV (Imputed data)						
**Method**	**Accuracy**	**AUC**	**Sensitivity**	**Specificity**	**Positive Predictive Value**	**Negative Predictive Value**
**GLM**	0.570	0.586	0.689	0.426	0.593	0.531
**StepAIC**	0.563	0.526	0.676	0.426	0.588	0.520
**Lasso**	0.578	0.593	0.676	0.459	0.602	0.539
**Random Forest**	0.556	0.556	0.662	0.426	0.583	0.548
**Neural Network**	0.541	0.540	0.581	0.492	0.581	0.492
TAV (Imputed data)						
**Method**	**Accuracy**	**AUC**	**Sensitivity**	**Specificity**	**Positive Predictive Value**	**Negative Predictive Value**
**GLM**	0.820	0.831	0.816	0.829	0.922	0.644
**StepAIC**	0.803	0.838	0.816	0.771	0.899	0.628
**Lasso**	0.812	0.842	0.793	0.857	0.932	0.625
**Random Forest**	0.820	0.857	0.828	0.800	0.911	0.651
**Neural Network**	0.812	0.799	0.862	0.686	0.872	0.667

For peer review only

e-Table 3. Predictors of ascending aortic dilatation in BAV and TAV patients, separately. Univariate and Multivariate logistic regression analysis: Imputed data

	**BAV imputed**						**TAV imputed**					
	unAdjusted Model		***Model 1***		***Model 2***		***unAdjusted Model***		***Model 1***		***Model 2***	
	OR (95% IC)*	***p***	***OR (95% IC)****	***p***	***OR (95% IC)****	***p***	***OR (95% IC)****	***p***	***OR (95% IC)****	***p***	***OR (95% IC)****	***p***
**Aortic Stenosis**	0.58 (0.39-0.85)	0.006	0.43 (0.27-0.67)	0.002	0.42 (0.22-0.79)	0.007	0.05 (0.03-0.07)	<0.001	0.04 (0.02-0.07)	<0.001	0.02 (0.01-0.05)	<0.001
**Aortic Regurgitation**	1.51 (1.04-2.19)	0.031	1.89 (1.24-2.88)	0.003	1.35 (0.75-2.44)	0.316	7.99 (5.19-12.45)	<0.001	7.27 (4.56-11.77)	<0.001	0.51 (0.20-1.18)	0.133
**Pulse Pressure**	0.99 (0.98-1.00)	0.009	0.99 (0.98-1.00)	0.013	0.98 (0.97-0.99)	<0.001	1.01 (1.00-1.02)	0.088	1.01 (1.00-1.02)	0.163	0.99 (0.98-1.00)	0.132
**Diabetes**	0.42 (0.22-0.76)	0.006	0.34 (0.18-0.65)	0.001	0.39 (0.19-0.74)	0.005	0.10 (0.02-028)	<0.001	0.10 (0.02-0.29)	<0.001	0.21 (0.05-0.71)	0.022

* Odds ratio and 95% confidence interval limits were obtained by logistic regression and were adjusted for age, sex, body surface area (BSA), low-density Lipoprotein (LDL), high-sensitivity C-reactive protein (hsCRP) and Diabetes

For peer review only

e-Table 4. Baseline characteristics of included and excluded study participants.

	[ALL] N=1180	Data N=1034	NA N=139	p.overall	N
**Gender: Male**	807 (68.4%)	708 (68.5%)	99 (67.8%)	0.947	1180
**Age**	64.9 (12.4)	65.2 (12.0)	62.9 (14.5)	0.077	1180
**Height**	174 (9.60)	174 (9.65)	175 (9.15)	0.263	1171
**Weight**	81.8 (15.6)	81.7 (15.8)	82.6 (14.4)	0.514	1168
**BSA**	1.96 (0.21)	1.96 (0.22)	1.98 (0.19)	0.305	1141
**BMI**	27.0 (4.56)	27.0 (4.53)	27.1 (4.80)	0.746	1167
**Regular Smoker:**				0.549	1168
**No**	535 (45.8%)	463 (45.2%)	72 (50.0%)		
**Former**	541 (46.3%)	480 (46.9%)	61 (42.4%)		
**Yes**	92 (7.88%)	81 (7.91%)	11 (7.64%)		
**Cusps:**				0.951	1140
**BAV**	599 (52.5%)	543 (52.5%)	56 (52.8%)		
**TAV**	541 (47.5%)	491 (47.5%)	50 (47.2%)		
**Raphe:**				.	1158
**True BAV**	50 (4.32%)	47 (4.60%)	3 (2.19%)		
**Left-Non coronary**	6 (0.52%)	5 (0.49%)	1 (0.73%)		
**Right-Non coronary**	99 (8.55%)	90 (8.81%)	9 (6.57%)		
**Right-Left**	432 (37.3%)	388 (38.0%)	44 (32.1%)		
**Systolic Blood Pressure**	138 (19.7)	139 (19.8)	136 (18.8)	0.053	1172
**Diastolic Blood Pressure**	78.3 (12.7)	78.2 (12.7)	79.3 (12.7)	0.305	1171
**PP**	60.1 (19.2)	60.6 (19.4)	56.2 (17.2)	0.005	1171
**Leukocytes**	5.90 [4.90;7.10]	5.90 [4.90;7.10]	5.90 [4.90;6.90]	0.665	1168
**hsCRP**	1.40 [0.64;3.20]	1.40 [0.63;3.10]	1.60 [0.72;3.52]	0.153	1159
**Cholesterol**	4.80 [4.00;5.68]	4.80 [4.00;5.60]	5.10 [4.30;6.10]	0.004	1162
**LDL**	2.80 [2.20;3.60]	2.80 [2.10;3.50]	3.20 [2.30;3.90]	0.002	1146
**Aortic Stenosis:**	813 (69.1%)	710 (68.7%)	103 (72.0%)	0.472	1177
**Aortic Insufficiency:**	361 (30.7%)	318 (30.8%)	43 (30.1%)	0.944	1177
**Diameter of Aortic Anulus**	23.9 (3.40)	24.0 (3.46)	23.5 (2.87)	0.148	1154
**Diameter of Valsalva Sinus**	35.4 (6.27)	35.3 (6.32)	36.2 (5.83)	0.095	1157
**Diameter of Sinotubular Junction**	29.9 (6.05)	29.8 (6.13)	30.7 (5.34)	0.083	1112
**Diameter of Ascending Aorta**	38.3 (9.24)	38.2 (9.28)	38.8 (8.09)	0.686	1069
**Dilatation of Ascending Aorta:**	402 (37.6%)	388 (37.5%)	14 (40.0%)	0.904	1069
**Sibling MI history before 65 year**	86 (7.76%)	78 (8.02%)	8 (5.93%)	0.497	1108
**Mother MI history before 65 year**	63 (5.65%)	57 (5.84%)	6 (4.29%)	0.583	1116
**Father MI history before 65 year**	151 (14.0%)	132 (14.0%)	19 (14.2%)	0.959	1076
**Myocardial Infarction**	66 (5.64%)	61 (5.94%)	5 (3.47%)	0.313	1171
**Stroke**	99 (8.43%)	87 (8.45%)	12 (8.33%)	0.964	1174
**Abdominal Aorta Aneurysm**	58 (4.96%)	55 (5.37%)	3 (2.07%)	0.132	1170
**Angina Pectoris**	103 (8.80%)	96 (9.35%)	7 (4.86%)	0.105	1171
**Heart Failure**	112 (9.64%)	97 (9.52%)	15 (10.5%)	0.828	1162
**Hypertension**	635 (54.5%)	566 (55.4%)	69 (47.6%)	0.092	1166
**Diabetes**	136 (11.6%)	118 (11.5%)	18 (12.4%)	0.852	1172
**ACE inhibitor:**	277 (23.6%)	245 (23.8%)	32 (22.4%)	0.790	1173
**Aspirin:**	352 (30.1%)	325 (31.6%)	27 (18.9%)	0.003	1171

For peer review only

	[ALL] N=1180	Data N=1034	NA N=139	p.overall	N
**Betablocker:**	530 (45.1%)	472 (45.8%)	58 (40.6%)	0.277	1174

For peer review only

e-Table 5. Contributors to the prediction of aortic dilatation in TAV

**GLM**		**StepAIC (top 10)**		**Lasso (top 10)**		**RF (top 10)**		**Neural network (top 10)**	
**AS**	100.000	**AS**	100.000	**AS**	100.000	**AS**	100.000	**AS**	100.00
**age**	27.762	**AI**	70.043	**Diabetes**	39.6265	**AI**	23.581	**age**	62.81
**Diabetes**	27.003	**age**	41.852	**Renal Failure**	26.2556	**Creatinine**	21.571	**Diabetes**	47.28
**AI**	21.226	**LDL**	31.027	**Father Stroke o r Mi or TIA65**	16.4437	**age**	21.181	**Beta blocker**	41.58
**BSA**	17.970	**Hemoglobin**	27.641	**MI**	15.3655	**PP**	16.418	**Creatinine**	38.18
**Pulse BP**	16.595	**ASA**	26.802	**ALAT**	14.7816	**Leukocytes**	14.558	**FatherMI65**	35.95
**LDL**	8.753	**Cholesterol**	26.657	**Sibling AP65**	14.7506	**hsCRP**	13.549	**Diastolic BP**	34.47
**Gender**	7.564	**Diabetes**	26.541	**HF**	12.8772	**Systolic BP**	13.540	**Deep VTPEM**	33.27
**hsCRP**	0.000	**hsCRP**	22.648	**Father MI65**	12.4264	**Height**	13.199	**Father Stroke or Mi or TIA65**	33.16

For peer review only

e-Figure 1: Flowchart of the study

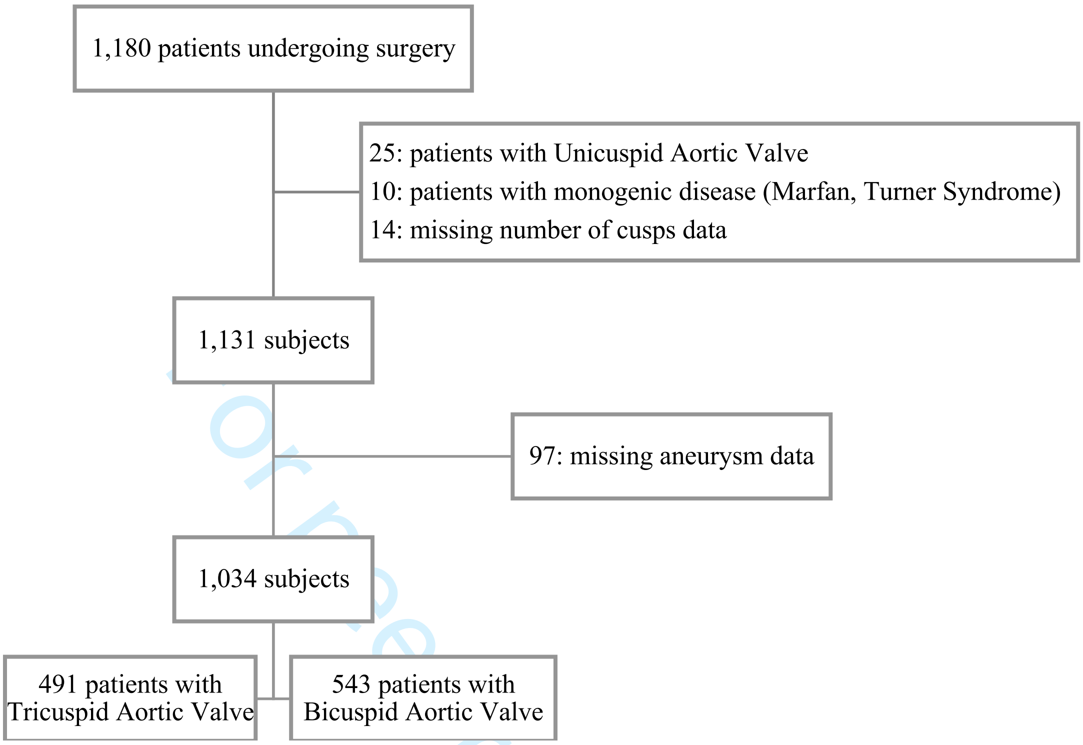

e-Figure 2. Plot of methodology used to choose variables

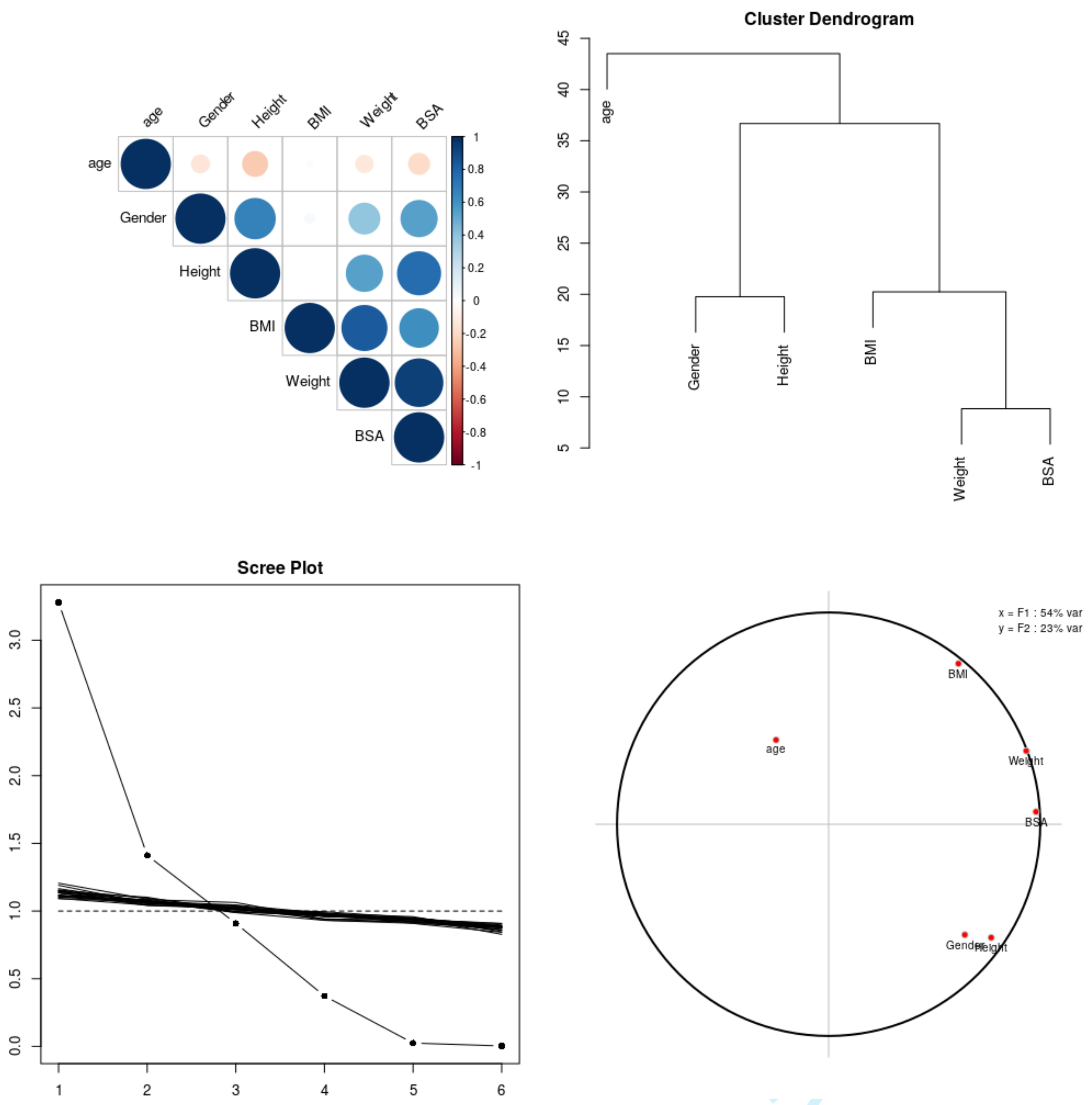

e-Figure 3. The ROC curve for our risk prediction model across the predictive methods using imputed data

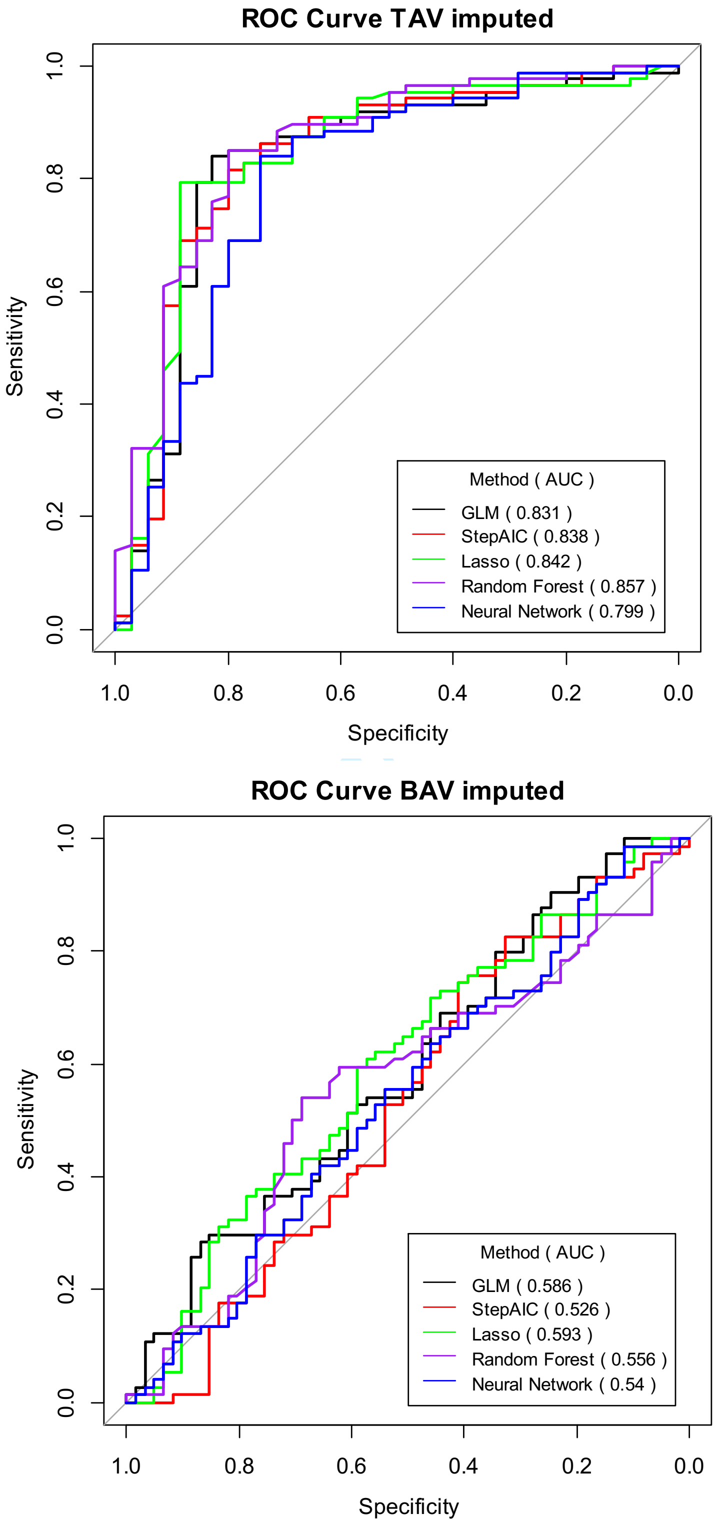

e-Figure 4. Principal component analysis (PCA) plot of TAV patients with or without aneurysm stratified by AS

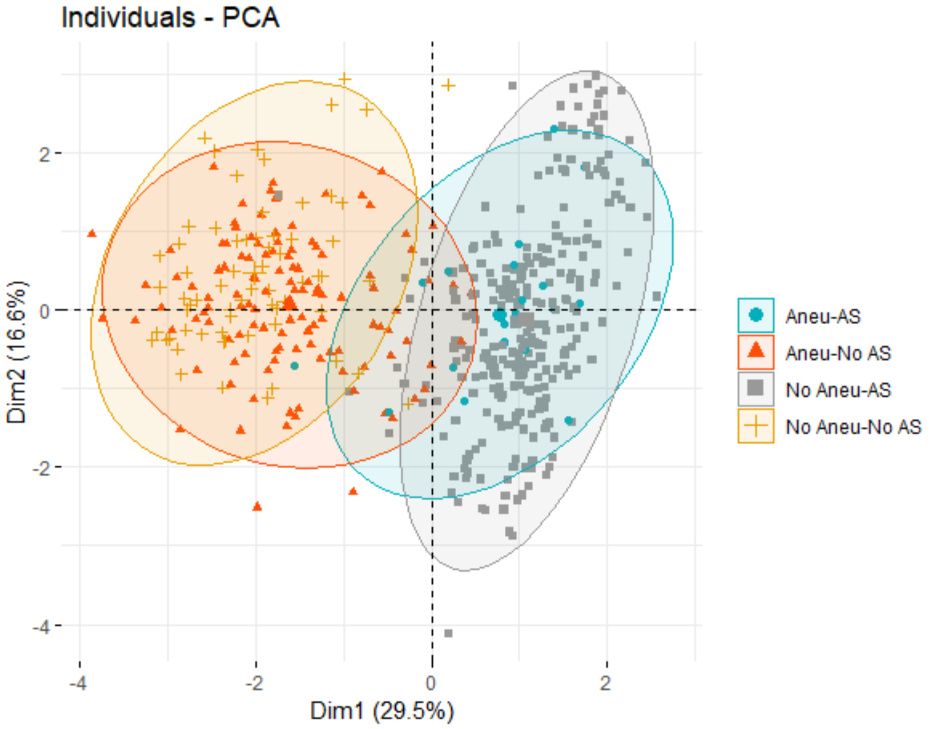

e-Figure 5. The ROC curve for our risk prediction model across the predictive methods without AS as predictor

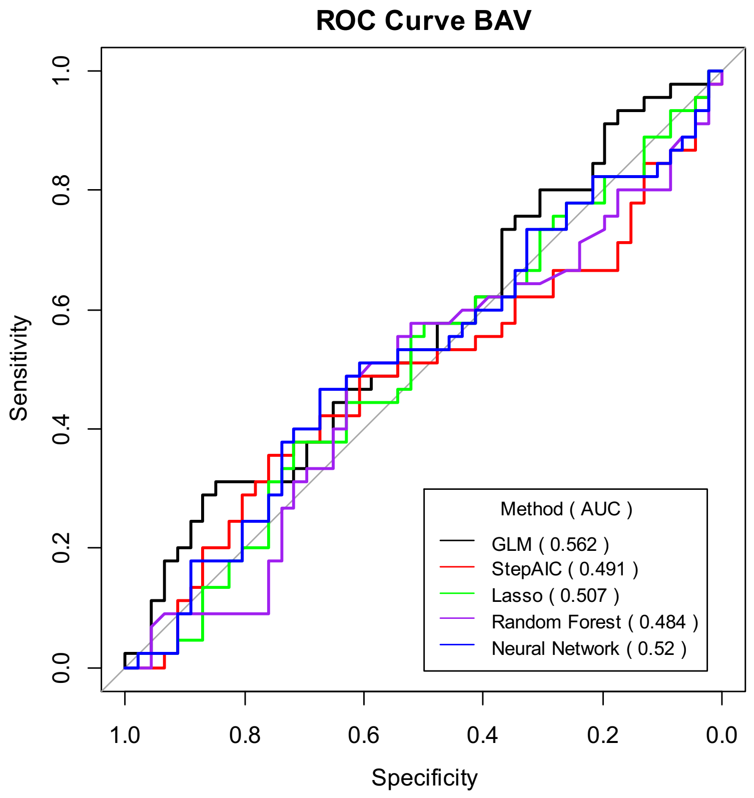

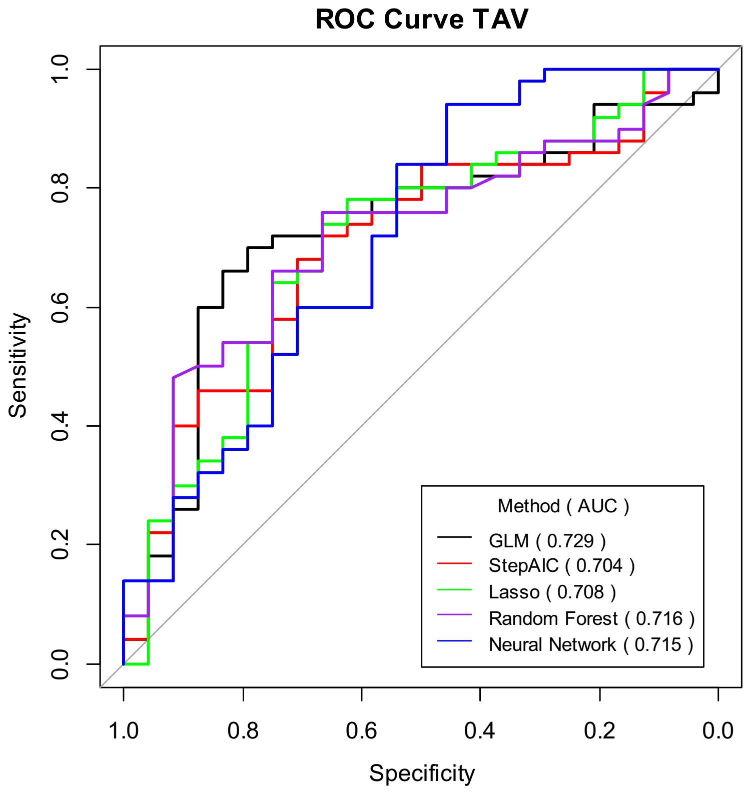

STROBE Statement—Checklist of items that should be included in reports of ***cohort studies***

	Item No	Recommendation	Page No
**Title and abstract**	1	(*a*) Indicate the study's design with a commonly used term in the title or the abstract	1
		(*b*) Provide in the abstract an informative and balanced summary of what was done and what was found	2
**Introduction**			
Background/rationale	2	Explain the scientific background and rationale for the investigation being reported	4
Objectives	3	State specific objectives, including any prespecified hypotheses	4
**Methods**			
Study design	4	Present key elements of study design early in the paper	5
Setting	5	Describe the setting, locations, and relevant dates, including periods of recruitment, exposure, follow-up, and data collection	5
Participants	6	(*a*) Give the eligibility criteria, and the sources and methods of selection of participants. Describe methods of follow-up	5
		(*b*) For matched studies, give matching criteria and number of exposed and unexposed	
Variables	7	Clearly define all outcomes, exposures, predictors, potential confounders, and effect modifiers. Give diagnostic criteria, if applicable	5, 6, 7,
Data sources/ measurement	8*	For each variable of interest, give sources of data and details of methods of assessment (measurement). Describe comparability of assessment methods if there is more than one group	5, 6, 7
Bias	9	Describe any efforts to address potential sources of bias	5, 6
Study size	10	Explain how the study size was arrived at	e-fig 1
Quantitative variables	11	Explain how quantitative variables were handled in the analyses. If applicable, describe which groupings were chosen and why	5, 6, 7
Statistical methods	12	(*a*) Describe all statistical methods, including those used to control for confounding	5, 6, 7
		(*b*) Describe any methods used to examine subgroups and interactions	5, 6, 7
		(*c*) Explain how missing data were addressed	
		(*d*) If applicable, explain how loss to follow-up was addressed	
		(*e*) Describe any sensitivity analyses	7, 8, 13
**Results**			
Participants	13*	(a) Report numbers of individuals at each stage of study—eg numbers potentially eligible, examined for eligibility, confirmed eligible, included in the study, completing follow-up, and analysed	8, 9
		(b) Give reasons for non-participation at each stage	
		(c) Consider use of a flow diagram	
Descriptive data	14*	(a) Give characteristics of study participants (eg demographic, clinical, social) and information on exposures and potential confounders	8, 9, 10
		(b) Indicate number of participants with missing data for each variable of interest	9
		(c) Summarise follow-up time (eg, average and total amount)	
Outcome data	15*	Report numbers of outcome events or summary measures over time	

For peer review only - http://bmjopen.bmj.com/site/about/guidelines.xhtml

Main results	16	(*a*) Give unadjusted estimates and, if applicable, confounder-adjusted estimates and their precision (eg, 95% confidence interval). Make clear which confounders were adjusted for and why they were included<br>(*b*) Report category boundaries when continuous variables were categorized<br>(*c*) If relevant, consider translating estimates of relative risk into absolute risk for a meaningful time period	11, 12, 13
Other analyses	17	Report other analyses done—eg analyses of subgroups and interactions, and sensitivity analyses	
**Discussion**			
Key results	18	Summarise key results with reference to study objectives	13, 14
Limitations	19	Discuss limitations of the study, taking into account sources of potential bias or imprecision. Discuss both direction and magnitude of any potential bias	14, 15, 19
Interpretation	20	Give a cautious overall interpretation of results considering objectives, limitations, multiplicity of analyses, results from similar studies, and other relevant evidence	15, 16, 17
Generalisability	21	Discuss the generalisability (external validity) of the study results	17, 18
**Other information**			
Funding	22	Give the source of funding and the role of the funders for the present study and, if applicable, for the original study on which the present article is based	19

*Give information separately for exposed and unexposed groups.

Note: An Explanation and Elaboration article discusses each checklist item and gives methodological background and published examples of transparent reporting. The STROBE checklist is best used in conjunction with this article (freely available on the Web sites of PLoS Medicine at http://www.plosmedicine.org/, Annals of Internal Medicine at http://www.annals.org/, and Epidemiology at http://www.epidem.com/). Information on the STROBE Initiative is available at http://www.strobe-statement.org.

For peer review only

BMJ Open

Clinical classifiers to identify ascending aortic dilatation in patients with bicuspid versus tricuspid aortic valves

Journal:	*BMJ Open*
Manuscript ID	bmjopen-2022-067977.R1
Article Type:	Original research
Date Submitted by the Author:	16-Jun-2023
Complete List of Authors:	Gaye, Bamba; INSERM U970, ; Universite de Paris Faculte de Sante,<br>Vignac, Maxime; Karolinska Institutet, Cardiovascular Medicine Unit, Center for Molecular Medicine, Department of Medicine<br>Gådin, Jesper R.; Karolinska Institutet, Cardiovascular Medicine Unit, Center for Molecular Medicine, Department of Medicine<br>Ladouceur, Magalie; INSERM; Georges Pompidou European Hospital Anatomical Pathology<br>Caidahl, Kenneth; Karolinska Institutet, Clinical Physiology Unit, Department of Molecular Medicine and Surgery<br>Olsson, Christian; Karolinska Institutet, Cardiothoracic Surgery Unit, Department of Molecular Medicine and Surgery<br>Franco-Cereceda, Anders; Karolinska Institutet, Cardiothoracic Surgery Unit, Department of Molecular Medicine and Surgery<br>Eriksson, Per ; Karolinska Institutet, Cardiovascular Medicine Unit, Center for Molecular Medicine, Department of Medicine<br>Björck, Hanna; Karolinska Institutet, Cardiovascular Medicine Unit, Center for Molecular Medicine, Department of Medicine
**Primary Subject Heading**:	Cardiovascular medicine
Secondary Subject Heading:	Surgery, Cardiovascular medicine
Keywords:	Valvular heart disease < CARDIOLOGY, EPIDEMIOLOGY, Cardiology < INTERNAL MEDICINE

SCHOLARONE™
Manuscripts

I, the Submitting Author has the right to grant and does grant on behalf of all authors of the Work (as defined in the below author licence), an exclusive licence and/or a non-exclusive licence for contributions from authors who are: i) UK Crown employees; ii) where BMJ has agreed a CC-BY licence shall apply, and/or iii) in accordance with the terms applicable for US Federal Government officers or employees acting as part of their official duties; on a worldwide, perpetual, irrevocable, royalty-free basis to BMJ Publishing Group Ltd ("BMJ") its licensees and where the relevant Journal is co-owned by BMJ to the co-owners of the Journal, to publish the Work in this journal and any other BMJ products and to exploit all rights, as set out in our licence.

The Submitting Author accepts and understands that any supply made under these terms is made by BMJ to the Submitting Author unless you are acting as an employee on behalf of your employer or a postgraduate student of an affiliated institution which is paying any applicable article publishing charge ("APC") for Open Access articles. Where the Submitting Author wishes to make the Work available on an Open Access basis (and intends to pay the relevant APC), the terms of reuse of such Open Access shall be governed by a Creative Commons licence – details of these licences and which Creative Commons licence will apply to this Work are set out in our licence referred to above.

Other than as permitted in any relevant BMJ Author's Self Archiving Policies, I confirm this Work has not been accepted for publication elsewhere, is not being considered for publication elsewhere and does not duplicate material already published. I confirm all authors consent to publication of this Work and authorise the granting of this licence.

For peer review only

Clinical classifiers to identify ascending aortic dilatation in patients with bicuspid versus tricuspid aortic valves

Bamba Gaye[1*] (PhD), Maxime Vignac[1] (MSc), Jesper R. Gådin[1] (PhD), Magalie Ladouceur[2,3] (MD), Kenneth Caidahl[4] (MD, PhD), Christian Olsson[5] (MD, PhD), Anders Franco-Cereceda[5] (MD), Per Eriksson[1] (PhD), Hanna M. Björck[1*] (PhD)

Running Title: Clinical Classifiers in BAV versus TAV

Total Word Count: 3315

Affiliations:
[1]Cardiovascular Medicine Unit, Center for Molecular Medicine, Department of Medicine, Karolinska Institutet, Stockholm, Karolinska University Hospital, Solna, Sweden
[2]INSERM, U970, Paris Cardiovascular Research Center / Université Paris Descartes, Sorbonne Paris Cité, Faculté de Médecine, Paris, France
[3]AP-HP, Georges Pompidou European Hospital, Cardiology Department, Paris, France
[4]Clinical Physiology Unit, Department of Molecular Medicine and Surgery, Karolinska Institutet, Stockholm, Sweden
[5]Cardiothoracic Surgery Unit, Department of Molecular Medicine and Surgery, Karolinska Institutet, Stockholm, Sweden

Disclosure: All authors have nothing to disclose.

***Address for Correspondence:**
Dr. Bamba Gaye (email: bamba.gaye@inserm.fr)
Dr. Hanna Bjorck (email: hanna.bjorck@ki.se)
Cardiovascular Medicine Unit, BioClinicum J8:20, Visionsgatan 4, Karolinska University Hospital, SE-171 64 Solna, Sweden

For peer review only

STRENGTHS AND LIMITATIONS OF THIS STUDY

- Neither the underlying pathophysiology nor a valid prediction model of the development of the aneurysm is available in patients with bicuspid aortic valves (BAV).
- Using automated machine learning algorithms and classic logistic regression models we demonstrated that clinical predictors can predict ascending aortic dilatation in patients with tricuspid aortic valves (TAV) but not in patients with bicuspid aortic valves (BAV).
- The lack of good models to develop clinical classifiers of BAV-associated ascending aortic dilatation strengthen the focus of genetics and/or flow as important contributing factors to aneurysm development in these individuals.
- Our study is a single centre and mono- ethnicity study. Therefore our results may not be generalizable to other population of BAV/TAV.

For peer review only

For peer review only - http://bmjopen.bmj.com/site/about/guidelines.xhtml

Abstract

Objective: We aimed to develop clinical classifiers to identify prevalent ascending aortic dilatation in patients with BAV and tricuspid aortic valve (TAV).

Methods: This study included BAV (n=543) and TAV (n=491) patients with aortic valve disease and/or ascending aortic dilatation but devoid of coronary artery disease undergoing cardiothoracic surgery. We applied machine learning algorithms and classic logistic regression models, using multiple variable selection methodologies to identify predictors of high risk of ascending aortic dilatation (ascending aorta with a diameter above 40 mm). Analyses included comprehensive multidimensional data (i.e., valve morphology, clinical data, family history of cardiovascular diseases, prevalent diseases, demographic, lifestyle and medication).

Results: BAV patients were younger (60.4 ± 12.4 years) than TAV patients (70.4±9.1 years), and had a higher frequency of aortic dilatation (45.3% vs. 28.9% for BAV and TAV, respectively. P<0.001). The aneurysm prediction models showed mean AUC values above 0.8 for TAV patients, with the absence of aortic stenosis being the main predictor, followed by diabetes and high sensitivity C-Reactive Protein. Using the same clinical meaures in BAV patients our prediction model resulted in AUC values between 0.5-0.55, not useful for prediction of aortic dilatation. The classiffication results were consistent for all machine learning algorithms and classic logistic regression models.

Conclusions: Cardiovascular risk profiles appear to be more predictive of aortopathy in TAV patients than in patients with BAV. This adds evidence to the fact that BAV- and TAV-associated aortopathy involve different pathways to aneurysm formation and highlights the need for specific aneurysm preventions in these patients. Further, our results highlight that machine learning approaches do not outperform classical prediction methods in addressing complex interactions and non-linear relations between variables.

Keywords: Bicuspid Aortic Valve; Tricuspid Aortic Valve; Ascending Aortic Aneurysm; Aortic Dilation

For peer review only

Abbreviations

AAA	Abdominal Aortic Aneurysm
AR	Aortic Regurgitation
AS	Aortic Stenosis
ASAP	Advanced Study of Aortic Pathology
BAV	Bicuscpid Aortic Valve
BMI	Body Mass Index
BSA	Body Surface Area
CVD	Cardiovascular Disease
DAVAACA	Disease of the Aortic Valve Ascending Aorta and Coronary Arteries
FFR	Fractional Flow Reserve
Hs-CRP	High Sensitivity C-Reactive Protein
LDL	Low Density Lipoprotein
LN	Left- and Non-Coronary Cusps
MCA	Multiple Correspondence Analysis
PCA	Principal ComponentAanalysis
PP	Pulse Pressure
TAA	Thoracic Aortic Aneurysm
TAV	Tricuspid Aortic Valve
TEE	Transesophageal Echocardiography
TTE	Transthoracic Echocardiography

For peer review only

Introduction

Thoracic aortic aneurysm (TAA) is a silent disease characterized by medial degradation and pathological widening of the intrathoracic aorta. Individuals with a bicuspid aortic valve (BAV) are at higher risk of developing ascending aortic aneurysm than individuals born with a normal tricuspid aortic valve (TAV).[1,2] BAV is the most common congenital cardiac abnormality with an incidence of about 13 per 1000 live births and more than one third of all BAV patients develop aneurysm or aortic dissection later in life.[3–5] The underlying mechanism is not known although a turbulent flow, caused by the abnormal bicuspid valve anatomy, and/or a genetic defect have been proposed as contributing factors. The complications of BAV have led to a number of unanswered clinical questions such as: (1) what mechanisms are underlying the development of aortopathy in adults with BAV, (2) how to predict the development of aortopathy in individuals with BAV, and (3) will prediction models performance and predictors be different in patients with BAV compared to patients with TAV. Echocardiography is effective to show the degree of the sinus valsava and the annulus dilatation. However, it is more difficult to know the degree of dilation of the sinotubular junction and the ascending aorta even though they are the more frequently dilatated sections in patients with BAV[6].

Our aim was to identify clinical classifiers of prevalent aortopathy using comprehensive multidimensional clinical data. Automated machine learning methods and traditional regression models was performed on a total of 1034 subjects with either BAV or TAV.

For peer review only

For peer review only - http://bmjopen.bmj.com/site/about/guidelines.xhtml

METHODS

Clinical cohorts

The Advanced Study of Aortic Pathology (ASAP) study design and description have been previously published.[1,7] Briefly, the ASAP cohort is a single-center, observational cohort study of consecutive patients with aortic valve and/or ascending aortic disease undergoing elective open-heart surgery at the Cardiothoracic Surgery Unit, Karolinska University Hospital in Stockholm, Sweden. Inclusion criteria were patients aged 18 or above with aortic valve disease (i.e., aortic stenosis; AS or aortic regurgitation; AR) and/or ascending aorta dilatation (aneurysm or ectasia) but devoid of significant coronary artery disease (stenosis >70% or Fractional Flow Reserve (FFR) <0.80) and primarily not planned for another concomitant valve surgery. The Disease of the Aortic Valve Ascending Aorta and Coronary Arteries (DAVAACA) study was set up in the continuity of the ASAP study. The ASAP/DAVAACA study started in February 2007 and include multidimensional data (blood analyses, genetic and clinical data, family history of cardiovascular diseases, prevalent diseases data, demographic characteristics, lifestyle habits data and medication). In the present study, 1180 operated patients were included (June 28, 2017). Exclusion of patients with unicuspid aortic valves, patients with missing data for cuspidity and aortic dilatation and patients with syndromic forms of TAA resulted in a final study population of 1034 subjects (e-Figure 1). Data collection and classification of AS and AR have been previously described.[1] Aortic valve function and aortic dimensions were assessed according to the standards outlined by the American Society of Echocardiography.[8–10]

For peer review only - http://bmjopen.bmj.com/site/about/guidelines.xhtml

Echocardiography

Preoperative transthoracic echocardiography (TTE) was used to determine aortic valve function. Transesophageal echocardiography (TEE) was performed on the operating table, prior to surgery, under general anaesthesia and as previously described.[11] The echocardiographic evaluation has been already pulished elsewhere.[1] TEE evaluation was used to assess valvular function, aortic root morphology and ascending aortic diameter; measurements were performed according to standards outlined by the ASE. Diameter of the ascending aorta was measured at several locations (annulus, sinus Valsalva, STJ. An ascending aorta with a diameter above 40 mm was classified as aortic dilatation in both women and men. Body surface area (BSA) was calculated using Du Bois method.[12] Aortic stenosis and aortic regurgitation were defined according to standard guidelines for valve surgery.[13]

Aortic valve morphology

The morphology of the aortic valve was determined by inspection of the valve during surgery. Based on appearance, the valve was classified according to number of cusps and commissures. Three cusps and three commissures denote a TAV; two cusps and two commissures denote a BAV (if a remnant commissural raphe was present) or true BAV (if no raphe was present). The BAV was further classified according to cusps fusion; right- and left coronary cusps (RL), right- and non-coronary cusps (RN) or left- and non-coronary cusps (LN).[1] The Sievers classification[14], based on intraoperative analysis of the aortic valve morphology, was used to diagnose and type the bicuspid aortic valve.

Statistical analysis

Characteristics of the population were described using ANOVA or Chi-square tests when appropriate. Several meaningful blocks of variables were chosen (clinical data, family history

For peer review only - http://bmjopen.bmj.com/site/about/guidelines.xhtml

of cardiovascular diseases, prevalent diseases, demographic characteristics, lifestyle habits data and medication). Principal component analysis (PCA) and Multiple correspondence analysis (MCA) were used to explain the variance-covariance structure of the variables combined. To identify variables that separated dilated versus non-dilated BAV and TAV patients, logistic regression models were used and automated machine learning algorithms were applied.

Logistic Regression

For logistic regression, a set of variables were selected to be included in the model. Since model performance can rely heavily on variable selection, different variable selection methods were tested prior to the logistic regression analysis (e-Figure 2). Firstly, variables were selected based on prior knowledge and/or biologic plausibility. Then, two automated variable selection methods were considered: (i) backward and forward elimination to optimize Akaike Information Criteria[11], (ii) and selection operator (LASSO).[12]

Machine Learning Algorithms

We used two machine learning algorithms, Random Forests and Artificial Neural Network, which are among the most widely and successfully used for clinical data.[15,16] Each one of them represents a different algorithm "family", with different internal algorithm structures.[17] Since it was not known beforehand which kind of algorithm would perform best, algorithms with different internal structures were chosen to increase the probability of good discriminative performance. A 10-fold cross validation method to our logistic regression was also applied, allowing to split the training data into five subsets. Then, five times iteration over the five subsets were performed, such that the subset with the index same as the iteration number was used as the validation test and the remaining 4 subsets were training tests. Sensitivity,

For peer review only - http://bmjopen.bmj.com/site/about/guidelines.xhtml

specificity, and positive and negative predictive values for each predictive model were also assessed, applying a leave one out cross validation method (e-Figure 3, e-Figure 4).

Patient and Public Involvement statement

It was not possible to involve patients or the public in the design, or conduct, or reporting, or dissemination plans of our research.

RESULTS

Characteristics of BAV and TAV patients

The study population included a total of 1034 patients (52% BAV and 48% TAV) (e-Table 1, e-Figure 1). BAV patients were significantly younger (60.4 (SD 12.40) years vs. 70.4 (SD 9.09) years in TAV patients), and mainly men (73.1%) compared to TAV patients (63.3%). Furthermore, BAV patients had significantly higher body surface area (BSA) but, in contrast, a lower body mass index (BMI). AS was more common in BAV compared to TAV whereas no significant difference in the proportion of AR was found, even if the prevalence of AR was slightly higher in TAV patients. Finally, a higher prevalence of cardiovascular disease (CVD) history was observed in TAV individuals (hypertension, coronary artery disease, heart failure and stroke) and a higher percentage of family history of CVD (sibling history of myocardial infarction before 65 years of age).

For peer review only

Table 1: Baseline Characteristics of Patients with BAV or TAV (dilated vs. non-dilated ascending aorta).

	BAV patients (n=543)			TAV patients (n=491)		
	Dilated (n=246)	Non-Dilated (n=297)	P value	Dilated (n=142)	Non-Dilated (n=349)	P value
**Gender: Male**	182 (74.0%)	215 (72.4%)	0.749	89 (62.7%)	222 (63.6%)	0.927
**Age**	61.1 (11.2)	59.8 (13.3)	0.204	67.2 (10.1)	71.8 (8.28)	<0.001
**Height**	177 (9.14)	174 (9.29)	0.002	174 (9.76)	172 (9.77)	0.043
**Weight**	83.1 (15.6)	80.9 (15.8)	0.100	81.4 (16.3)	81.6 (15.7)	0.909
**BSA**	2.00 (0.21)	1.96 (0.22)	0.052	1.95 (0.22)	1.94 (0.22)	0.479
**BMI**	26.5 (4.15)	26.6 (4.36)	0.916	26.9 (4.41)	27.7 (4.90)	0.087
**Regular Smoker:**			0.176			0.537
**No**	108 (44.1%)	142 (48.8%)		61 (43.3%)	152 (43.8%)	
**Former**	117 (47.8%)	117 (40.2%)		69 (48.9%)	177 (51.0%)	
**Yes**	20 (8.16%)	32 (11.0%)		11 (7.80%)	18 (5.19%)	
**Raphe:**			0.568			
**True BAV**	24 (9.96%)	23 (7.96%)				
**Left-Non coronary**	1 (0.41%)	4 (1.38%)				
**Right-Non coronary**	43 (17.8%)	47 (16.3%)				
**Right-Left**	173 (71.8%)	215 (74.4%)				
**Systolic Blood Pressure**	135 (18.2)	136 (18.9)	0.299	144 (17.4)	142 (21.5)	0.299
**Diastolic Blood Pressure**	81.3 (12.3)	79.0 (11.6)	0.026	75.1 (13.0)	76.5 (13.3)	0.290
**Pulse Pressure (PP)**	53.3 (16.5)	57.2 (18.3)	0.009	68.7 (18.1)	65.4 (20.3)	0.076
**Leukocytes**	5.70 [4.70;6.80]	5.90 [4.97;7.00]	0.052	6.00 [5.00;7.30]	6.10 [5.10;7.40]	0.386
**hsCRP**	0.93 [0.50;2.15]	1.20 [0.59;2.80]	0.056	2.10 [0.98;4.05]	1.60 [0.71;3.80]	0.050
**Cholesterol**	4.90 [4.20;5.70]	4.80 [4.20;5.60]	0.566	4.80 [4.12;5.40]	4.60 [3.90;5.60]	0.175
**LDL**	2.90 [2.20;3.60]	2.80 [2.30;3.60]	0.813	2.90 [2.20;3.50]	2.60 [1.90;3.40]	0.064
**Aortic Stenosis:**	169 (68.7%)	235 (79.1%)	0.008	23 (16.2%)	283 (81.1%)	<0.001
**Aortic Regurgitation:**	83 (33.7%)	75 (25.3%)	0.038	93 (65.5%)	67 (19.2%)	<0.001
**Diameter Aortic Anulus**	25.5 (3.41)	24.2 (3.58)	<0.001	23.5 (3.17)	22.8 (3.03)	0.042
**Diameter Sinus Valsalva**	38.1 (5.38)	33.6 (5.11)	<0.001	40.1 (6.98)	32.8 (5.71)	<0.001
**Diameter STJ**	33.6 (5.63)	28.2 (4.51)	<0.001	35.9 (6.76)	26.6 (4.36)	<0.001
**Diameter Ascend Aorta**	46.9 (5.02)	33.0 (4.27)	<0.001	50.5 (7.90)	31.6 (4.03)	<0.001
**Sibling MI before 65 year**	13 (5.56%)	19 (6.81%)	0.688	11 (8.09%)	35 (10.8%)	0.474
**Mother MI before 65 year**	15 (6.20%)	10 (3.64%)	0.250	7 (5.26%)	25 (7.67%)	0.474
**Father MI before 65 year**	24 (10.6%)	45 (16.3%)	0.087	18 (14.1%)	45 (14.4%)	0.922
**Myocardial Infarction**	8 (3.25%)	11 (3.77%)	0.930	9 (6.43%)	33 (9.46%)	0.367
**Stroke**	15 (6.10%)	15 (5.08%)	0.746	14 (10.0%)	43 (12.3%)	0.571
**AAA**	23 (9.39%)	2 (0.68%)	<0.001	25 (18.2%)	5 (1.44%)	<0.001
**Angina Pectoris**	9 (3.66%)	26 (8.87%)	0.023	9 (6.43%)	52 (14.9%)	0.015
**Heart Failure**	15 (6.15%)	25 (8.65%)	0.354	15 (10.6%)	42 (12.2%)	0.747
**Hypertension**	115 (47.1%)	140 (47.6%)	0.979	88 (62.9%)	223 (65.0%)	0.730
**Diabetes**	15 (6.10%)	39 (13.3%)	0.009	3 (2.16%)	61 (17.5%)	<0.001
**Anti hypertensive drugs***						
**ACE inhibitor**	46 (18.7%)	57 (19.3%)	0.941	45 (31.9%)	97 (27.9%)	0.434
**Aspirin**	48 (19.5%)	84 (28.5%)	0.021	38 (27.0%)	155 (44.8%)	0.001
**Betablocker**	92 (37.4%)	125 (42.4%)	0.277	76 (53.9%)	179 (51.3%)	0.672

Chi square test was used for categorical variables; ANOVA for normal-distributed continuous variables; Kruskal-Wallis test for non-normal continuous variables. BSA, body surface area; BMI, body mass index; hsCRP, high sensitive C-reactive

For peer review only

protein; LDL, low density lipoprotein; STJ, sinotubular junction; AAA, abdominal aortic aneurysm. ****Information on other anti-hypertensive drugs is presented in e- table 6.***

For peer review only

For peer review only - http://bmjopen.bmj.com/site/about/guidelines.xhtml

Aortic dilatation in BAV and TAV patients

Globally, PCA showed no separation among BAV patients with (BAV-D) or without (BAV-ND) ascendic aortic dilatation (Figure 1, Panel A), using general clinical characteristics, family history of CVD, prevalent diseases and demographics as input. By contrast, TAV patients with a dilated (TAV-D) ascending aorta clearly separated from TAV individuals with non-dilated (TAV-ND) ascending aortas (Figure 1, Panel B), indicating a more pronounced difference between these groups.

Analysis of patient descriptive data showed a pattern of significantly different associated traits between BAV-ND vs. BAV-D and TAV-ND vs. TAV-D (Table 1, e-Table 2) patients. Specifically, TAV-D patients were younger than TAV-ND patients while in the BAV group age did not differ. BAV-D patients, on the other hand, had a higher BSA and diastolic blood pressure, and a lower pulse pressure (PP) than BAV-ND patients. In both BAV and TAV patients, AR was more commonly associated with aortic dilatation, whilst AS was more prevalent in patients with non-dilated aorta. It was also noted that aortic dilatation was more localized to the tubular part of the ascending aorta in BAV patients, whereas in TAV patients the dilatation more often included the aortic root (Table 1). A positive association between dilatation and abdominal aortic aneurysm (AAA), and negative associations between dilatation and diabetes, and dilatation and angina was found in both BAV and TAV patients (Table 1).

To further analyze BAV- and TAV-associated aortopathy, a multivariable logistic regression model was performed, including age, sex, BSA, low density lipoprotein (LDL) cholesterol and high sensitivity C-Reactive Protein (hs-CRP) as covariates (Table 2). Independent variables were choosen based on previous analyses with significant differences within groups. Covariates were selected based on stepwise selection and decision trees. The results showed that in BAV

For peer review only

patients, AS, PP and diabetes were negatively associated with dilatation (adjusted models for age, sex, BSA, LDL and hsCRP were with AS: OR=0.44 (0.28-0.70), P=0.001; PP: OR=0.99 (0.98-1.0), P=0.025, and diabetes: OR=0.32 (0.16-0.62), P=0.001). Similarly, in TAV patients, dilatation was negatively associated with AS: OR=0.03 (0.02-0.06), P<0.001 and diabetes OR=0.11 (0.03-0.32), P<0.001. The magnitude of the association with AS was however 15-fold higher in TAV than in BAV patients. Aortic regurgitation was positively associated with aortic dilation in both BAV and TAV with a 5-fold higher magnitude in TAV compared to BAV patients. (Table 2). The ROC curve for the risk prediction model across the predictive methods without AS as predictor are shown in e-Figure 5.

For peer review only

Table 2. Predictors of ascending aortic dilatation in BAV and TAV patients, separately. Univariate and Multivariate logistic regression analysis.

	BAV						TAV					
	unAdjusted Model		Model 1		Model 2		unAdjusted Model		Model 1		Model 2	
	OR (95% IC)*	p	OR (95% IC)*	p	OR (95% IC)*	p	OR (95% IC)*	p	OR (95% IC)*	p	OR (95% IC)*	p
**Aortic Stenosis**	0.58 (0.39-0.85)	0.006	0.44 (0.28-0.70)	0.001	0.46 (0.24-0.88)	0.020	0.05 (0.03-0.07)	<0.001	0.03 (0.02-0.06)	<0.001	0.02 (0.01-0.05)	<0.001
**Aortic Regurgitation**	1.51 (1.04-2.19)	0.031	1.92 (1.25-2.99)	0.003	1.42 (0.76-2.66)	0.270	7.99 (5.19-12.45)	<0.001	7.47 (4.62-12.27)	<0.001	0.58 (0.23-1.36)	0.229
**Pulse Pressure**	0.99 (0.98-1.00)	0.011	0.99 (0.98-1.00)	0.025	0.98 (0.97-0.99)	0.001	1.01 (1.00-1.02)	0.091	1.00 (0.99-1.02)	0.385	0.99 (0.97-1.00)	0.096
**Diabetes**	0.42 (0.22-0.77)	0.007	0.32 (0.16-0.62)	0.001	0.36 (0.18-0.72)	0.005	0.1 (0.03-0.29)	<0.001	0.11 (0.03-0.32)	<0.001	0.26 (0.06-0.87)	0.048

* Odds ratio and 95% confidence interval limits were obtained by logistic regression
Model 1: adjusted for Sex_Male + Age + BSA + LDL + hsCRP
Model 2: adjusted for Sex_Male + Age + BSA + LDL+ hsCRP + the 3 other variables (i.e: Aortic Regurgitation, Pulse Pressure and Diabetes for Aortic Stenosis)

For peer review only

Clinical classifiers of aortic dilatation

In order to answer the question if the prevalence of aortopathy in TAV and BAV patients may be identified using clinical characteristics, automated machine learning algorithms, including Random Forests and Artificial Neural Network, and classic logistic regression models were used. This showed that the discrimination between TAV-ND and TAV-D patients was good for all models tested, with mean AUCs ranging from 0.81 to 0.88 (Figure 2, e-Table 3). The best discrimination was obtained using Random Forest (mean AUC: 0.88 (95% CI: 0.78-0.96)) and GLM (mean AUC: 0.81 (95% CI: 0.68-0.93)) (Figure 2, e-Table 3). Generalized Linear Regressions included AS, AR, PP, prevalence of diabetes, age, sex, BSA, LDL, hsCRP, BMI as variables. It was not possible to predict aortic dilatation in BAV individuals that are at high risk of developing aneurysm, as indicated by poor discrimination by all classifications models used (Figure 2, e-Table 3). This is in line with the lack of separation in the PCA.

Of note, the robustness of our classification models is supported by the results of the sensitivity analyses using imputed data (e-Table 4, e-Table 5). Supplemental e-Table 5 indicates that the main characteristics between subjects with and without missing data did not differ substantially, minimizing selection bias.

Discussion

In our analysis of 1034 Swedish subjects with bicuspid and tricuspid aortic valves with and without prelevant aortopathy but devoid of coronary artery disease and primarily not planned for another concomitant valve surgery, we report two key findings. Firstly, prediction of aortic dilatation using automated machine learning methods and traditional regression models on multidimensional clinical data was possible only among TAV individuals, and *not* in patients with BAV. This suggests that general clinical cardiovascular risk profiles play more important roles during aortic dilation in TAV patients than in patients with a BAV, and further supports

For peer review only - http://bmjopen.bmj.com/site/about/guidelines.xhtml

that aortopathy associated with BAV and TAV, respectively, are clearly distinct with different underlying etiologies. Secondly, our study shows that the classification results were consistent for all machine learning algorithms and classic logistic regression models. This suggest that machine learning approaches might not outperform classical prediction methods in addressing complex interactions and non-linear relations between variables.

The strongest predictor for ascending aortic dilatation in TAV was the absence of aortic stenosis. The other contributors to the prediction of aortic dilatation in TAV are shown in **e-Table 6.**

This is in accordance with our previous observations that surgical patients with AS and ascending aortic dilatation almost exclusively have a BAV[18]. Whether this imply that biological processes associated with the development of AS in TAV also contributes to ascending aortic stability needs to be further elucidated. AS is commonly caused by progressive calcification of the aortic valve and increase in prevalence with age. In our cohort, TAV patients with dilated ascending aortas were significantly younger than TAV patients with non-dilated ascending aortas, which may be one contributing factor to the observed association and possibly reflects the surgical nature of the cohort. However, omitting patients with AS from the analysis still resulted in significant predictive values, although with worse discrimination, indicating that age alone cannot explain the association. Other strong contributors to the prediction model were diabetes and hsCRP. A negative association between diabetes and aneurysm in the abdominal aorta is well documented.[19] It has also been suggested that metformin prescription may associate with decreased risk of aortic dilatation and that the molecular mechanism involves a metformin-induced reduction in aortic inflammation.[20,21] Similarly, an elevated hsCRP has been described in AAA patients and found to be an independent risk factor for AAA.[22,23] Moreover, we have previously shown an inflammatory gene expression profile in dilated aortic

For peer review only

For peer review only - http://bmjopen.bmj.com/site/about/guidelines.xhtml

tissue from TAV but not BAV patients.[7] In the present study, there was a borderline significance of reduced hsCRP levels in BAV patients with dilated aorta. This together implies that aortic dilatation in TAV, in these aspects, may be more similar to aneurysm of the abdominal aorta than BAV aortic dilatation.

The lack of a good model for risk prediction of BAV-associated aortopathy raises the question, which other contributing factors may be of importance for aneurysm formation and development in these individuals? Two main hypotheses have been put forth in the literature.

Firstly, an altered flow in the proximal part of the ascending aorta due to the valve malformation itself has been suggested to provoke aortic dilatation. Also, different BAV morphotypes, i.e. cusp fusion pattern, have been shown to cause flow disturbances that affect the aorta in morphotype-dependent ways. [24] In our study, flow characteristics were not included in the predition model, which possibly could have influenced the results. However, we could not see any difference in the precense of aortic dilatation between different morphotypes, i.e. true BAV, LN-, RN- or RL cusp fucion (Table 1). Of note, other factors may also influence aortic flow patterns, e.g. eccentricity of valve opening due to valve disease, or vessel stiffnes. Indeed, it has been suggested that AS significantly alters aortic hemodynamic and wall shear stress, independent of aortic valve phenotype.

Secondly, the genetic contribution may override the influence of traditional risk factors to aneurysm development in patients with BAV. Although specific gene(s) and/or mutation(s) underlying BAV and BAV-associated aortopathy are still to be unravelled, several genes have been shown to be associated with both BAV formation and concomitant aortopathy in mice and humans.[25,26,27(p2)] Moreover, a high heratibility of BAV and/or other cardiovascular

For peer review only - http://bmjopen.bmj.com/site/about/guidelines.xhtml

malformations have been demonstrated using segregation patterns in families, with a heritability of BAV and BAV together with other cardiovascular malformations being as high as 89% and 75%, respectively.[28] A third, most likely, possibility is that both genetic factors and abnormal hemodynamic burden play central roles in BAV-associated aortopathy, interacting with each other and therby contribute to aortic dilatation.

Further dissecting differences between patients with non-dilated and dilated ascending aortas we found that, among others, PP, AS, AR and diabetes were associated with dilatation in BAV. PP is a well known risk factor for cardiovascular disease and the clinical manifestation of increased vascular stiffness.[29] Surprisingly, PP was higher in BAV patients with a non-dilated aorta, which may seem counterintuative since AR is associated with both increased PP and aortic dilatation. However, it may be speculated that the higher PP seen in these patients may rely on structural changes due to an increased hemodynamic burden associated with a BAV. In line with this, we have previously showed that BAV patients have a qualitative collagen defect in their ascending aorta, signified by a different collagen glycation compared with TAV patients and suggestive of an altered non-enzymatic collagen crosslinking.[30] Interestingly, we have also shown that dilated ascending aorta of BAV patients display an increased collagen-related stiffness compared with TAV patients.[31] Furthermore, The Strong Heart Study could also show that in patients free of prevalent coronary heart disease, aortic root dilatation was, at a given diastolic blood pressure and stroke volume, associated with lower pulse pressure.[32]

Additionally, accelerated vascular aging and increased arterial stiffness has previously been described in diabetic patients, the proportion of which was higher among BAV patients with non-dilated aortas.[33] However, we and others have previously demonstrated an increased vascular inflammation in dilated aorta in TAV- but not BAV patients, suggesting that other

For peer review only - http://bmjopen.bmj.com/site/about/guidelines.xhtml

mechanisms could be involved in the protective role of diabetes on aneurysm formation in BAV patients.[7,34] The association between dilatation and valve disease was not as pronounced in BAV- as in TAV patients, although AS was also negatively associated with dilatation in BAV, as previously found.[35] It may be speculated that the presence of AS increases flow velocities and blood pressure in the ascending aorta, thereby stimulating vascular remodeling and strengthening of the aortic wall. Whether this hamper the process of dilatation remains to be answered. The relation between degree of stenosis and width of the ascending aorta is complex, and a previous study found mid-ascending dilatation proportional to valve gradient when patients with small aortas were excluded.[35]

Our findings raise the issue of how to identify and implement prevention of aortopathy in BAV patients in a clinical setting. So far, clinicians have focused on aortic valve function and aortic dimensions to indicate cardiac surgery and recommend annual follow-up in asymptomatic patients to screen for associated aortopathy.[36] High importance has been given to the morphology of the valve, although in our study, dilatation in BAV did not show any significant association with valve morphology. Of note, previous studies establishing an association between BAV cusp fusion and clinical outcomes relied on small sample size based on imaging diagnostic rather than anatomic diagnosis.[37]

Study strengths and limitations

In this study, comprehensive clinical data, including blood sampling as well as epidemiological data was used in the analysis of in total 1034 individuals (543 BAV and 491 TAV). The morphology of aortic valves was evaluated by visual inspection during open-heart surgery, which is a major strength compared to only echocardiography in terms of reliability.

For peer review only

A few limitations must however be highlighted. Firstly, by design, only individuals devoid of significant coronary artery disease were included, which may introduce a selection bias and an overestimation of the prevalence of subjects with aneurysm. Secondly, as this is a population based surgical cohort, it is possible that our study population included BAV and TAV patients with worse outcomes compared to their counterparts of similar age. This should however not affect the associations between valve type and aortic dilatation. Thirdly, TAV patients with non-dilated aortas were significantly older than TAV individuals with a dilated aorta, which could possibly explain the higher degree of patients with aortic stenosis in this group. Lastly our study is a single centre and mono- ethnicity study. Therefore our results may not be generalizable to other population of BAV/TAV.

To conclude, using automated machine learning algorithms and classic logistic regression models we demonstrated that in TAV patients, cardiovascular risk profiles appear to be more predictive of aortopathy than in BAV patients. The good performance of the TAV classifier also after exclusion of AS offers important implications for better targeting TAV individuals that are of a high risk of developing aneurysm. The lack of good models to develop clinical classifiers of BAV-associated aortopathy strengthen the focus of genetics and/or flow as important contributing factors to aneurysm development in these individuals.

For peer review only

Contributorship statement

Author Contributions: Gaye, Franco-Cereceda, Eriksson, and Björck had full access to all the data in the study and take responsibility for the integrity of the data and the accuracy of the data analysis.

Study concept and design: Gaye, Eriksson, Björck

Acquisition, analysis, or interpretation of data: Gaye, Vignac, Gådin, Ladouceur, Caidahl, Olsson, Franco-Cereceda, Eriksson, Björck

Drafting of the manuscript: Gaye

Critical revision of the manuscript for important intellectual content: Gaye, Vignac, Gådin, Ladouceur, Caidahl, Olsson, Franco-Cereceda, Eriksson, Björck

Statistical analysis: Gaye and Vignac

Study supervision: Gaye, Eriksson, Björck

Competing interests

The authors declare no competing interests

Funding

The work was supported by the Swedish Research Council [12660]; the Swedish Heart-Lung Foundation [20180451, 20170669]; the Stockholm County Council [20180072]; La Marató de TV3 [20151330]; and a donation by Fredrik Lundberg.

Data sharing statement

The data of this article are available upon reasonable request to the corresponding author.

Ethics Approval Statement

For peer review only

The study was approved by the Regional Ethics Review Board (application number 2006/784-31/1; 2012/1633-31/4), Stockholm, Sweden. Oral and written informed consent were obtained from all patients according to the declaration of Helsinki.

For peer review only

References

1. Jackson V, Petrini J, Caidahl K, Eriksson MJ, Liska J, Eriksson P, Franco-Cereceda A. Bicuspid aortic valve leaflet morphology in relation to aortic root morphology: a study of 300 patients undergoing open-heart surgery. *Eur J Cardiothorac Surg*. Published online May 2011:S1010794011004623. doi:10.1016/j.ejcts.2011.04.014

2. Messner B, Bernhard D. Bicuspid aortic valve-associated aortopathy: Where do we stand? *J Mol Cell Cardiol*. 2019;133:76-85. doi:10.1016/j.yjmcc.2019.05.023

3. Ward C. Clinical significance of the bicuspid aortic valve. *Heart*. 2000;83(1):81-85. doi:10.1136/heart.83.1.81

4. Williams DS. Bicuspid aortic valve. *J Insur Med N Y N*. 2006;38(1):72-74.

5. Losenno KL, Goodman RL, Chu MWA. Bicuspid Aortic Valve Disease and Ascending Aortic Aneurysms: Gaps in Knowledge. *Cardiol Res Pract*. 2012;2012:1-16. doi:10.1155/2012/145202

6. Verma S, Siu SC. Aortic dilatation in patients with bicuspid aortic valve. *N Engl J Med*. 2014;370(20):1920-1929. doi:10.1056/NEJMra1207059

7. Folkersen L, Wågsäter D, Paloschi V, Jackson V, Petrini J, Kurtovic S, Maleki S, Eriksson MJ, Caidahl K, Hamsten A, Michel J-B, Liska J, Gabrielsen A, Franco-Cereceda A, Eriksson P. Unraveling Divergent Gene Expression Profiles in Bicuspid and Tricuspid Aortic Valve Patients with Thoracic Aortic Dilatation: The ASAP Study. *Mol Med*. 2011;17(11-12):1365-1373. doi:10.2119/molmed.2011.00286

8. Lang RM, Bierig M, Devereux RB, Flachskampf FA, Foster E, Pellikka PA, Picard MH, Roman MJ, Seward J, Shanewise JS, Solomon SD, Spencer KT, St John Sutton M, Stewart WJ. Recommendations for Chamber Quantification: A Report from the American Society of Echocardiography's Guidelines and Standards Committee and the Chamber Quantification Writing Group, Developed in Conjunction with the European Association of Echocardiography, a Branch of the European Society of Cardiology. *J Am Soc Echocardiogr*. 2005;18(12):1440-1463. doi:10.1016/j.echo.2005.10.005

9. Zoghbi W. Recommendations for evaluation of the severity of native valvular regurgitation with two-dimensional and doppler echocardiography. *J Am Soc Echocardiogr*. 2003;16(7):777-802. doi:10.1016/S0894-7317(03)00335-3

10. Baumgartner H, Hung J, Bermejo J, Chambers JB, Evangelista A, Griffin BP, Iung B, Otto CM, Pellikka PA, Quiñones M. Echocardiographic Assessment of Valve Stenosis: EAE/ASE Recommendations for Clinical Practice. *J Am Soc Echocardiogr*. 2009;22(1):1-23. doi:10.1016/j.echo.2008.11.029

11. Walter S, Tiemeier H. Variable selection: current practice in epidemiological studies. *Eur J Epidemiol*. 2009;24(12):733-736. doi:10.1007/s10654-009-9411-2

12. Tibshirani R. The lasso method for variable selection in the Cox model. *Stat Med*. 1997;16(4):385-395. doi:10.1002/(sici)1097-0258(19970228)16:4<385::aid-sim380>3.0.co;2-3

13. Nishimura Rick A., Otto Catherine M., Bonow Robert O., Carabello Blase A., Erwin John P., Guyton Robert A., O'Gara Patrick T., Ruiz Carlos E., Skubas Nikolaos J., Sorajja Paul, Sundt Thoralf M., Thomas James D. 2014 AHA/ACC Guideline for the Management of Patients With Valvular Heart Disease: Executive Summary. *Circulation*. 2014;129(23):2440-2492. doi:10.1161/CIR.0000000000000029

14. Sievers H-H, Schmidtke C. A classification system for the bicuspid aortic valve from 304 surgical specimens. *J Thorac Cardiovasc Surg*. 2007;133(5):1226-1233. doi:10.1016/j.jtcvs.2007.01.039

15. Singal AG, Mukherjee A, Elmunzer JB, Higgins PDR, Lok AS, Zhu J, Marrero JA, Waljee AK. Machine Learning Algorithms Outperform Conventional Regression Models in Predicting Development of Hepatocellular Carcinoma: *Am J Gastroenterol*.

For peer review only

2013;108(11):1723-1730. doi:10.1038/ajg.2013.332
16. Lambin P, van Stiphout RGPM, Starmans MHW, Rios-Velazquez E, Nalbantov G, Aerts HJWL, Roelofs E, van Elmpt W, Boutros PC, Granone P, Valentini V, Begg AC, De Ruysscher D, Dekker A. Predicting outcomes in radiation oncology—multifactorial decision support systems. *Nat Rev Clin Oncol*. 2013;10(1):27-40. doi:10.1038/nrclinonc.2012.196
17. Fern'andez-Delgado M, Cernadas E, Barro S, Amorim D. Do we need hundreds of classifiers to solve real world classification problems? *J Mach Learn Res*. 2014;15(1).
18. Jackson V, Petrini J, Eriksson MJ, Caidahl K, Eriksson P, Franco-Cereceda A. Aortic dimensions in relation to bicuspid and tricuspid aortic valve pathology. *J Heart Valve Dis*. 2014;23(4):463-472.
19. Takagi H, Umemoto T, for the ALICE (All-Literature Investigation of Cardiovascular Evidence) Group. Negative association of diabetes with rupture of abdominal aortic aneurysm. *Diab Vasc Dis Res*. 2016;13(5):341-347. doi:10.1177/1479164116651389
20. Yu X, Jiang D, Wang J, Wang R, Chen T, Wang K, Durgahee MSA, Wei X, Cao S. Metformin prescription and aortic aneurysm: systematic review and meta-analysis. *Heart*. 2019;105(17):1351-1357. doi:10.1136/heartjnl-2018-314639
21. Li L, Mamputu J-C, Wiernsperger N, Renier G. Signaling Pathways Involved in Human Vascular Smooth Muscle Cell Proliferation and Matrix Metalloproteinase-2 Expression Induced by Leptin: Inhibitory Effect of Metformin. *Diabetes*. 2005;54(7):2227-2234. doi:10.2337/diabetes.54.7.2227
22. Vainas T, Lubbers T, Stassen FRM, Herngreen SB, van Dieijen-Visser MP, Bruggeman CA, Kitslaar PJEHM, Schurink GWH. Serum C-Reactive Protein Level Is Associated With Abdominal Aortic Aneurysm Size and May Be Produced by Aneurysmal Tissue. *Circulation*. 2003;107(8):1103-1105. doi:10.1161/01.CIR.0000059938.95404.92
23. Shangwei Z, Yingqi W, Jiang X, Zhongyin W, Juan J, Dafang C, Yonghua H, Wei G. Serum High-Sensitive C-Reactive Protein Level and CRP Genetic Polymorphisms Are Associated with Abdominal Aortic Aneurysm. *Ann Vasc Surg*. 2017;45:186-192. doi:10.1016/j.avsg.2017.05.024
24. McNally A, Madan A, Sucosky P. Morphotype-Dependent Flow Characteristics in Bicuspid Aortic Valve Ascending Aortas: A Benchtop Particle Image Velocimetry Study. *Front Physiol*. 2017;8. doi:10.3389/fphys.2017.00044
25. Yang B, Zhou W, Jiao J, Nielsen JB, Mathis MR, Heydarpour M, Lettre G, Folkersen L, Prakash S, Schurmann C, Fritsche L, Farnum GA, Lin M, Othman M, Hornsby W, Driscoll A, Levasseur A, Thomas M, Farhat L, Dubé M-P, Isselbacher EM, Franco-Cereceda A, Guo D, Bottinger EP, Deeb GM, Booher A, Kheterpal S, Chen YE, Kang HM, Kitzman J, Cordell HJ, Keavney BD, Goodship JA, Ganesh SK, Abecasis G, Eagle KA, Boyle AP, Loos RJF, Eriksson P, Tardif J-C, Brummett CM, Milewicz DM, Body SC, Willer CJ. Protein-altering and regulatory genetic variants near GATA4 implicated in bicuspid aortic valve. *Nat Commun*. 2017;8(1):15481. doi:10.1038/ncomms15481
26. Baylor-Hopkins Center for Mendelian Genomics, MIBAVA Leducq Consortium, Gould RA, Aziz H, Woods CE, Seman-Senderos MA, Sparks E, Preuss C, Wünnemann F, Bedja D, Moats CR, McClymont SA, Rose R, Sobreira N, Ling H, MacCarrick G, Kumar AA, Luyckx I, Cannaerts E, Verstraeten A, Björk HM, Lehsau A-C, Jaskula-Ranga V, Lauridsen H, Shah AA, Bennett CL, Ellinor PT, Lin H, Isselbacher EM, Lino Cardenas CL, Butcher JT, Hughes GC, Lindsay ME, Mertens L, Franco-Cereceda A, Verhagen JMA, Wessels M, Mohamed SA, Eriksson P, Mital S, Van Laer L, Loeys BL, Andelfinger G, McCallion AS, Dietz HC. ROBO4 variants predispose individuals to bicuspid aortic valve and thoracic aortic aneurysm. *Nat Genet*. 2019;51(1):42-50. doi:10.1038/s41588-018-0265-y
27. Pasipoularides A. Clinical-pathological correlations of BAV and the attendant thoracic aortopathies. Part 2: Pluridisciplinary perspective on their genetic and molecular origins. *J*

For peer review only - http://bmjopen.bmj.com/site/about/guidelines.xhtml

Mol Cell Cardiol. 2019;133:233-246. doi:10.1016/j.yjmcc.2019.05.022
28. Cripe L, Andelfinger G, Martin LJ, Shooner K, Benson DW. Bicuspid aortic valve is heritable. *J Am Coll Cardiol*. 2004;44(1):138-143. doi:10.1016/j.jacc.2004.03.050
29. Georgakis MK, Gill D, Malik R, Protogerou AD, Webb AJS, Dichgans M. Genetically Predicted Blood Pressure Across the Lifespan: Differential Effects of Mean and Pulse Pressure on Stroke Risk. *Hypertension*. 2020;76(3):953-961. doi:10.1161/HYPERTENSIONAHA.120.15136
30. Wågsäter D, Paloschi V, Hanemaaijer R, Hultenby K, Bank RA, Franco-Cereceda A, Lindeman JHN, Eriksson P. Impaired Collagen Biosynthesis and Cross-linking in Aorta of Patients With Bicuspid Aortic Valve. *J Am Heart Assoc*. 2013;2(1). doi:10.1161/JAHA.112.000034
31. Forsell C, Björck HM, Eriksson P, Franco-Cereceda A, Gasser TC. Biomechanical Properties of the Thoracic Aneurysmal Wall: Differences Between Bicuspid Aortic Valve and Tricuspid Aortic Valve Patients. *Ann Thorac Surg*. 2014;98(1):65-71. doi:10.1016/j.athoracsur.2014.04.042
32. de Simone G, Roman MJ, De Marco M, Bella JN, Izzo R, Lee ET, Devereux RB. Hemodynamic Correlates of Abnormal Aortic Root Dimension in an Adult Population: The Strong Heart Study. *J Am Heart Assoc*. 2015;4(10). doi:10.1161/JAHA.115.002309
33. Schram MT, Kostense PJ, van Dijk RAJM, Dekker JM, Nijpels G, Bouter LM, Heine RJ, Stehouwer CDA. Diabetes, pulse pressure and cardiovascular mortality: the Hoorn Study: *J Hypertens*. 2002;20(9):1743-1751. doi:10.1097/00004872-200209000-00017
34. Grewal N, Gittenberger-de Groot AC, Poelmann RE, Klautz RJM, Lindeman JHN, Goumans M-J, Palmen M, Mohamed SA, Sievers H-H, Bogers AJJC, DeRuiter MC. Ascending aorta dilation in association with bicuspid aortic valve: A maturation defect of the aortic wall. *J Thorac Cardiovasc Surg*. 2014;148(4):1583-1590. doi:10.1016/j.jtcvs.2014.01.027
35. Della Corte A, Bancone C, Quarto C, Dialetto G, Covino FE, Scardone M, Caianiello G, Cotrufo M. Predictors of ascending aortic dilatation with bicuspid aortic valve: a wide spectrum of disease expression☆. *Eur J Cardiothorac Surg*. 2007;31(3):397-405. doi:10.1016/j.ejcts.2006.12.006
36. Baumgartner H, Falk V, Bax JJ, De Bonis M, Hamm C, Holm PJ, Iung B, Lancellotti P, Lansac E, Rodriguez Muñoz D, Rosenhek R, Sjögren J, Tornos Mas P, Vahanian A, Walther T, Wendler O, Windecker S, Zamorano JL, ESC Scientific Document Group, Roffi M, Alfieri O, Agewall S, Ahlsson A, Barbato E, Bueno H, Collet J-P, Coman IM, Czerny M, Delgado V, Fitzsimons D, Folliguet T, Gaemperli O, Habib G, Harringer W, Haude M, Hindricks G, Katus HA, Knuuti J, Kolh P, Leclercq C, McDonagh TA, Piepoli MF, Pierard LA, Ponikowski P, Rosano GMC, Ruschitzka F, Shlyakhto E, Simpson IA, Sousa-Uva M, Stepinska J, Tarantini G, Tchétché D, Aboyans V, Windecker S, Aboyans V, Agewall S, Barbato E, Bueno H, Coca A, Collet J-P, Coman IM, Dean V, Delgado V, Fitzsimons D, Gaemperli O, Hindricks G, Iung B, Jüni P, Katus HA, Knuuti J, Lancellotti P, Leclercq C, McDonagh T, Piepoli MF, Ponikowski P, Richter DJ, Roffi M, Shlyakhto E, Simpson IA, Zamorano JL, Kzhdryan HK, Mascherbauer J, Samadov F, Shumavets V, Camp GV, Lončar D, Lovric D, Georgiou GM, Linhartova K, Ihlemann N, Abdelhamid M, Pern T, Turpeinen A, Srbinovska-Kostovska E, Cohen A, Bakhutashvili Z, Ince H, Vavuranakis M, et al. 2017 ESC/EACTS Guidelines for the management of valvular heart disease. *Eur Heart J*. 2017;38(36):2739-2791. doi:10.1093/eurheartj/ehx391
37. Schaefer BM, Lewin MB, Stout KK, Gill E, Prueitt A, Byers PH, Otto CM. The bicuspid aortic valve: an integrated phenotypic classification of leaflet morphology and aortic root shape. *Heart*. 2008;94(12):1634-1638. doi:10.1136/hrt.2007.132092

For peer review only - http://bmjopen.bmj.com/site/about/guidelines.xhtml

Figure 1. Principal component analysis (PCA) plot of BAV versus TAV patients (with or without aneurysm).

A, BAV patients

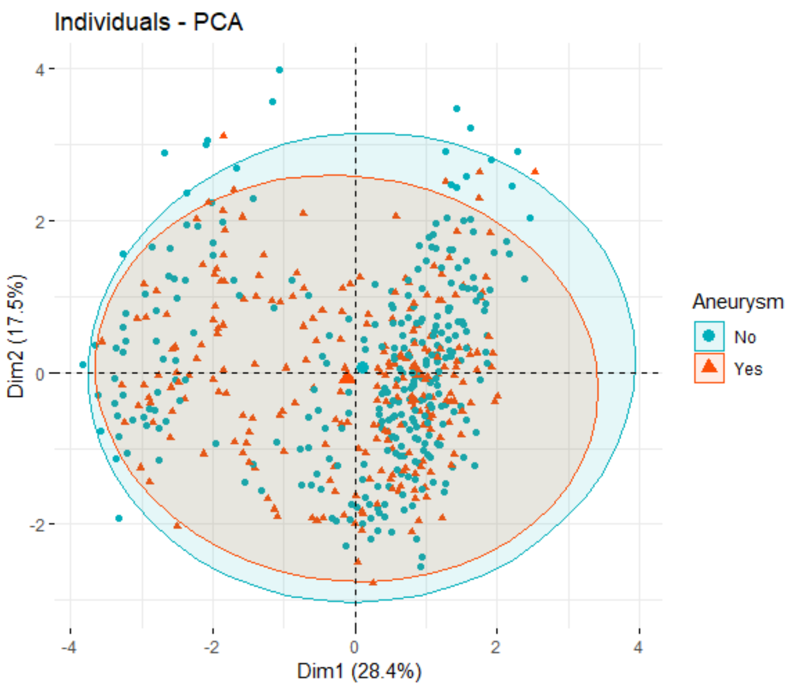

B, TAV patients

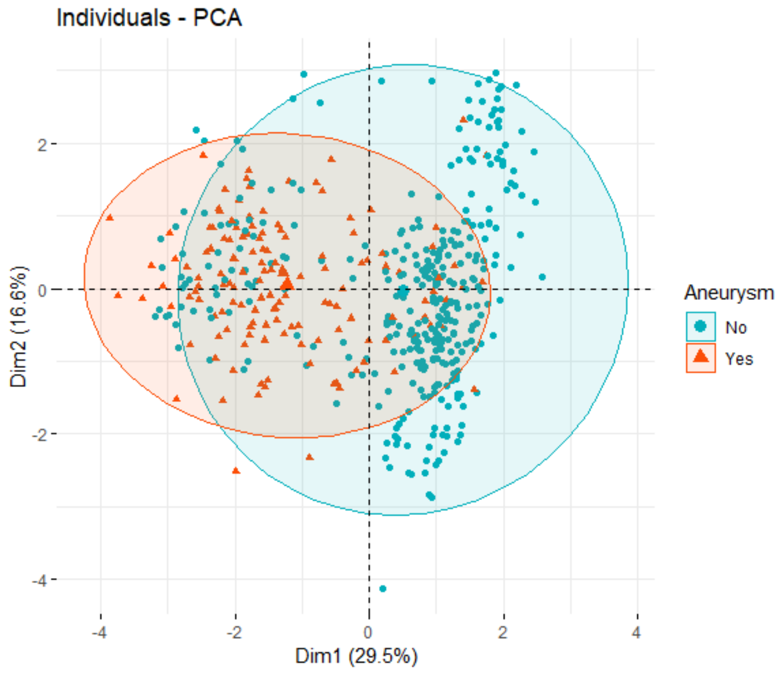

Figure 2. The ROC curve for risk prediction model across the predictive methods used.

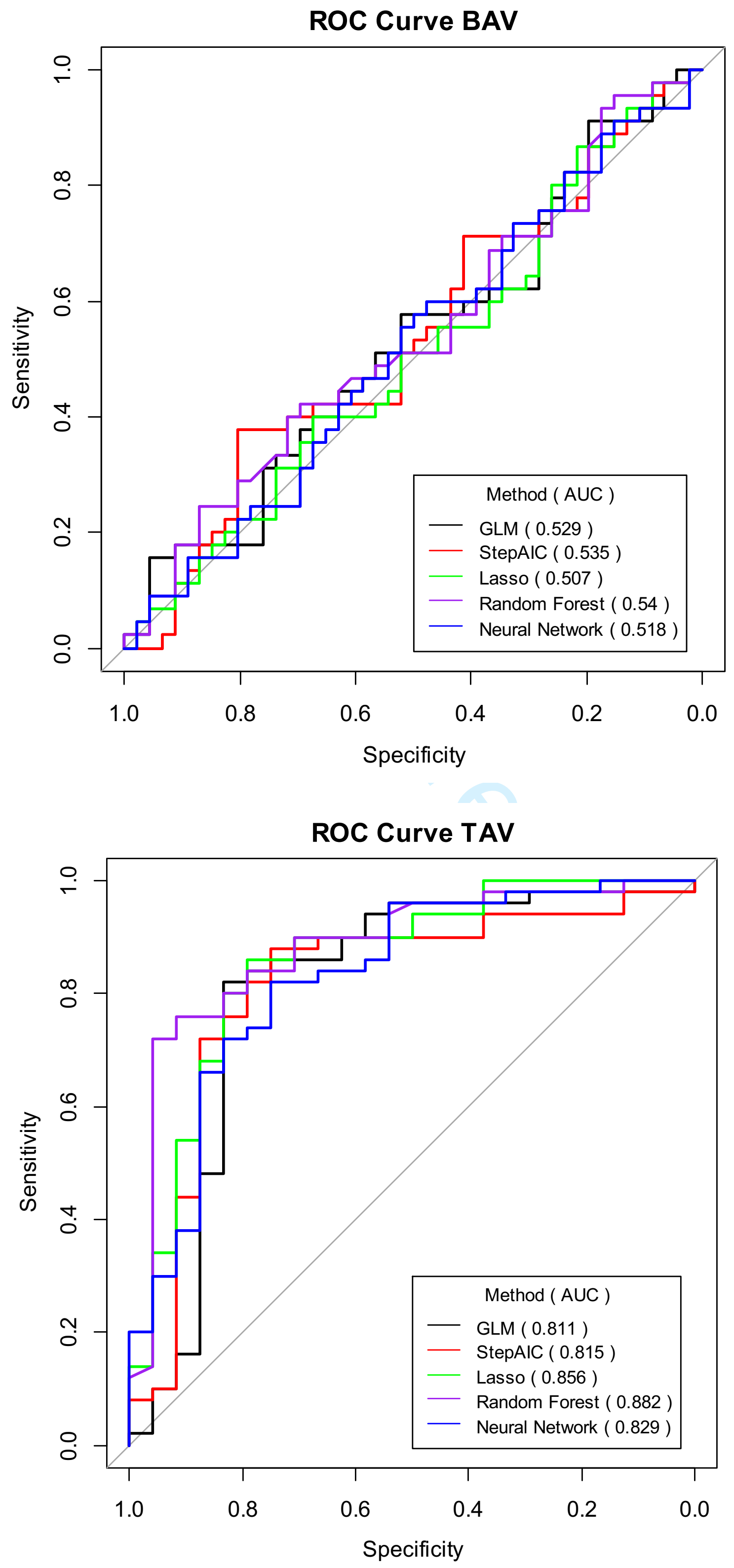

Clinical classifiers to identify ascending aortic dilatation in patients with bicuspid versus tricuspid aortic valves

Supplementary Appendix Table of Contents:

Tables

e-Table 1: Characteristics of Patients with BAV versus TAV.
e-Table 2. Additional cardiovascular drugs including anti-hypertensive therapy.
e-Table 3. Performance of the algorithms.
e-Table 4: Predictors of ascending aortic dilatation in BAV and TAV patients, separately. Univariate and Multivariate logistic regression analysis: Imputed data.
e-Table 5: Baseline characteristics of included and excluded study participants.
e-Table 6: Contributors to the prediction of aortic dilatation in TAV.

Figures

e-Figure 1: Flowchart of the study
e-Figure 2: Plot of methodology used to choose variables.
e-Figure 3: The ROC curve for our risk prediction model across the predictive methods using imputed data.
e-Figure 4. Principal component analysis (PCA) plot of TAV patients with or without aneurysm stratified by AS
e-Figure 5. The ROC curve for our risk prediction model across the predictive methods without AS as predictor;

Dataset selection:

In order to gather maximum of information from the available measurement in the ASAP/DAVAACA cohort, we first created several blocks of meaningful variables: clinical data, family history of cardiovascular diseases, prevalent diseases, demographic characteristics, lifestyle habits data, blood sample data and medication. Then to select the most meaningful variables by avoiding collinearity within each bloc, we performed several analyses: (1) a hierarchical clustering, then (2) we ran Principal Component Analysis, (3) we lastly created the correlation matrix inside each cluster (and its correlation plot) (**Figure e-2**).

For peer review only

e-Table 1: Characteristics of Patients with BAV versus TAV

	BAV N=543	TAV N=491	P value
**Gender: Male**	397 (73.1%)	311 (63.3%)	0.001
**Age**	60.4 (12.4)	70.4 (9.09)	<0.001
**Height**	175 (9.30)	172 (9.80)	<0.001
**Weight**	81.9 (15.8)	81.5 (15.9)	0.697
**BSA**	1.98 (0.22)	1.94 (0.22)	0.012
**BMI**	26.6 (4.26)	27.4 (4.77)	0.002
**Regular Smoker:**			0.023
**No**	250 (46.6%)	213 (43.6%)	
**Former**	234 (43.7%)	246 (50.4%)	
**Yes**	52 (9.70%)	29 (5.94%)	
**Systolic Blood Pressure**	136 (18.6)	142 (20.4)	<0.001
**Diastolic Blood Pressure**	80.1 (11.9)	76.1 (13.2)	<0.001
**PP**	55.4 (17.6)	66.4 (19.7)	<0.001
**Leukocytes**	5.80 [4.80;6.90]	6.00 [5.03;7.40]	0.004
**hsCRP**	1.10 [0.55;2.50]	1.80 [0.79;3.80]	<0.001
**Cholesterol**	4.80 [4.20;5.60]	4.60 [3.90;5.60]	0.016
**LDL**	2.80 [2.20;3.60]	2.60 [2.00;3.50]	0.009
**Aortic Stenosis:**	404 (74.4%)	306 (62.3%)	<0.001
**Aortic Insufficiency:**	158 (29.1%)	160 (32.6%)	0.252
**Diameter of Aortic Anulus**	24.8 (3.57)	23.0 (3.08)	<0.001
**Diameter of Valsalva Sinus**	35.6 (5.68)	34.9 (6.94)	0.056
**Diameter of Sinotubular Junction**	30.5 (5.70)	29.0 (6.49)	<0.001
**Diameter of Ascending Aorta**	39.3 (8.29)	37.1 (10.1)	<0.001
**Dilatation of Ascending Aorta:**	297 (54.7%)	349 (71.1%)	<0.001
**Sibling MI history before 65 year**	32 (6.24%)	46 (10.0%)	0.041
**Mother MI history before 65 year**	25 (4.84%)	32 (6.97%)	0.199
**Father MI history before 65 year**	69 (13.7%)	63 (14.3%)	0.874
**Myocardial Infarction**	19 (3.53%)	42 (8.59%)	0.001
**Stroke**	30 (5.55%)	57 (11.7%)	0.001
**Abdominal Aorta Aneurysm**	25 (4.63%)	30 (6.19%)	0.335
**Angina Pectoris**	35 (6.49%)	61 (12.5%)	0.001
**Heart Failure**	40 (7.50%)	57 (11.7%)	0.029
**Hypertension**	255 (47.4%)	311 (64.4%)	<0.001
**Diabetes**	54 (10.0%)	64 (13.1%)	0.139
**Anti-hypertensive drugs**			
**ACE inhibitor**	103 (19.0%)	142 (29.0%)	<0.001
**Aspirin**	132 (24.4%)	193 (39.6%)	<0.001
**Betablocker**	217 (40.1%)	255 (52.0%)	<0.001

For peer review only

e-Table 2. Additional cardiovascular drugs including anti-hypertensive therapy.

	BAV patients (n=543)			TAV patients (n=491)		
	**Dilated (n=246)**	**Non-Dilated (n=297)**	**P value**	**Dilated (n=142)**	**Non-Dilated (n=349)**	**P value**
**ACE inhibitor**	46 (18.7%)	57 (19.3%)	0.941	45 (31.9%)	97 (27.9%)	0.434
**Angiotensin II blocker**	42 (17.1%)	45 (15.3%)	0.648	38 (27.0%)	78 (22.5%)	0.350
**Beta blocker**	92 (37.4%)	125 (42.4%)	0.277	76 (53.9%)	179 (51.3%)	0.672
**Calcium antagonist**	40 (16.3%)	41 (13.9%)	0.518	40 (28.4%)	87 (24.9%)	0.501
**Loop diuretics**	22 (8.94%)	35 (11.9%)	0.330	19 (13.5%)	65 (18.6%)	0.216
**Thiazide diuretics***	1 (0.81%)	1 (0.72%)	0.931	2 (2.99%)	5 (3.70%)	0.793
**Lipid lowering agent**	79 (32.1%)	107 (36.3%)	0.356	45 (32.1%)	193 (55.3%)	<0.001
**Anticoagulant**	28 (11.4%)	26 (8.81%)	0.396	12 (8.51%)	50 (14.4%)	0.107
**Insulin**	4 (1.63%)	13 (4.41%)	0.112	0 (0.00%)	17 (4.87%)	0.005
**Peroral antidiabetics***	4 (3.25%)	5 (3.60%)	0.878	2 (2.99%)	16 (11.9%)	0.069

*Results concerning these variables should be precocious due to high rate of missing value (n=464).

For peer review only

e-Table 3. Performance of the algorithms

Method	Accuracy	AUC	Sensitivity	Specificity	Positive Predictive Value	Negative Predictive Value
			BAV			
**Method**	**Accuracy**	**AUC**	**Sensitivity**	**Specificity**	**Positive Predictive Value**	**Negative Predictive Value**
**GLM**	0.528	0.529	0.444	0.609	0.526	0.528
**StepAIC**	0.506	0.535	0.422	0.587	0.500	0.509
**Lasso**	0.484	0.507	0.422	0.544	0.475	0.490
**Random Forest**	0.517	0.540	0.467	0.565	0.512	0.520
**Neural Network**	0.473	0.518	0.489	0.457	0.468	0.477
			TAV			
**Method**	**Accuracy**	**AUC**	**Sensitivity**	**Specificity**	**Positive Predictive Value**	**Negative Predictive Value**
**GLM**	0.824	0.811	0.820	0.833	0.911	0.690
**StepAIC**	0.824	0.815	0.900	0.667	0.849	0.762
**Lasso**	0.824	0.854	0.860	0.750	0.878	0.720
**Random Forest**	0.797	0.882	0.840	0.708	0.857	0.680
**Neural Network**	0.770	0.825	0.820	0.667	0.837	0.640
			BAV (Imputed data)			
**Method**	**Accuracy**	**AUC**	**Sensitivity**	**Specificity**	**Positive Predictive Value**	**Negative Predictive Value**
**GLM**	0.570	0.586	0.689	0.426	0.593	0.531
**StepAIC**	0.563	0.526	0.676	0.426	0.588	0.520
**Lasso**	0.578	0.593	0.676	0.459	0.602	0.539
**Random Forest**	0.556	0.556	0.662	0.426	0.583	0.548
**Neural Network**	0.541	0.540	0.581	0.492	0.581	0.492
			TAV (Imputed data)			
**Method**	**Accuracy**	**AUC**	**Sensitivity**	**Specificity**	**Positive Predictive Value**	**Negative Predictive Value**
**GLM**	0.820	0.831	0.816	0.829	0.922	0.644
**StepAIC**	0.803	0.838	0.816	0.771	0.899	0.628
**Lasso**	0.812	0.842	0.793	0.857	0.932	0.625
**Random Forest**	0.820	0.857	0.828	0.800	0.911	0.651
**Neural Network**	0.812	0.799	0.862	0.686	0.872	0.667

For peer review only

e-Table 4. Predictors of aortopathy in BAV and TAV patients separately, Univariate and Multivariate logistic regression analysis: Imputed data

	**BAV imputed (N= 543)**						**TAV imputed (n= 491)**					
	unAdjusted Model		***Model 1***		***Model 2***		***unAdjusted Model***		***Model 1***		***Model 2***	
	OR (95% IC)*	***p***	***OR (95% IC)****	***p***	***OR (95% IC)****	***p***	***OR (95% IC)****	***p***	***OR (95% IC)****	***p***	***OR (95% IC)****	***p***
**Aortic Stenosis**	0.58 (0.39-0.85)	0.006	0.43 (0.27-0.67)	0.002	0.42 (0.22-0.79)	0.007	0.05 (0.03-0.07)	<0.001	0.04 (0.02-0.07)	<0.001	0.02 (0.01-0.05)	<0.001
**Aortic Regurgitation**	1.51 (1.04-2.19)	0.031	1.89 (1.24-2.88)	0.003	1.35 (0.75-2.44)	0.316	7.99 (5.19-12.45)	<0.001	7.27 (4.56-11.77)	<0.001	0.51 (0.20-1.18)	0.133
**Pulse Pressure**	0.99 (0.98-1.00)	0.009	0.99 (0.98-1.00)	0.013	0.98 (0.97-0.99)	<0.001	1.01 (1.00-1.02)	0.088	1.01 (1.00-1.02)	0.163	0.99 (0.98-1.00)	0.132
**Diabetes**	0.42 (0.22-0.76)	0.006	0.34 (0.18-0.65)	0.001	0.39 (0.19-0.74)	0.005	0.10 (0.02-028)	<0.001	0.10 (0.02-0.29)	<0.001	0.21 (0.05-0.71)	0.022

* Odds ratio and 95% confidence interval limits were obtained by logistic regression and were adjusted for age, sex, body surface area (BSA), low-density Lipoprotein (LDL), high-sensitivity C-reactive protein (hsCRP) and Diabetes

For peer review only

e-Table 5. Baseline characteristics of included and excluded study participants

	[ALL] N=1180	Data N=1041	NA N=139	p.overall	N
**Gender: Male**	807 (68.4%)	708 (68.5%)	99 (67.8%)	0.947	1180
**Age**	64.9 (12.4)	65.2 (12.0)	62.9 (14.5)	0.077	1180
**Height**	174 (9.60)	174 (9.65)	175 (9.15)	0.263	1171
**Weight**	81.8 (15.6)	81.7 (15.8)	82.6 (14.4)	0.514	1168
**BSA**	1.96 (0.21)	1.96 (0.22)	1.98 (0.19)	0.305	1141
**BMI**	27.0 (4.56)	27.0 (4.53)	27.1 (4.80)	0.746	1167
**Regular Smoker:**				0.549	1168
**No**	535 (45.8%)	463 (45.2%)	72 (50.0%)		
**Former**	541 (46.3%)	480 (46.9%)	61 (42.4%)		
**Yes**	92 (7.88%)	81 (7.91%)	11 (7.64%)		
**Cusps:**				0.951	1140
**BAV**	599 (52.5%)	543 (52.5%)	56 (52.8%)		
**TAV**	541 (47.5%)	491 (47.5%)	50 (47.2%)		
**Raphe:**				.	1158
**True BAV**	50 (4.32%)	47 (4.60%)	3 (2.19%)		
**Left-Non coronary**	6 (0.52%)	5 (0.49%)	1 (0.73%)		
**Right-Non coronary**	99 (8.55%)	90 (8.81%)	9 (6.57%)		
**Right-Left**	432 (37.3%)	388 (38.0%)	44 (32.1%)		
**Systolic Blood Pressure**	138 (19.7)	139 (19.8)	136 (18.8)	0.053	1172
**Diastolic Blood Pressure**	78.3 (12.7)	78.2 (12.7)	79.3 (12.7)	0.305	1171
**PP**	60.1 (19.2)	60.6 (19.4)	56.2 (17.2)	0.005	1171
**Leukocytes**	5.90 [4.90;7.10]	5.90 [4.90;7.10]	5.90 [4.90;6.90]	0.665	1168
**hsCRP**	1.40 [0.64;3.20]	1.40 [0.63;3.10]	1.60 [0.72;3.52]	0.153	1159
**Cholesterol**	4.80 [4.00;5.68]	4.80 [4.00;5.60]	5.10 [4.30;6.10]	0.004	1162
**LDL**	2.80 [2.20;3.60]	2.80 [2.10;3.50]	3.20 [2.30;3.90]	0.002	1146
**Aortic Stenosis:**	813 (69.1%)	710 (68.7%)	103 (72.0%)	0.472	1177
**Aortic Insufficiency:**	361 (30.7%)	318 (30.8%)	43 (30.1%)	0.944	1177
**Diameter of Aortic Anulus**	23.9 (3.40)	24.0 (3.46)	23.5 (2.87)	0.148	1154
**Diameter of Valsalva Sinus**	35.4 (6.27)	35.3 (6.32)	36.2 (5.83)	0.095	1157
**Diameter of Sinotubular Junction**	29.9 (6.05)	29.8 (6.13)	30.7 (5.34)	0.083	1112
**Diameter of Ascending Aorta**	38.3 (9.24)	38.2 (9.28)	38.8 (8.09)	0.686	1069
**Dilatation of Ascending Aorta:**	402 (37.6%)	388 (37.5%)	14 (40.0%)	0.904	1069
**Sibling MI history before 65-y**	86 (7.76%)	78 (8.02%)	8 (5.93%)	0.497	1108
**Mother MI history before 65-y**	63 (5.65%)	57 (5.84%)	6 (4.29%)	0.583	1116
**Father MI history before 65-y**	151 (14.0%)	132 (14.0%)	19 (14.2%)	0.959	1076
**Myocardial Infarction**	66 (5.64%)	61 (5.94%)	5 (3.47%)	0.313	1171
**Stroke**	99 (8.43%)	87 (8.45%)	12 (8.33%)	0.964	1174
**Abdominal Aorta Aneurysm**	58 (4.96%)	55 (5.37%)	3 (2.07%)	0.132	1170
**Angina Pectoris**	103 (8.80%)	96 (9.35%)	7 (4.86%)	0.105	1171
**Heart Failure**	112 (9.64%)	97 (9.52%)	15 (10.5%)	0.828	1162
**Hypertension**	635 (54.5%)	566 (55.4%)	69 (47.6%)	0.092	1166
**Diabetes**	136 (11.6%)	118 (11.5%)	18 (12.4%)	0.852	1172
**Anti-hypertensive drugs**					
**ACE inhibitor:**	277 (23.6%)	245 (23.8%)	32 (22.4%)	0.790	1173
**Aspirin:**	352 (30.1%)	325 (31.6%)	27 (18.9%)	0.003	1171
**Betablocker:**	530 (45.1%)	472 (45.8%)	58 (40.6%)	0.277	1174

For peer review only

e-Table 6. Contributors to the predictive classifiers in TAV

GLM		StepAIC (top 10)		Lasso (top 10)		RF (top 10)		Neural network (top 10)	
**AS**	100.000	**AS**	100.000	**AS**	100.000	**AS**	100.000	**AS**	100.00
**age**	27.762	**AI**	70.043	**Diabetes**	39.6265	**AI**	23.581	**age**	62.81
**Diabetes**	27.003	**age**	41.852	**Renal Failure**	26.2556	**Creatinine**	21.571	**Diabetes**	47.28
**AI**	21.226	**LDL**	31.027	**Father Stroke o r Mi or TIA65**	16.4437	**age**	21.181	**Beta blocker**	41.58
**BSA**	17.970	**Hemoglobin**	27.641	**MI**	15.3655	**PP**	16.418	**Creatinine**	38.18
**Pulse BP**	16.595	**ASA**	26.802	**ALAT**	14.7816	**Leukocytes**	14.558	**FatherMI65**	35.95
**LDL**	8.753	**Cholesterol**	26.657	**Sibling AP65**	14.7506	**hsCRP**	13.549	**Diastolic BP**	34.47
**Gender**	7.564	**Diabetes**	26.541	**HF**	12.8772	**Systolic BP**	13.540	**Deep VTPEM**	33.27
**hsCRP**	0.000	**hsCRP**	22.648	**Father MI65**	12.4264	**Height**	13.199	**Father Stroke or Mi or TIA65**	33.16

For peer review only

e-Figure 1: Flowchart of the study

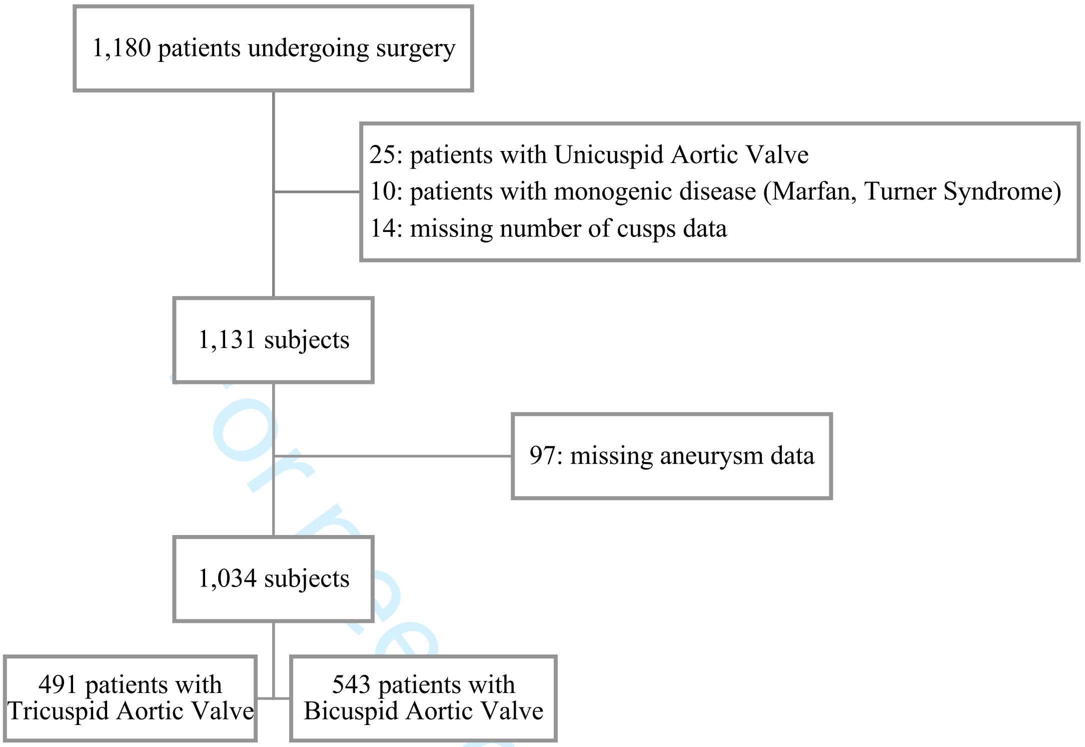

For peer review only

e-Figure 2. Plot of methodology used to choose variables

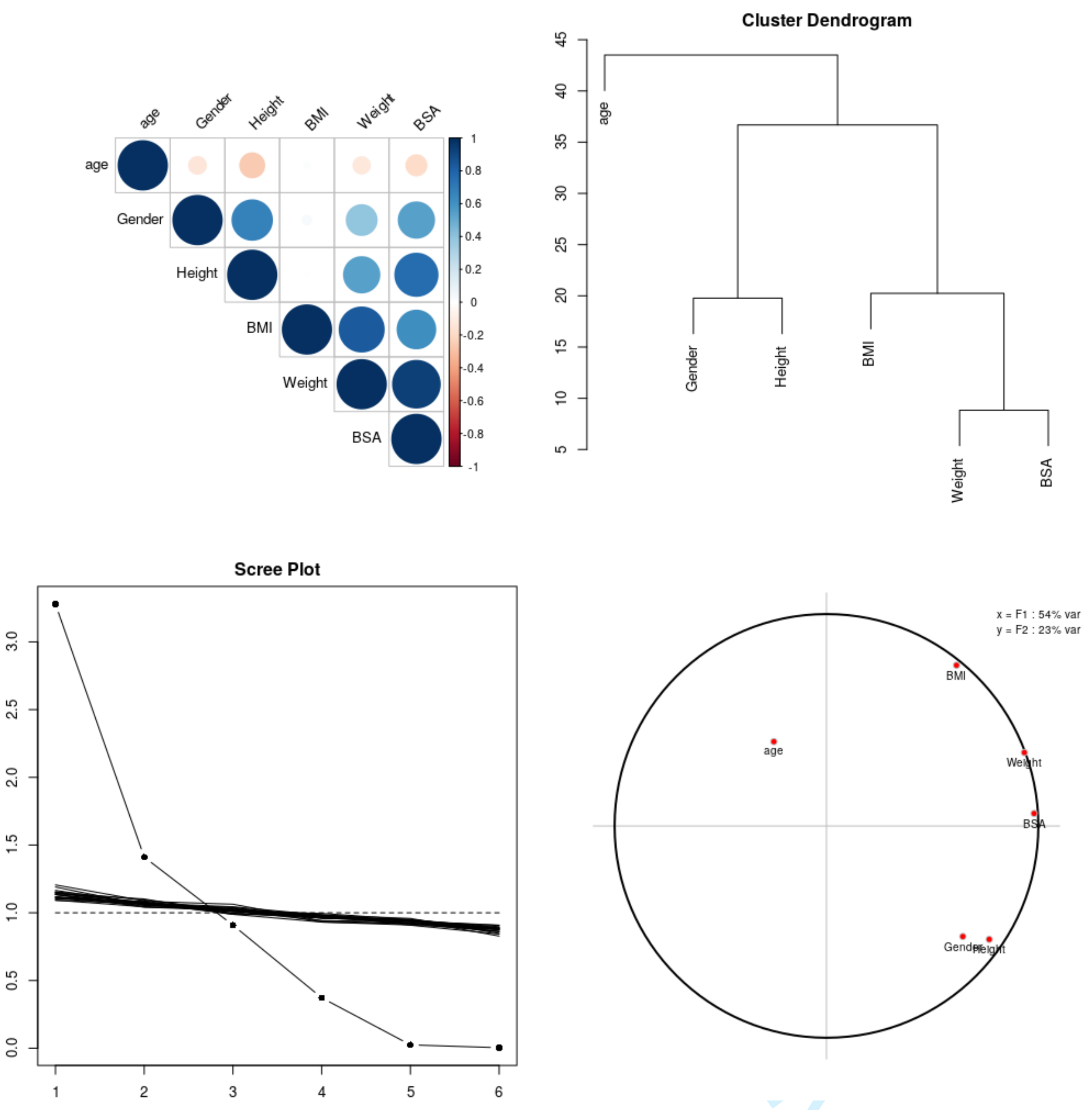

e-Figure 3. The ROC curve for our risk prediction model across the predictive methods using imputed data

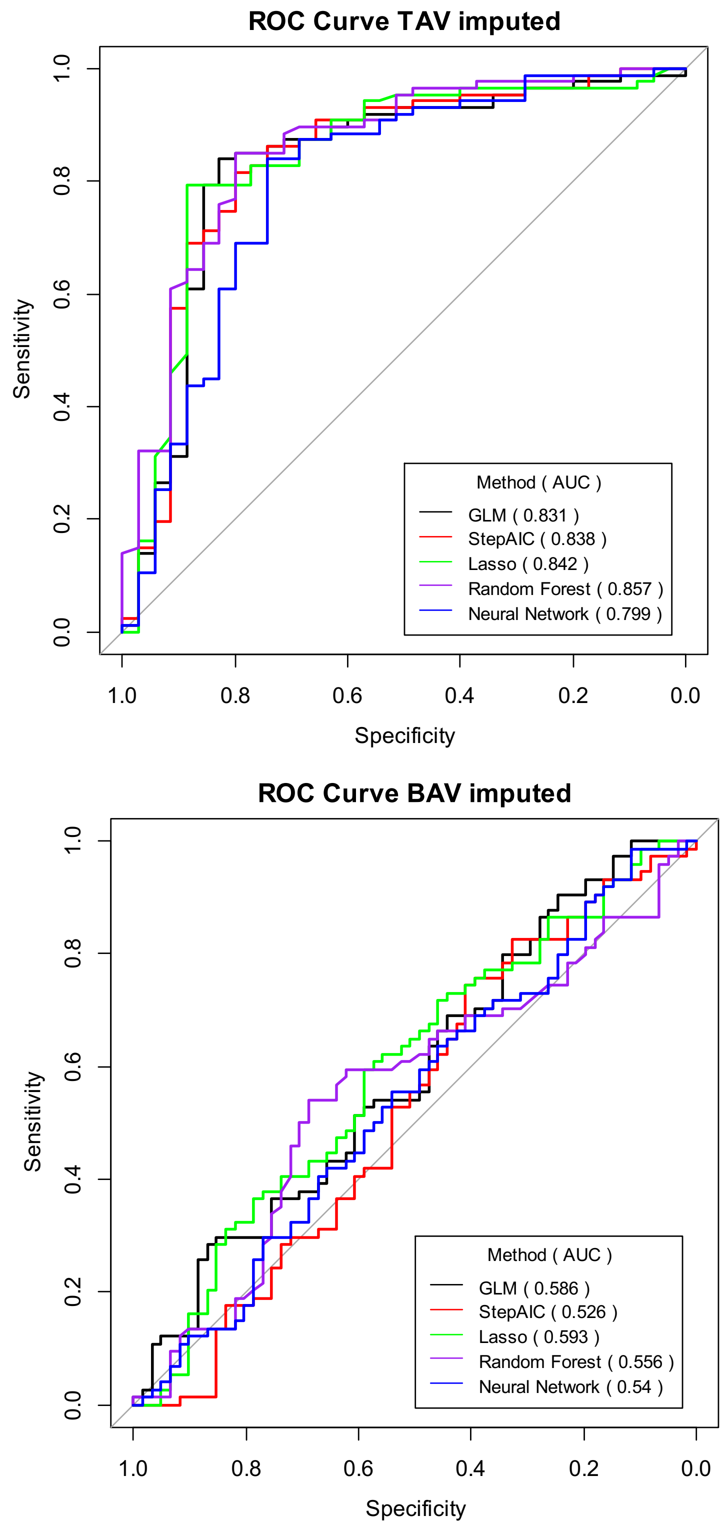

e-Figure 4. Principal component analysis (PCA) plot of TAV patients with or without aneurysm stratified by AS

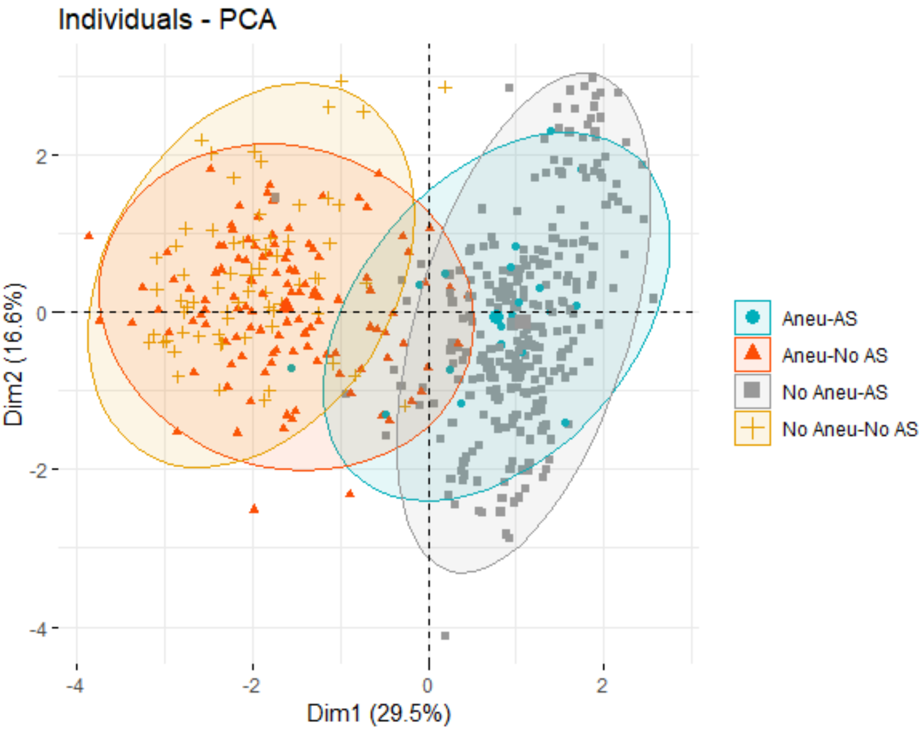

eview only

e-Figure 5. The ROC curve for our risk prediction model across the predictive methods without AS as predictor

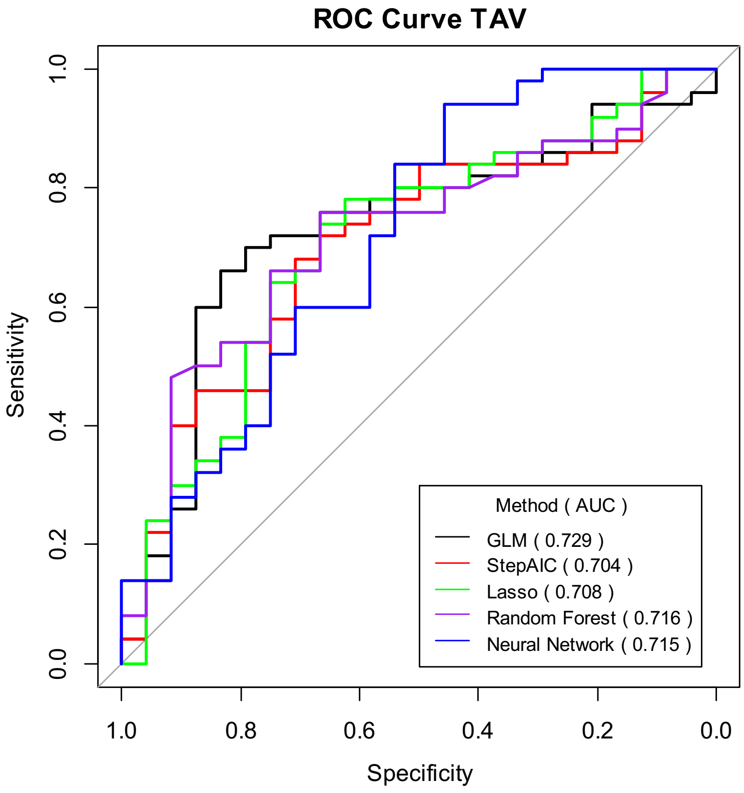

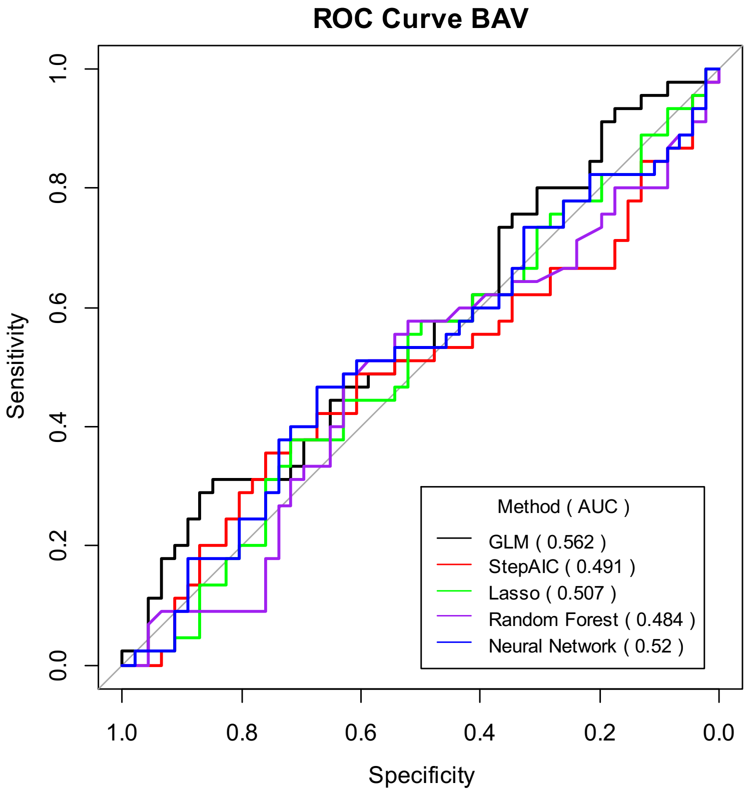

STROBE Statement—Checklist of items that should be included in reports of ***cohort studies***

	Item No	Recommendation	Page No
**Title and abstract**	1	(*a*) Indicate the study's design with a commonly used term in the title or the abstract	1
		(*b*) Provide in the abstract an informative and balanced summary of what was done and what was found	2
**Introduction**			
Background/rationale	2	Explain the scientific background and rationale for the investigation being reported	4
Objectives	3	State specific objectives, including any prespecified hypotheses	4
**Methods**			
Study design	4	Present key elements of study design early in the paper	5
Setting	5	Describe the setting, locations, and relevant dates, including periods of recruitment, exposure, follow-up, and data collection	5
Participants	6	(*a*) Give the eligibility criteria, and the sources and methods of selection of participants. Describe methods of follow-up	5
		(*b*) For matched studies, give matching criteria and number of exposed and unexposed	
Variables	7	Clearly define all outcomes, exposures, predictors, potential confounders, and effect modifiers. Give diagnostic criteria, if applicable	5, 6, 7,
Data sources/ measurement	8*	For each variable of interest, give sources of data and details of methods of assessment (measurement). Describe comparability of assessment methods if there is more than one group	5, 6, 7
Bias	9	Describe any efforts to address potential sources of bias	5, 6
Study size	10	Explain how the study size was arrived at	e-fig 1
Quantitative variables	11	Explain how quantitative variables were handled in the analyses. If applicable, describe which groupings were chosen and why	5, 6, 7
Statistical methods	12	(*a*) Describe all statistical methods, including those used to control for confounding	5, 6, 7
		(*b*) Describe any methods used to examine subgroups and interactions	5, 6, 7
		(*c*) Explain how missing data were addressed	
		(*d*) If applicable, explain how loss to follow-up was addressed	
		(*e*) Describe any sensitivity analyses	7, 8, 13
**Results**			
Participants	13*	(a) Report numbers of individuals at each stage of study—eg numbers potentially eligible, examined for eligibility, confirmed eligible, included in the study, completing follow-up, and analysed	8, 9
		(b) Give reasons for non-participation at each stage	
		(c) Consider use of a flow diagram	
Descriptive data	14*	(a) Give characteristics of study participants (eg demographic, clinical, social) and information on exposures and potential confounders	8, 9, 10
		(b) Indicate number of participants with missing data for each variable of interest	9
		(c) Summarise follow-up time (eg, average and total amount)	
Outcome data	15*	Report numbers of outcome events or summary measures over time	

For peer review only

Main results	16	(*a*) Give unadjusted estimates and, if applicable, confounder-adjusted estimates and their precision (eg, 95% confidence interval). Make clear which confounders were adjusted for and why they were included<br>(*b*) Report category boundaries when continuous variables were categorized<br>(*c*) If relevant, consider translating estimates of relative risk into absolute risk for a meaningful time period	11, 12, 13
Other analyses	17	Report other analyses done—eg analyses of subgroups and interactions, and sensitivity analyses	
**Discussion**			
Key results	18	Summarise key results with reference to study objectives	13, 14
Limitations	19	Discuss limitations of the study, taking into account sources of potential bias or imprecision. Discuss both direction and magnitude of any potential bias	14, 15, 19
Interpretation	20	Give a cautious overall interpretation of results considering objectives, limitations, multiplicity of analyses, results from similar studies, and other relevant evidence	15, 16, 17
Generalisability	21	Discuss the generalisability (external validity) of the study results	17, 18
**Other information**			
Funding	22	Give the source of funding and the role of the funders for the present study and, if applicable, for the original study on which the present article is based	19

*Give information separately for exposed and unexposed groups.

Note: An Explanation and Elaboration article discusses each checklist item and gives methodological background and published examples of transparent reporting. The STROBE checklist is best used in conjunction with this article (freely available on the Web sites of PLoS Medicine at http://www.plosmedicine.org/, Annals of Internal Medicine at http://www.annals.org/, and Epidemiology at http://www.epidem.com/). Information on the STROBE Initiative is available at http://www.strobe-statement.org.

For peer review only

BMJ Open

Predictive Machine Learning Models for Ascending Aortic Dilatation in Patients with Bicuspid and Tricuspid Aortic Valves Undergoing Cardiothoracic Surgery: A prospective, single-center and observational study

Journal:	*BMJ Open*
Manuscript ID	bmjopen-2022-067977.R2
Article Type:	Original research
Date Submitted by the Author:	26-Jan-2024

Complete List of Authors:	Gaye, Bamba; INSERM U970, ; Universite de Paris Faculte de Sante,<br>Vignac, Maxime; Karolinska Institutet, Cardiovascular Medicine Unit, Center for Molecular Medicine, Department of Medicine<br>Gådin, Jesper R.; Karolinska Institutet, Cardiovascular Medicine Unit, Center for Molecular Medicine, Department of Medicine<br>Ladouceur, Magalie; INSERM; Georges Pompidou European Hospital Anatomical Pathology<br>Caidahl, Kenneth; Karolinska Institutet, Clinical Physiology Unit, Department of Molecular Medicine and Surgery<br>Olsson, Christian; Karolinska Institutet, Cardiothoracic Surgery Unit, Department of Molecular Medicine and Surgery<br>Franco-Cereceda, Anders; Karolinska Institutet, Cardiothoracic Surgery Unit, Department of Molecular Medicine and Surgery<br>Eriksson, Per ; Karolinska Institutet, Cardiovascular Medicine Unit, Center for Molecular Medicine, Department of Medicine<br>Björck, Hanna; Karolinska Institutet, Cardiovascular Medicine Unit, Center for Molecular Medicine, Department of Medicine

**Primary Subject Heading**:	Cardiovascular medicine
Secondary Subject Heading:	Surgery, Cardiovascular medicine
Keywords:	Valvular heart disease < CARDIOLOGY, EPIDEMIOLOGY, Cardiology < INTERNAL MEDICINE

SCHOLARONE™
Manuscripts

I, the Submitting Author has the right to grant and does grant on behalf of all authors of the Work (as defined in the below author licence), an exclusive licence and/or a non-exclusive licence for contributions from authors who are: i) UK Crown employees; ii) where BMJ has agreed a CC-BY licence shall apply, and/or iii) in accordance with the terms applicable for US Federal Government officers or employees acting as part of their official duties; on a worldwide, perpetual, irrevocable, royalty-free basis to BMJ Publishing Group Ltd ("BMJ") its licensees and where the relevant Journal is co-owned by BMJ to the co-owners of the Journal, to publish the Work in this journal and any other BMJ products and to exploit all rights, as set out in our licence.

The Submitting Author accepts and understands that any supply made under these terms is made by BMJ to the Submitting Author unless you are acting as an employee on behalf of your employer or a postgraduate student of an affiliated institution which is paying any applicable article publishing charge ("APC") for Open Access articles. Where the Submitting Author wishes to make the Work available on an Open Access basis (and intends to pay the relevant APC), the terms of reuse of such Open Access shall be governed by a Creative Commons licence – details of these licences and which Creative Commons licence will apply to this Work are set out in our licence referred to above.

Other than as permitted in any relevant BMJ Author's Self Archiving Policies, I confirm this Work has not been accepted for publication elsewhere, is not being considered for publication elsewhere and does not duplicate material already published. I confirm all authors consent to publication of this Work and authorise the granting of this licence.

For peer review only

Predictive Machine Learning Models for Ascending Aortic Dilatation in Patients with Bicuspid and Tricuspid Aortic Valves Undergoing Cardiothoracic Surgery: A prospective, single-center and observational study

Bamba Gaye[1*] (PhD), Maxime Vignac[1] (MSc), Jesper R. Gådin[1] (PhD), Magalie Ladouceur[2,3] (MD), Kenneth Caidahl[4] (MD, PhD), Christian Olsson[5] (MD, PhD), Anders Franco-Cereceda[5] (MD), Per Eriksson[1] (PhD), Hanna M. Björck[1*] (PhD)

Running Title: Clinical Classifiers in BAV versus TAV

Total Word Count: 3315

Affiliations:
[1]Cardiovascular Medicine Unit, Center for Molecular Medicine, Department of Medicine, Karolinska Institutet, Stockholm, Karolinska University Hospital, Solna, Sweden
[2]INSERM, U970, Paris Cardiovascular Research Center / Université Paris Descartes, Sorbonne Paris Cité, Faculté de Médecine, Paris, France
[3]AP-HP, Georges Pompidou European Hospital, Cardiology Department, Paris, France
[4]Clinical Physiology Unit, Department of Molecular Medicine and Surgery, Karolinska Institutet, Stockholm, Sweden
[5]Cardiothoracic Surgery Unit, Department of Molecular Medicine and Surgery, Karolinska Institutet, Stockholm, Sweden

Disclosure: All authors have nothing to disclose.

***Address for Correspondence:**
Dr. Bamba Gaye (email: bamba.gaye@inserm.fr)
Dr. Hanna Bjorck (email: hanna.bjorck@ki.se)
Cardiovascular Medicine Unit, BioClinicum J8:20, Visionsgatan 4, Karolinska University Hospital, SE-171 64 Solna, Sweden

For peer review only

Abstract

Objectives: The objective of this study was to develop clinical classifiers aiming to identify prevalent ascending aortic dilatation in patients with Bicuspid Aortic Valve (BAV) and tricuspid aortic valve (TAV).

Design and Setting: A prospective, single-center and observational cohort.

Participants: The study involved 543 BAV and 491 TAV patients with aortic valve disease and/or ascending aortic dilatation, excluding those with coronary artery disease, undergoing cardiothoracic surgery at the Karolinska University Hospital (Sweden).

Main Outcome Measures: Predictors of high risk of ascending aortic dilatation (defined as ascending aorta with a diameter above 40 mm) were identified through the application of machine learning algorithms and classic logistic regression models.

Exposures: Comprehensive multidimensional data, including valve morphology, clinical information, family history of cardiovascular diseases, prevalent diseases, demographic details, lifestyle factors, and medication.

Results: BAV patients, with an average age of 60.4±12.4 years, showed a higher frequency of aortic dilatation (45.3%) compared to TAV patients, who had an average age of 70.4±9.1 years (28.9% dilatation, $p<0.001$). Aneurysm prediction models for TAV patients exhibited mean AUC values above 0.8, with the absence of aortic stenosis being the primary predictor, followed by diabetes and high sensitivity C-Reactive Protein. Conversely, prediction models for BAV patients resulted in AUC values between 0.5-0.55, indicating low usefulness for predicting aortic dilatation. Classification results remained consistent across all machine learning algorithms and classic logistic regression models.

Conclusion and Recommendation: Cardiovascular risk profiles appear to be more predictive of aortopathy in TAV patients than in patients with BAV. This adds evidence to the fact that BAV- and TAV-associated aortopathy involve different pathways to aneurysm formation and highlights the need for specific aneurysm preventions in these patients. Further, our results highlight that machine learning approaches do not outperform classical prediction methods in addressing complex interactions and non-linear relations between variables.

For peer review only - http://bmjopen.bmj.com/site/about/guidelines.xhtml

STRENGTHS AND LIMITATIONS OF THIS STUDY

- **Comprehensive clinical and epidemiological data from 1034 individuals (543 BAV and 491 TAV) were analyzed, providing a robust dataset for examination.**
- **Aortic valve morphology assessment during open-heart surgery adds a significant strength, enhancing reliability compared to relying solely on echocardiography.**
- **However, the study has inherent limitations, including potential selection bias due to the exclusion of individuals with significant coronary artery disease, potentially leading to an overestimation of aneurysm prevalence.**
- **Being a surgical cohort, the study population may include individuals with worse outcomes, which could affect generalizability to counterparts of similar age in non-surgical settings.**
- **The study is limited to a single-center and mono-ethnicity, potentially impacting the generalizability of the findings to broader BAV/TAV populations.**

For peer review only

Abbreviations

AAA	Abdominal Aortic Aneurysm
AR	Aortic Regurgitation
AS	Aortic Stenosis
ASAP	Advanced Study of Aortic Pathology
BAV	Bicuscpid Aortic Valve
BMI	Body Mass Index
BSA	Body Surface Area
CVD	Cardiovascular Disease
DAVAACA	Disease of the Aortic Valve Ascending Aorta and Coronary Arteries
FFR	Fractional Flow Reserve
Hs-CRP	High Sensitivity C-Reactive Protein
LDL	Low Density Lipoprotein
LN	Left- and Non-Coronary Cusps
MCA	Multiple Correspondence Analysis
PCA	Principal ComponentAanalysis
PP	Pulse Pressure
TAA	Thoracic Aortic Aneurysm
TAV	Tricuspid Aortic Valve
TEE	Transesophageal Echocardiography
TTE	Transthoracic Echocardiography

For peer review only

Introduction

Thoracic aortic aneurysm (TAA) is a silent disease characterized by medial degradation and pathological widening of the intrathoracic aorta. Individuals with a bicuspid aortic valve (BAV) are at higher risk of developing ascending aortic aneurysm than individuals born with a normal tricuspid aortic valve (TAV).[1],[2] BAV is the most common congenital cardiac abnormality with an incidence of about 13 per 1000 live births and more than one third of all BAV patients develop aneurysm or aortic dissection later in life.[3]–[5] The underlying mechanism is not known although a turbulent flow, caused by the abnormal bicuspid valve anatomy, and/or a genetic defect have been proposed as contributing factors. The complications of BAV have led to a number of unanswered clinical questions such as: (1) what mechanisms are underlying the development of aortopathy in adults with BAV, (2) how to predict the development of aortopathy in individuals with BAV, and (3) will prediction models performance and predictors be different in patients with BAV compared to patients with TAV. Echocardiography is effective to show the degree of the sinus valsava and the annulus dilatation. However, it is more difficult to know the degree of dilation of the sinotubular junction and the ascending aorta even though they are the more frequently dilatated sections in patients with BAV[6].

Our aim was to identify clinical classifiers of prevalent aortopathy using comprehensive multidimensional clinical data. Automated machine learning methods and traditional regression models was performed on a total of 1034 subjects with either BAV or TAV.

For peer review only

METHODS

Clinical cohorts

The Advanced Study of Aortic Pathology (ASAP) study design and description have been previously published.[1],[7] Briefly, the ASAP cohort is a single-center, observational cohort study of consecutive patients with aortic valve and/or ascending aortic disease undergoing elective open-heart surgery at the Cardiothoracic Surgery Unit, Karolinska University Hospital in Stockholm, Sweden. Inclusion criteria were patients aged 18 or above with aortic valve disease (i.e., aortic stenosis; AS or aortic regurgitation; AR) and/or ascending aorta dilatation (aneurysm or ectasia) but devoid of significant coronary artery disease (stenosis >70% or Fractional Flow Reserve (FFR) <0.80) and primarily not planned for another concomitant valve surgery. The Disease of the Aortic Valve Ascending Aorta and Coronary Arteries (DAVAACA) study was set up in the continuity of the ASAP study. The ASAP/DAVAACA study started in February 2007 and include multidimensional data (blood analyses, genetic and clinical data, family history of cardiovascular diseases, prevalent diseases data, demographic characteristics, lifestyle habits data and medication). In the present study, 1180 operated patients were included (June 28, 2017). Exclusion of patients with unicuspid aortic valves, patients with missing data for cuspidity and aortic dilatation and patients with syndromic forms of TAA resulted in a final study population of 1034 subjects (e-Figure 1). Data collection and classification of AS and AR have been previously described.[1] Aortic valve function and aortic dimensions were assessed according to the standards outlined by the American Society of Echocardiography.[8]–[10]

For peer review only

168 *Echocardiography*

169 Preoperative transthoracic echocardiography (TTE) was used to determine aortic valve function. Transesophageal echocardiography (TEE) was performed on the operating table, prior to surgery, under general anaesthesia and as previously described.[11] The echocardiographic evaluation has been already pulished elsewhere.[1] TEE evaluation was used to assess valvular function, aortic root morphology and ascending aortic diameter; measurements were performed according to standards outlined by the ASE. Diameter of the ascending aorta was measured at several locations (annulus, sinus Valsalva, STJ). An ascending aorta with a diameter exceeding 40 mm was categorized as aortic dilatation in both women and men. Similarly, patients with root dilatation exceeding 40 mm were classified as having aortic dilatation. Body surface area (BSA) was calculated using Du Bois method.[12] Aortic stenosis and aortic regurgitation were defined according to standard guidelines for valve surgery.[13]

181 *Aortic valve morphology*

182 The morphology of the aortic valve was determined by inspection of the valve during surgery. Based on appearance, the valve was classified according to number of cusps and commissures. Three cusps and three commissures denote a TAV; two cusps and two commissures denote a BAV (if a remnant commissural raphe was present) or true BAV (if no raphe was present). The BAV was further classified according to cusps fusion; right- and left coronary cusps (RL), right- and non-coronary cusps (RN) or left- and non-coronary cusps (LN).[1] The Sievers classification[14], based on intraoperative analysis of the aortic valve morphology, was used to diagnose and type the bicuspid aortic valve.

191 **Statistical analysis**

For peer review only

Characteristics of the population were described using ANOVA or Chi-square tests when appropriate. Several meaningful blocks of variables were chosen (clinical data, family history of cardiovascular diseases, prevalent diseases, demographic characteristics, lifestyle habits data and medication). Principal component analysis (PCA) and Multiple correspondence analysis (MCA) were used to explain the variance-covariance structure of the variables combined. To identify variables that separated dilated versus non-dilated BAV and TAV patients, logistic regression models were used and automated machine learning algorithms were applied.

Logistic Regression

For logistic regression, a set of variables were selected to be included in the model. Since model performance can rely heavily on variable selection, different variable selection methods were tested prior to the logistic regression analysis (e-Figure 2). Firstly, variables were selected based on prior knowledge and/or biologic plausibility. Then, two automated variable selection methods were considered: (i) backward and forward elimination to optimize Akaike Information Criteria[11], (ii) and selection operator (LASSO).[12]

Machine Learning Algorithms

We used two machine learning algorithms, Random Forests and Artificial Neural Network, which are among the most widely and successfully used for clinical data.[15],[16] Each one of them represents a different algorithm "family", with different internal algorithm structures.[17] Since it was not known beforehand which kind of algorithm would perform best, algorithms with different internal structures were chosen to increase the probability of good discriminative performance. A 10-fold cross validation method to our logistic regression was also applied, allowing to split the training data into five subsets. Then, five times iteration over the five subsets were performed, such that the subset with the index same as the iteration number was

For peer review only

used as the validation test and the remaining 4 subsets were training tests. Sensitivity, specificity, and positive and negative predictive values for each predictive model were also assessed, applying a leave one out cross validation method (e-Figure 3, e-Figure 4).

Patient and Public Involvement statement

It was not possible to involve patients or the public in the design, or conduct, or reporting, or dissemination plans of our research.

RESULTS

Characteristics of BAV and TAV patients

The study population included a total of 1034 patients (52% BAV and 48% TAV) (e-Table 1, e-Figure 1). BAV patients were significantly younger (60.4 (SD 12.40) years vs. 70.4 (SD 9.09) years in TAV patients), and mainly men (73.1%) compared to TAV patients (63.3%). Furthermore, BAV patients had significantly higher body surface area (BSA) but, in contrast, a lower body mass index (BMI). AS was more common in BAV compared to TAV whereas no significant difference in the proportion of AR was found, even if the prevalence of AR was slightly higher in TAV patients. Finally, a higher prevalence of cardiovascular disease (CVD) history was observed in TAV individuals (hypertension, coronary artery disease, heart failure and stroke) and a higher percentage of family history of CVD (sibling history of myocardial infarction before 65 years of age).

For peer review only

Table 1: Baseline Characteristics of Patients with BAV or TAV (dilated vs. non-dilated ascending aorta).

	BAV patients (n=543)			TAV patients (n=491)		
	**Dilated (n=246)**	**Non-Dilated (n=297)**	**P value**	**Dilated (n=142)**	**Non-Dilated (n=349)**	**P value**
**Gender: Male**	182 (74.0%)	215 (72.4%)	0.749	89 (62.7%)	222 (63.6%)	0.927
**Age**	61.1 (11.2)	59.8 (13.3)	0.204	67.2 (10.1)	71.8 (8.28)	<0.001
**Height**	177 (9.14)	174 (9.29)	0.002	174 (9.76)	172 (9.77)	0.043
**Weight**	83.1 (15.6)	80.9 (15.8)	0.100	81.4 (16.3)	81.6 (15.7)	0.909
**BSA**	2.00 (0.21)	1.96 (0.22)	0.052	1.95 (0.22)	1.94 (0.22)	0.479
**BMI**	26.5 (4.15)	26.6 (4.36)	0.916	26.9 (4.41)	27.7 (4.90)	0.087
**Regular Smoker:**			0.176			0.537
**No**	108 (44.1%)	142 (48.8%)		61 (43.3%)	152 (43.8%)	
**Former**	117 (47.8%)	117 (40.2%)		69 (48.9%)	177 (51.0%)	
**Yes**	20 (8.16%)	32 (11.0%)		11 (7.80%)	18 (5.19%)	
**Raphe:**			0.568			
**True BAV**	24 (9.96%)	23 (7.96%)				
**Left-Non coronary**	1 (0.41%)	4 (1.38%)				
**Right-Non coronary**	43 (17.8%)	47 (16.3%)				
**Right-Left**	173 (71.8%)	215 (74.4%)				
**Systolic Blood Pressure**	135 (18.2)	136 (18.9)	0.299	144 (17.4)	142 (21.5)	0.299
**Diastolic Blood Pressure**	81.3 (12.3)	79.0 (11.6)	0.026	75.1 (13.0)	76.5 (13.3)	0.290
**Pulse Pressure (PP)**	53.3 (16.5)	57.2 (18.3)	0.009	68.7 (18.1)	65.4 (20.3)	0.076
**Leukocytes**	5.70 [4.70;6.80]	5.90 [4.97;7.00]	0.052	6.00 [5.00;7.30]	6.10 [5.10;7.40]	0.386
**hsCRP**	0.93 [0.50;2.15]	1.20 [0.59;2.80]	0.056	2.10 [0.98;4.05]	1.60 [0.71;3.80]	0.050
**Cholesterol**	4.90 [4.20;5.70]	4.80 [4.20;5.60]	0.566	4.80 [4.12;5.40]	4.60 [3.90;5.60]	0.175
**LDL**	2.90 [2.20;3.60]	2.80 [2.30;3.60]	0.813	2.90 [2.20;3.50]	2.60 [1.90;3.40]	0.064
**Aortic Stenosis:**	169 (68.7%)	235 (79.1%)	0.008	23 (16.2%)	283 (81.1%)	<0.001
**Aortic Regurgitation:**	83 (33.7%)	75 (25.3%)	0.038	93 (65.5%)	67 (19.2%)	<0.001
**Diameter Aortic Anulus**	25.5 (3.41)	24.2 (3.58)	<0.001	23.5 (3.17)	22.8 (3.03)	0.042
**Diameter Sinus Valsalva**	38.1 (5.38)	33.6 (5.11)	<0.001	40.1 (6.98)	32.8 (5.71)	<0.001
**Diameter STJ**	33.6 (5.63)	28.2 (4.51)	<0.001	35.9 (6.76)	26.6 (4.36)	<0.001
**Diameter Ascend Aorta**	46.9 (5.02)	33.0 (4.27)	<0.001	50.5 (7.90)	31.6 (4.03)	<0.001
**Sibling MI before 65 year**	13 (5.56%)	19 (6.81%)	0.688	11 (8.09%)	35 (10.8%)	0.474
**Mother MI before 65 year**	15 (6.20%)	10 (3.64%)	0.250	7 (5.26%)	25 (7.67%)	0.474
**Father MI before 65 year**	24 (10.6%)	45 (16.3%)	0.087	18 (14.1%)	45 (14.4%)	0.922
**Myocardial Infarction**	8 (3.25%)	11 (3.77%)	0.930	9 (6.43%)	33 (9.46%)	0.367
**Stroke**	15 (6.10%)	15 (5.08%)	0.746	14 (10.0%)	43 (12.3%)	0.571
**AAA**	23 (9.39%)	2 (0.68%)	<0.001	25 (18.2%)	5 (1.44%)	<0.001
**Angina Pectoris**	9 (3.66%)	26 (8.87%)	0.023	9 (6.43%)	52 (14.9%)	0.015
**Heart Failure**	15 (6.15%)	25 (8.65%)	0.354	15 (10.6%)	42 (12.2%)	0.747
**Hypertension**	115 (47.1%)	140 (47.6%)	0.979	88 (62.9%)	223 (65.0%)	0.730
**Diabetes**	15 (6.10%)	39 (13.3%)	0.009	3 (2.16%)	61 (17.5%)	<0.001
**Anti hypertensive drugs**						
**ACE inhibitor**	46 (18.7%)	57 (19.3%)	0.941	45 (31.9%)	97 (27.9%)	0.434
**Aspirin**	48 (19.5%)	84 (28.5%)	0.021	38 (27.0%)	155 (44.8%)	0.001
**Betablocker**	92 (37.4%)	125 (42.4%)	0.277	76 (53.9%)	179 (51.3%)	0.672

For peer review only

Chi square test was used for categorical variables; ANOVA for normal-distributed continuous variables; Kruskal-Wallis test for non-normal continuous variables. BSA, body surface area; BMI, body mass index; hsCRP, high sensitive C-reactive protein; LDL, low density lipoprotein; STJ, sinotubular junction; AAA, abdominal aortic aneurysm.

Aortic dilatation in BAV and TAV patients

Globally, PCA showed no separation among BAV patients with (BAV-D) or without (BAV-ND) ascendic aortic dilatation (Figure 1, Panel A), using general clinical characteristics, family history of CVD, prevalent diseases and demographics as input. By contrast, TAV patients with a dilated (TAV-D) ascending aorta clearly separated from TAV individuals with non-dilated (TAV-ND) ascending aortas (Figure 1, Panel B), indicating a more pronounced difference between these groups.

Analysis of patient descriptive data showed a pattern of significantly different associated traits between BAV-ND vs. BAV-D and TAV-ND vs. TAV-D (Table 1, e-Table 2) patients. Specifically, TAV-D patients were younger than TAV-ND patients while in the BAV group age did not differ. BAV-D patients, on the other hand, had a higher BSA and diastolic blood pressure, and a lower pulse pressure (PP) than BAV-ND patients. In both BAV and TAV patients, AR was more commonly associated with aortic dilatation, whilst AS was more prevalent in patients with non-dilated aorta. It was also noted that aortic dilatation was more localized to the tubular part of the ascending aorta in BAV patients, whereas in TAV patients the dilatation more often included the aortic root (Table 1). A positive association between dilatation and abdominal aortic aneurysm (AAA), and negative associations between dilatation and diabetes, and dilatation and angina was found in both BAV and TAV patients (Table 1).

To further analyze BAV- and TAV-associated aortopathy, a multivariable logistic regression model was performed, including age, sex, BSA, low density lipoprotein (LDL) cholesterol and high sensitivity C-Reactive Protein (hs-CRP) as covariates (Table 2). Independent variables were choosen based on previous analyses with significant differences within groups. Covariates

For peer review only

were selected based on stepwise selection and decision trees. The results showed that in BAV patients, AS, PP and diabetes were negatively associated with dilatation (adjusted models for age, sex, BSA, LDL and hsCRP were with AS: OR=0.44 (0.28-0.70), P=0.001; PP: OR=0.99 (0.98-1.0), P=0.025, and diabetes: OR=0.32 (0.16-0.62), P=0.001). Similarly, in TAV patients, dilatation was negatively associated with AS: OR=0.03 (0.02-0.06), P<0.001 and diabetes OR=0.11 (0.03-0.32), P<0.001. The magnitude of the association with AS was however 15-fold higher in TAV than in BAV patients. Aortic regurgitation was positively associated with aortic dilation in both BAV and TAV with a 5-fold higher magnitude in TAV compared to BAV patients. (Table 2). The ROC curve for the risk prediction model across the predictive methods without AS as predictor are shown in e-Figure 5.

For peer review only

Table 2. Predictors of ascending aortic dilatation in BAV and TAV patients, separately. Univariate and Multivariate logistic regression analysis.

	BAV						TAV					
	unAdjusted Model		Model 1		Model 2		unAdjusted Model		Model 1		Model 2	
	OR (95% IC)*	p	OR (95% IC)*	p	OR (95% IC)*	p	OR (95% IC)*	p	OR (95% IC)*	p	OR (95% IC)*	p
**Aortic Stenosis**	0.58 (0.39-0.85)	0.006	0.44 (0.28-0.70)	0.001	0.46 (0.24-0.88)	0.020	0.05 (0.03-0.07)	<0.001	0.03 (0.02-0.06)	<0.001	0.02 (0.01-0.05)	<0.001
**Aortic Regurgitation**	1.51 (1.04-2.19)	0.031	1.92 (1.25-2.99)	0.003	1.42 (0.76-2.66)	0.270	7.99 (5.19-12.45)	<0.001	7.47 (4.62-12.27)	<0.001	0.58 (0.23-1.36)	0.229
**Pulse Pressure**	0.99 (0.98-1.00)	0.011	0.99 (0.98-1.00)	0.025	0.98 (0.97-0.99)	0.001	1.01 (1.00-1.02)	0.091	1.00 (0.99-1.02)	0.385	0.99 (0.97-1.00)	0.096
**Diabetes**	0.42 (0.22-0.77)	0.007	0.32 (0.16-0.62)	0.001	0.36 (0.18-0.72)	0.005	0.1 (0.03-0.29)	<0.001	0.11 (0.03-0.32)	<0.001	0.26 (0.06-0.87)	0.048

* Odds ratio and 95% confidence interval limits were obtained by logistic regression
Model 1: adjusted for Sex_Male + Age + BSA + LDL + hsCRP
Model 2: adjusted for Sex_Male + Age + BSA + LDL+ hsCRP + the 3 other variables (i.e: Aortic Regurgitation, Pulse Pressure and Diabetes for Aortic Stenosis)

For peer review only

Clinical classifiers of aortic dilatation

In order to answer the question if the prevalence of aortopathy in TAV and BAV patients may be identified using clinical characteristics, automated machine learning algorithms, including Random Forests and Artificial Neural Network, and classic logistic regression models were used. This showed that the discrimination between TAV-ND and TAV-D patients was good for all models tested, with mean AUCs ranging from 0.81 to 0.88 (Figure 2, e-Table 3). The best discrimination was obtained using Random Forest (mean AUC: 0.88 (95% CI: 0.78-0.96)) and GLM (mean AUC: 0.81 (95% CI: 0.68-0.93)) (Figure 2, e-Table 3). Generalized Linear Regressions included AS, AR, PP, prevalence of diabetes, age, sex, BSA, LDL, hsCRP, BMI as variables. It was not possible to predict aortic dilatation in BAV individuals that are at high risk of developing aneurysm, as indicated by poor discrimination by all classifications models used (Figure 2, e-Table 3). This is in line with the lack of separation in the PCA.

Of note, the robustness of our classification models is supported by the results of the sensitivity analyses using imputed data (e-Table 4, e-Table 5). Supplemental e-Table 5 indicates that the main characteristics between subjects with and without missing data did not differ substantially, minimizing selection bias.

Discussion

In our analysis of 1034 Swedish subjects with bicuspid and tricuspid aortic valves with and without prelevant aortopathy but devoid of coronary artery disease and primarily not planned for another concomitant valve surgery, we report two key findings. Firstly, prediction of aortic dilatation using automated machine learning methods and traditional regression models on multidimensional clinical data was possible only among TAV individuals, and *not* in patients with BAV. This suggests that general clinical cardiovascular risk profiles play more important roles during aortic dilation in TAV patients than in patients with a BAV, and further supports

For peer review only - http://bmjopen.bmj.com/site/about/guidelines.xhtml

that aortopathy associated with BAV and TAV, respectively, are clearly distinct with different underlying etiologies. Secondly, our study shows that the classification results were consistent for all machine learning algorithms and classic logistic regression models. This suggest that machine learning approaches might not outperform classical prediction methods in addressing complex interactions and non-linear relations between variables. This unexpected finding that classical statistics outperforms machine learning merits further exploration. This may be attributed to the distinct attributes and applications of each approach. Classical statistical methods, grounded in well-established mathematical theories, offer interpretability and inferential power, excelling in scenarios where assumptions are met and relationships between variables are linear or follow specific parametric forms. Conversely, machine learning techniques, leveraging algorithms to identify patterns in complex datasets, provide flexibility and the ability to capture non-linear relationships. They are adept at handling high-dimensional data, especially when the underlying data structure is intricate or not well-defined.

The strongest predictor for ascending aortic dilatation in TAV was the absence of aortic stenosis. The other contributors to the prediction of aortic dilatation in TAV are shown in **e-Table 6.**

This is in accordance with our previous observations that surgical patients with AS and ascending aortic dilatation almost exclusively have a BAV[18]. Whether this imply that biological processes associated with the development of AS in TAV also contributes to ascending aortic stability needs to be further elucidated. AS is commonly caused by progressive calcification of the aortic valve and increase in prevalence with age. In our cohort, TAV patients with dilated ascending aortas were significantly younger than TAV patients with non-dilated ascending aortas, which may be one contributing factor to the observed association and possibly reflects the surgical nature of the cohort. However, omitting patients with AS from the analysis

For peer review only - http://bmjopen.bmj.com/site/about/guidelines.xhtml

still resulted in significant predictive values, although with worse discrimination, indicating that age alone cannot explain the association. Other strong contributors to the prediction model were diabetes and hsCRP. A negative association between diabetes and aneurysm in the abdominal aorta is well documented.[19] It has also been suggested that metformin prescription may associate with decreased risk of aortic dilatation and that the molecular mechanism involves a metformin-induced reduction in aortic inflammation.[20],[21] Similarly, an elevated hsCRP has been described in AAA patients and found to be an independent risk factor for AAA.[22],[23] Moreover, we have previously shown an inflammatory gene expression profile in dilated aortic tissue from TAV but not BAV patients.[7] In the present study, there was a borderline significance of reduced hsCRP levels in BAV patients with dilated aorta. This together implies that aortic dilatation in TAV, in these aspects, may be more similar to aneurysm of the abdominal aorta than BAV aortic dilatation.

The lack of a good model for risk prediction of BAV-associated aortopathy raises the question, which other contributing factors may be of importance for aneurysm formation and development in these individuals? Two main hypotheses have been put forth in the literature.

Firstly, an altered flow in the proximal part of the ascending aorta due to the valve malformation itself has been suggested to provoke aortic dilatation. Also, different BAV morphotypes, i.e. cusp fusion pattern, have been shown to cause flow disturbances that affect the aorta in morphotype-dependent ways.[24] In our study, flow characteristics were not included in the predition model, which possibly could have influenced the results. However, we could not see any difference in the precense of aortic dilatation between different morphotypes, i.e. true BAV, LN-, RN- or RL cusp fucion (Table 1). Of note, other factors may also influence aortic flow patterns, e.g. eccentricity of valve opening due to valve disease, or vessel stiffnes. Indeed, it

For peer review only - http://bmjopen.bmj.com/site/about/guidelines.xhtml

has been suggested that AS significantly alters aortic hemodynamic and wall shear stress, independent of aortic valve phenotype.

Secondly, the genetic contribution may override the influence of traditional risk factors to aneurysm development in patients with BAV. Although specific gene(s) and/or mutation(s) underlying BAV and BAV-associated aortopathy are still to be unravelled, several genes have been shown to be associated with both BAV formation and concomitant aortopathy in mice and humans.[25],[26],[27(p2)] Moreover, a high heratibility of BAV and/or other cardiovascular malformations have been demonstrated using segregation patterns in families, with a heritability of BAV and BAV together with other cardiovascular malformations being as high as 89% and 75%, respectively.[28] A third, most likely, possibility is that both genetic factors and abnormal hemodynamic burden play central roles in BAV-associated aortopathy, interacting with each other and therby contribute to aortic dilatation.

Further dissecting differences between patients with non-dilated and dilated ascending aortas we found that, among others, PP, AS, AR and diabetes were associated with dilatation in BAV. PP is a well known risk factor for cardiovascular disease and the clinical manifestation of increased vascular stiffness.[29] Surprisingly, PP was higher in BAV patients with a non-dilated aorta, which may seem counterintuative since AR is associated with both increased PP and aortic dilatation. However, it may be speculated that the higher PP seen in these patients may rely on structural changes due to an increased hemodynamic burden associated with a BAV. In line with this, we have previously showed that BAV patients have a qualitative collagen defect in their ascending aorta, signified by a different collagen glycation compared with TAV patients and suggestive of an altered non-enzymatic collagen crosslinking.[30] Interestingly, we have also shown that dilated ascending aorta of BAV patients display an

For peer review only

increased collagen-related stiffness compared with TAV patients.[31] Furthermore, The Strong Heart Study could also show that in patients free of prevalent coronary heart disease, aortic root dilatation was, at a given diastolic blood pressure and stroke volume, associated with lower pulse pressure.[32] In our study, Angina pectoris appears to be more prevalent in individuals with non-dilated aortas. This observation can be elucidated by the exclusion criterion of significant coronary artery stenosis, as defined in our study. Despite this exclusion criterion, angina is reported as a symptomatic manifestation. It is noteworthy that angina may be symptomatic of aortic stenosis or left ventricular hypertrophy induced by aortic stenosis. The latter condition is more frequently encountered in isolation rather than in combination with proximal aortic dilatation. Additionally, our findings indicate a higher prevalence of angina, as well as other cardiovascular conditions (such as stroke, previous myocardial infarction, etc.), and the use of medications in the group with tricuspid aortic valves (TAV), where the patients are older compared to the bicuspid aortic valve group.

Additionally, accelerated vascular aging and increased arterial stiffness has previously been described in diabetic patients, the proportion of which was higher among BAV patients with non-dilated aortas.[33] However, we and others have previously demonstrated an increased vascular inflammation in dilated aorta in TAV- but not BAV patients, suggesting that other mechanisms could be involved in the protective role of diabetes on aneurysm formation in BAV patients.[7],[34] The association between dilatation and valve disease was not as pronounced in BAV- as in TAV patients, although AS was also negatively associated with dilatation in BAV, as previously found.[35] It may be speculated that the presence of AS increases flow velocities and blood pressure in the ascending aorta, thereby stimulating vascular remodeling and strengthening of the aortic wall. Whether this hamper the process of dilatation remains to be answered. The relation between degree of stenosis and width of the ascending aorta is

For peer review only

For peer review only - http://bmjopen.bmj.com/site/about/guidelines.xhtml

complex, and a previous study found mid-ascending dilatation proportional to valve gradient when patients with small aortas were excluded.[35]

Our findings raise the issue of how to identify and implement prevention of aortopathy in BAV patients in a clinical setting. So far, clinicians have focused on aortic valve function and aortic dimensions to indicate cardiac surgery and recommend annual follow-up in asymptomatic patients to screen for associated aortopathy.[36] High importance has been given to the morphology of the valve, although in our study, dilatation in BAV did not show any significant association with valve morphology. Of note, previous studies establishing an association between BAV cusp fusion and clinical outcomes relied on small sample size based on imaging diagnostic rather than anatomic diagnosis.[37]

For peer review only

Study strengths and limitations

In this study, comprehensive clinical data, including blood sampling as well as epidemiological data was used in the analysis of in total 1034 individuals (543 BAV and 491 TAV). The morphology of aortic valves was evaluated by visual inspection during open-heart surgery, which is a major strength compared to only echocardiography in terms of reliability.

A few limitations must however be highlighted. Firstly, by design, only individuals devoid of significant coronary artery disease were included, which may introduce a selection bias and an overestimation of the prevalence of subjects with aneurysm. Secondly, as this is a population based surgical cohort, it is possible that our study population included BAV and TAV patients with worse outcomes compared to their counterparts of similar age. This should however not affect the associations between valve type and aortic dilatation. Thirdly, TAV patients with non-dilated aortas were significantly older than TAV individuals with a dilated aorta, which

For peer review only - http://bmjopen.bmj.com/site/about/guidelines.xhtml

could possibly explain the higher degree of patients with aortic stenosis in this group. Lastly our study is a single centre and mono- ethnicity study. Therefore our results may not be generalizable to other population of BAV/TAV.

To conclude, using automated machine learning algorithms and classic logistic regression models we demonstrated that in TAV patients, cardiovascular risk profiles appear to be more predictive of aortopathy than in BAV patients. The good performance of the TAV classifier also after exclusion of AS offers important implications for better targeting TAV individuals that are of a high risk of developing aneurysm. The lack of good models to develop clinical classifiers of BAV-associated aortopathy strengthen the focus of genetics and/or flow as important contributing factors to aneurysm development in these individuals.

Contributorship statement

Author Contributions: Gaye, Franco-Cereceda, Eriksson, and Björck had full access to all the data in the study and take responsibility for the integrity of the data and the accuracy of the data analysis.

Study concept and design: Gaye, Eriksson, Björck

Acquisition, analysis, or interpretation of data: Gaye, Vignac, Gådin, Ladouceur, Caidahl, Olsson, Franco-Cereceda, Eriksson, Björck

Drafting of the manuscript: Gaye

Critical revision of the manuscript for important intellectual content: Gaye, Vignac, Gådin, Ladouceur, Caidahl, Olsson, Franco-Cereceda, Eriksson, Björck

Statistical analysis: Gaye and Vignac

Study supervision: Gaye, Eriksson, Björck

Competing interests

For peer review only

The authors declare no competing interests

Funding

The work was supported by the Swedish Research Council [12660]; the Swedish Heart-Lung Foundation [20180451, 20170669]; the Stockholm County Council [20180072]; La Marató de TV3 [20151330]; and a donation by Fredrik Lundberg.

Data sharing statement

The data of this article are available upon reasonable request to the corresponding author.

Ethics Approval Statement

The study was approved by the Regional Ethics Review Board (application number 2006/784-31/1; 2012/1633-31/4), Stockholm, Sweden. Oral and written informed consent were obtained from all patients according to the declaration of Helsinki.

For peer review only

References

1. Jackson V, Petrini J, Caidahl K, Eriksson MJ, Liska J, Eriksson P, Franco-Cereceda A. Bicuspid aortic valve leaflet morphology in relation to aortic root morphology: a study of 300 patients undergoing open-heart surgery. *Eur J Cardiothorac Surg*. Published online May 2011:S1010794011004623. doi:10.1016/j.ejcts.2011.04.014
2. Messner B, Bernhard D. Bicuspid aortic valve-associated aortopathy: Where do we stand? *J Mol Cell Cardiol*. 2019;133:76-85. doi:10.1016/j.yjmcc.2019.05.023
3. Ward C. Clinical significance of the bicuspid aortic valve. *Heart*. 2000;83(1):81-85. doi:10.1136/heart.83.1.81
4. Williams DS. Bicuspid aortic valve. *J Insur Med N Y N*. 2006;38(1):72-74.
5. Losenno KL, Goodman RL, Chu MWA. Bicuspid Aortic Valve Disease and Ascending Aortic Aneurysms: Gaps in Knowledge. *Cardiol Res Pract*. 2012;2012:1-16. doi:10.1155/2012/145202
6. Verma S, Siu SC. Aortic dilatation in patients with bicuspid aortic valve. *N Engl J Med*. 2014;370(20):1920-1929. doi:10.1056/NEJMra1207059
7. Folkersen L, Wågsäter D, Paloschi V, Jackson V, Petrini J, Kurtovic S, Maleki S, Eriksson MJ, Caidahl K, Hamsten A, Michel J-B, Liska J, Gabrielsen A, Franco-Cereceda A, Eriksson P. Unraveling Divergent Gene Expression Profiles in Bicuspid and Tricuspid Aortic Valve Patients with Thoracic Aortic Dilatation: The ASAP Study. *Mol Med*. 2011;17(11-12):1365-1373. doi:10.2119/molmed.2011.00286
8. Lang RM, Bierig M, Devereux RB, Flachskampf FA, Foster E, Pellikka PA, Picard MH, Roman MJ, Seward J, Shanewise JS, Solomon SD, Spencer KT, St John Sutton M, Stewart WJ. Recommendations for Chamber Quantification: A Report from the American Society of Echocardiography's Guidelines and Standards Committee and the Chamber Quantification Writing Group, Developed in Conjunction with the European Association of Echocardiography, a Branch of the European Society of Cardiology. *J Am Soc Echocardiogr*. 2005;18(12):1440-1463. doi:10.1016/j.echo.2005.10.005
9. Zoghbi W. Recommendations for evaluation of the severity of native valvular regurgitation with two-dimensional and doppler echocardiography. *J Am Soc Echocardiogr*. 2003;16(7):777-802. doi:10.1016/S0894-7317(03)00335-3
10. Baumgartner H, Hung J, Bermejo J, Chambers JB, Evangelista A, Griffin BP, Iung B, Otto CM, Pellikka PA, Quiñones M. Echocardiographic Assessment of Valve Stenosis: EAE/ASE Recommendations for Clinical Practice. *J Am Soc Echocardiogr*. 2009;22(1):1-23. doi:10.1016/j.echo.2008.11.029
11. Walter S, Tiemeier H. Variable selection: current practice in epidemiological studies. *Eur J Epidemiol*. 2009;24(12):733-736. doi:10.1007/s10654-009-9411-2
12. Tibshirani R. The lasso method for variable selection in the Cox model. *Stat Med*. 1997;16(4):385-395. doi:10.1002/(sici)1097-0258(19970228)16:4<385::aid-sim380>3.0.co;2-3
13. Nishimura Rick A., Otto Catherine M., Bonow Robert O., Carabello Blase A., Erwin John P., Guyton Robert A., O'Gara Patrick T., Ruiz Carlos E., Skubas Nikolaos J., Sorajja Paul, Sundt Thoralf M., Thomas James D. 2014 AHA/ACC Guideline for the Management of Patients With Valvular Heart Disease: Executive Summary. *Circulation*. 2014;129(23):2440-2492. doi:10.1161/CIR.0000000000000029
14. Sievers H-H, Schmidtke C. A classification system for the bicuspid aortic valve from 304 surgical specimens. *J Thorac Cardiovasc Surg*. 2007;133(5):1226-1233. doi:10.1016/j.jtcvs.2007.01.039
15. Singal AG, Mukherjee A, Elmunzer JB, Higgins PDR, Lok AS, Zhu J, Marrero JA, Waljee AK. Machine Learning Algorithms Outperform Conventional Regression Models in Predicting Development of Hepatocellular Carcinoma: *Am J Gastroenterol*.

For peer review only

For peer review only - http://bmjopen.bmj.com/site/about/guidelines.xhtml

2013;108(11):1723-1730. doi:10.1038/ajg.2013.332

16. Lambin P, van Stiphout RGPM, Starmans MHW, Rios-Velazquez E, Nalbantov G, Aerts HJWL, Roelofs E, van Elmpt W, Boutros PC, Granone P, Valentini V, Begg AC, De Ruysscher D, Dekker A. Predicting outcomes in radiation oncology—multifactorial decision support systems. *Nat Rev Clin Oncol*. 2013;10(1):27-40. doi:10.1038/nrclinonc.2012.196

17. Fern'andez-Delgado M, Cernadas E, Barro S, Amorim D. Do we need hundreds of classifiers to solve real world classification problems? *J Mach Learn Res*. 2014;15(1).

18. Jackson V, Petrini J, Eriksson MJ, Caidahl K, Eriksson P, Franco-Cereceda A. Aortic dimensions in relation to bicuspid and tricuspid aortic valve pathology. *J Heart Valve Dis*. 2014;23(4):463-472.

19. Takagi H, Umemoto T, for the ALICE (All-Literature Investigation of Cardiovascular Evidence) Group. Negative association of diabetes with rupture of abdominal aortic aneurysm. *Diab Vasc Dis Res*. 2016;13(5):341-347. doi:10.1177/1479164116651389

20. Yu X, Jiang D, Wang J, Wang R, Chen T, Wang K, Durgahee MSA, Wei X, Cao S. Metformin prescription and aortic aneurysm: systematic review and meta-analysis. *Heart*. 2019;105(17):1351-1357. doi:10.1136/heartjnl-2018-314639

21. Li L, Mamputu J-C, Wiernsperger N, Renier G. Signaling Pathways Involved in Human Vascular Smooth Muscle Cell Proliferation and Matrix Metalloproteinase-2 Expression Induced by Leptin: Inhibitory Effect of Metformin. *Diabetes*. 2005;54(7):2227-2234. doi:10.2337/diabetes.54.7.2227

22. Vainas T, Lubbers T, Stassen FRM, Herngreen SB, van Dieijen-Visser MP, Bruggeman CA, Kitslaar PJEHM, Schurink GWH. Serum C-Reactive Protein Level Is Associated With Abdominal Aortic Aneurysm Size and May Be Produced by Aneurysmal Tissue. *Circulation*. 2003;107(8):1103-1105. doi:10.1161/01.CIR.0000059938.95404.92

23. Shangwei Z, Yingqi W, Jiang X, Zhongyin W, Juan J, Dafang C, Yonghua H, Wei G. Serum High-Sensitive C-Reactive Protein Level and CRP Genetic Polymorphisms Are Associated with Abdominal Aortic Aneurysm. *Ann Vasc Surg*. 2017;45:186-192. doi:10.1016/j.avsg.2017.05.024

24. McNally A, Madan A, Sucosky P. Morphotype-Dependent Flow Characteristics in Bicuspid Aortic Valve Ascending Aortas: A Benchtop Particle Image Velocimetry Study. *Front Physiol*. 2017;8. doi:10.3389/fphys.2017.00044

25. Yang B, Zhou W, Jiao J, Nielsen JB, Mathis MR, Heydarpour M, Lettre G, Folkersen L, Prakash S, Schurmann C, Fritsche L, Farnum GA, Lin M, Othman M, Hornsby W, Driscoll A, Levasseur A, Thomas M, Farhat L, Dubé M-P, Isselbacher EM, Franco-Cereceda A, Guo D, Bottinger EP, Deeb GM, Booher A, Kheterpal S, Chen YE, Kang HM, Kitzman J, Cordell HJ, Keavney BD, Goodship JA, Ganesh SK, Abecasis G, Eagle KA, Boyle AP, Loos RJF, Eriksson P, Tardif J-C, Brummett CM, Milewicz DM, Body SC, Willer CJ. Protein-altering and regulatory genetic variants near GATA4 implicated in bicuspid aortic valve. *Nat Commun*. 2017;8(1):15481. doi:10.1038/ncomms15481

26. Baylor-Hopkins Center for Mendelian Genomics, MIBAVA Leducq Consortium, Gould RA, Aziz H, Woods CE, Seman-Senderos MA, Sparks E, Preuss C, Wünnemann F, Bedja D, Moats CR, McClymont SA, Rose R, Sobreira N, Ling H, MacCarrick G, Kumar AA, Luyckx I, Cannaerts E, Verstraeten A, Björk HM, Lehsau A-C, Jaskula-Ranga V, Lauridsen H, Shah AA, Bennett CL, Ellinor PT, Lin H, Isselbacher EM, Lino Cardenas CL, Butcher JT, Hughes GC, Lindsay ME, Mertens L, Franco-Cereceda A, Verhagen JMA, Wessels M, Mohamed SA, Eriksson P, Mital S, Van Laer L, Loeys BL, Andelfinger G, McCallion AS, Dietz HC. ROBO4 variants predispose individuals to bicuspid aortic valve and thoracic aortic aneurysm. *Nat Genet*. 2019;51(1):42-50. doi:10.1038/s41588-018-0265-y

27. Pasipoularides A. Clinical-pathological correlations of BAV and the attendant thoracic aortopathies. Part 2: Pluridisciplinary perspective on their genetic and molecular origins. *J Mol*

For peer review only - http://bmjopen.bmj.com/site/about/guidelines.xhtml

Cell Cardiol. 2019;133:233-246. doi:10.1016/j.yjmcc.2019.05.022

28. Cripe L, Andelfinger G, Martin LJ, Shooner K, Benson DW. Bicuspid aortic valve is heritable. *J Am Coll Cardiol*. 2004;44(1):138-143. doi:10.1016/j.jacc.2004.03.050

29. Georgakis MK, Gill D, Malik R, Protogerou AD, Webb AJS, Dichgans M. Genetically Predicted Blood Pressure Across the Lifespan: Differential Effects of Mean and Pulse Pressure on Stroke Risk. *Hypertension*. 2020;76(3):953-961. doi:10.1161/HYPERTENSIONAHA.120.15136

30. Wågsäter D, Paloschi V, Hanemaaijer R, Hultenby K, Bank RA, Franco-Cereceda A, Lindeman JHN, Eriksson P. Impaired Collagen Biosynthesis and Cross-linking in Aorta of Patients With Bicuspid Aortic Valve. *J Am Heart Assoc*. 2013;2(1). doi:10.1161/JAHA.112.000034

31. Forsell C, Björck HM, Eriksson P, Franco-Cereceda A, Gasser TC. Biomechanical Properties of the Thoracic Aneurysmal Wall: Differences Between Bicuspid Aortic Valve and Tricuspid Aortic Valve Patients. *Ann Thorac Surg*. 2014;98(1):65-71. doi:10.1016/j.athoracsur.2014.04.042

32. de Simone G, Roman MJ, De Marco M, Bella JN, Izzo R, Lee ET, Devereux RB. Hemodynamic Correlates of Abnormal Aortic Root Dimension in an Adult Population: The Strong Heart Study. *J Am Heart Assoc*. 2015;4(10). doi:10.1161/JAHA.115.002309

33. Schram MT, Kostense PJ, van Dijk RAJM, Dekker JM, Nijpels G, Bouter LM, Heine RJ, Stehouwer CDA. Diabetes, pulse pressure and cardiovascular mortality: the Hoorn Study: *J Hypertens*. 2002;20(9):1743-1751. doi:10.1097/00004872-200209000-00017

34. Grewal N, Gittenberger-de Groot AC, Poelmann RE, Klautz RJM, Lindeman JHN, Goumans M-J, Palmen M, Mohamed SA, Sievers H-H, Bogers AJJC, DeRuiter MC. Ascending aorta dilation in association with bicuspid aortic valve: A maturation defect of the aortic wall. *J Thorac Cardiovasc Surg*. 2014;148(4):1583-1590. doi:10.1016/j.jtcvs.2014.01.027

35. Della Corte A, Bancone C, Quarto C, Dialetto G, Covino FE, Scardone M, Caianiello G, Cotrufo M. Predictors of ascending aortic dilatation with bicuspid aortic valve: a wide spectrum of disease expression☆. *Eur J Cardiothorac Surg*. 2007;31(3):397-405. doi:10.1016/j.ejcts.2006.12.006

36. Baumgartner H, Falk V, Bax JJ, De Bonis M, Hamm C, Holm PJ, Iung B, Lancellotti P, Lansac E, Rodriguez Muñoz D, Rosenhek R, Sjögren J, Tornos Mas P, Vahanian A, Walther T, Wendler O, Windecker S, Zamorano JL, ESC Scientific Document Group, Roffi M, Alfieri O, Agewall S, Ahlsson A, Barbato E, Bueno H, Collet J-P, Coman IM, Czerny M, Delgado V, Fitzsimons D, Folliguet T, Gaemperli O, Habib G, Harringer W, Haude M, Hindricks G, Katus HA, Knuuti J, Kolh P, Leclercq C, McDonagh TA, Piepoli MF, Pierard LA, Ponikowski P, Rosano GMC, Ruschitzka F, Shlyakhto E, Simpson IA, Sousa-Uva M, Stepinska J, Tarantini G, Tchétché D, Aboyans V, Windecker S, Aboyans V, Agewall S, Barbato E, Bueno H, Coca A, Collet J-P, Coman IM, Dean V, Delgado V, Fitzsimons D, Gaemperli O, Hindricks G, Iung B, Jüni P, Katus HA, Knuuti J, Lancellotti P, Leclercq C, McDonagh T, Piepoli MF, Ponikowski P, Richter DJ, Roffi M, Shlyakhto E, Simpson IA, Zamorano JL, Kzhdryan HK, Mascherbauer J, Samadov F, Shumavets V, Camp GV, Lončar D, Lovric D, Georgiou GM, Linhartova K, Ihlemann N, Abdelhamid M, Pern T, Turpeinen A, Srbinovska-Kostovska E, Cohen A, Bakhutashvili Z, Ince H, Vavuranakis M, et al. 2017 ESC/EACTS Guidelines for the management of valvular heart disease. *Eur Heart J*. 2017;38(36):2739-2791. doi:10.1093/eurheartj/ehx391

37. Schaefer BM, Lewin MB, Stout KK, Gill E, Prueitt A, Byers PH, Otto CM. The bicuspid aortic valve: an integrated phenotypic classification of leaflet morphology and aortic root shape. *Heart*. 2008;94(12):1634-1638. doi:10.1136/hrt.2007.132092

For peer review only

Figure 1. Principal component analysis (PCA) plot of BAV versus TAV patients (with or without aneurysm).

A, BAV patients

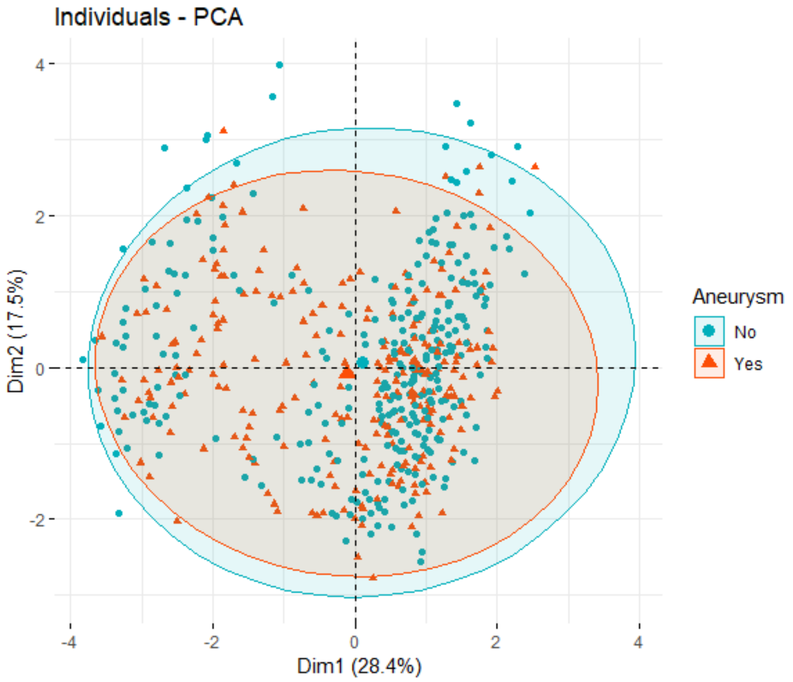

B, TAV patients

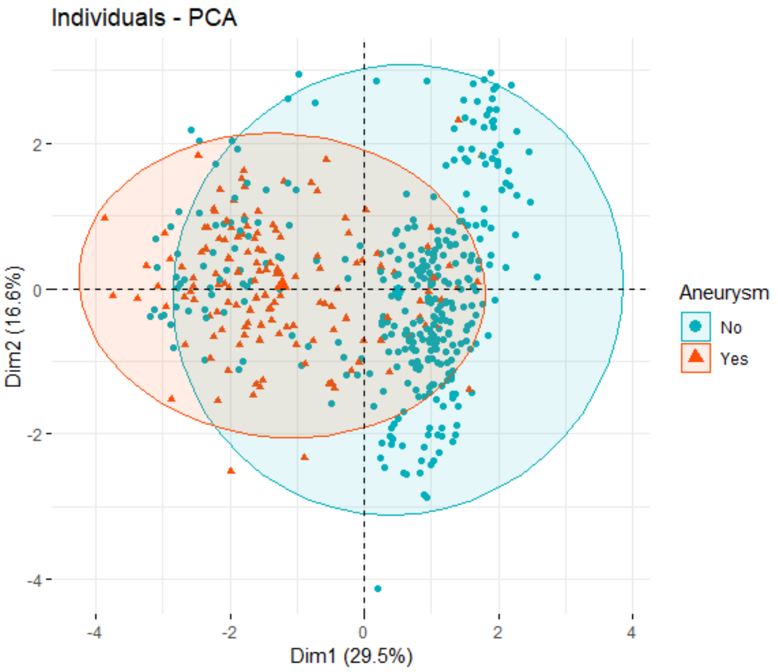

Figure 2. The ROC curve for risk prediction model across the predictive methods used.

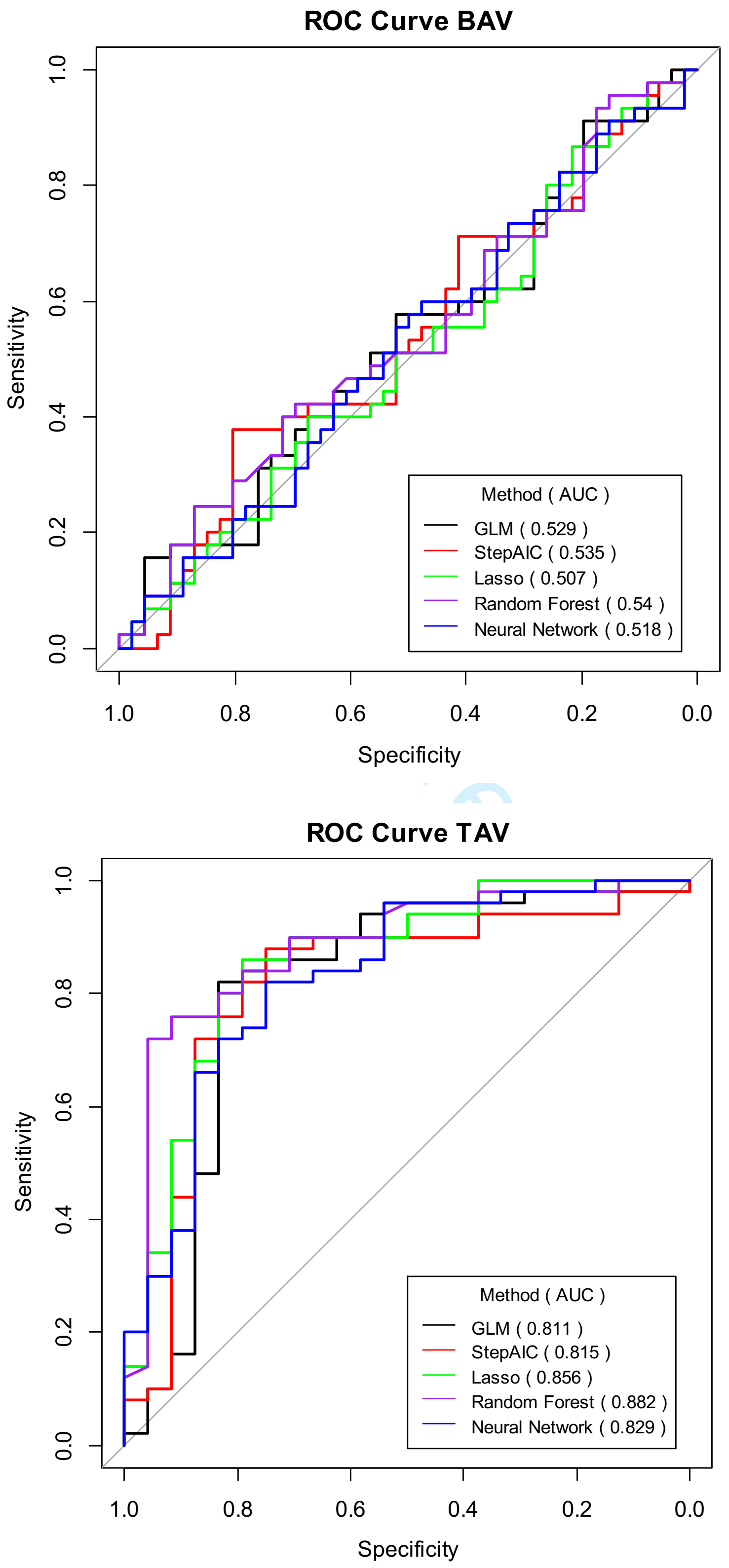

Predictive Machine Learning Models for Ascending Aortic Dilatation in Patients with Bicuspid and Tricuspid Aortic Valves Undergoing Cardiothoracic Surgery: A prospective, single-center and observational study

Supplementary Appendix Table of Contents:

Tables

e-Table 1: Characteristics of Patients with BAV versus TAV.

e-Table 2. Additional cardiovascular drugs including anti-hypertensive therapy.

e-Table 3. Performance of the algorithms.

e-Table 4: Predictors of ascending aortic dilatation in BAV and TAV patients, separately. Univariate and Multivariate logistic regression analysis: Imputed data.

e-Table 5: Baseline characteristics of included and excluded study participants.

e-Table 6: Contributors to the prediction of aortic dilatation in TAV.

Figures

e-Figure 1: Flowchart of the study

e-Figure 2: Plot of methodology used to choose variables.

e-Figure 3: The ROC curve for our risk prediction model across the predictive methods using imputed data.

e-Figure 4. Principal component analysis (PCA) plot of TAV patients with or without aneurysm stratified by AS

e-Figure 5. The ROC curve for our risk prediction model across the predictive methods without AS as predictor;

Dataset selection:

In order to gather maximum of information from the available measurement in the ASAP/DAVAACA cohort, we first created several blocks of meaningful variables: clinical data, family history of cardiovascular diseases, prevalent diseases, demographic characteristics, lifestyle habits data, blood sample data and medication. Then to select the most meaningful variables by avoiding collinearity within each bloc, we performed several analyses: (1) a hierarchical clustering, then (2) we ran Principal Component Analysis, (3) we lastly created the correlation matrix inside each cluster (and its correlation plot) (**Figure e-2**).

For peer review only

e-Table 1: Characteristics of Patients with BAV versus TAV

	BAV N=543	TAV N=491	P value
**Gender: Male**	397 (73.1%)	311 (63.3%)	0.001
**Age**	60.4 (12.4)	70.4 (9.09)	<0.001
**Height**	175 (9.30)	172 (9.80)	<0.001
**Weight**	81.9 (15.8)	81.5 (15.9)	0.697
**BSA**	1.98 (0.22)	1.94 (0.22)	0.012
**BMI**	26.6 (4.26)	27.4 (4.77)	0.002
**Regular Smoker:**			0.023
**No**	250 (46.6%)	213 (43.6%)	
**Former**	234 (43.7%)	246 (50.4%)	
**Yes**	52 (9.70%)	29 (5.94%)	
**Systolic Blood Pressure**	136 (18.6)	142 (20.4)	<0.001
**Diastolic Blood Pressure**	80.1 (11.9)	76.1 (13.2)	<0.001
**PP**	55.4 (17.6)	66.4 (19.7)	<0.001
**Leukocytes**	5.80 [4.80;6.90]	6.00 [5.03;7.40]	0.004
**hsCRP**	1.10 [0.55;2.50]	1.80 [0.79;3.80]	<0.001
**Cholesterol**	4.80 [4.20;5.60]	4.60 [3.90;5.60]	0.016
**LDL**	2.80 [2.20;3.60]	2.60 [2.00;3.50]	0.009
**Aortic Stenosis:**	404 (74.4%)	306 (62.3%)	<0.001
**Aortic Insufficiency:**	158 (29.1%)	160 (32.6%)	0.252
**Diameter of Aortic Anulus**	24.8 (3.57)	23.0 (3.08)	<0.001
**Diameter of Valsalva Sinus**	35.6 (5.68)	34.9 (6.94)	0.056
**Diameter of Sinotubular Junction**	30.5 (5.70)	29.0 (6.49)	<0.001
**Diameter of Ascending Aorta**	39.3 (8.29)	37.1 (10.1)	<0.001
**Dilatation of Ascending Aorta:**	297 (54.7%)	349 (71.1%)	<0.001
**Sibling MI history before 65 year**	32 (6.24%)	46 (10.0%)	0.041
**Mother MI history before 65 year**	25 (4.84%)	32 (6.97%)	0.199
**Father MI history before 65 year**	69 (13.7%)	63 (14.3%)	0.874
**Myocardial Infarction**	19 (3.53%)	42 (8.59%)	0.001
**Stroke**	30 (5.55%)	57 (11.7%)	0.001
**Abdominal Aorta Aneurysm**	25 (4.63%)	30 (6.19%)	0.335
**Angina Pectoris**	35 (6.49%)	61 (12.5%)	0.001
**Heart Failure**	40 (7.50%)	57 (11.7%)	0.029
**Hypertension**	255 (47.4%)	311 (64.4%)	<0.001
**Diabetes**	54 (10.0%)	64 (13.1%)	0.139
**Anti-hypertensive drugs**			
**ACE inhibitor**	103 (19.0%)	142 (29.0%)	<0.001
**Aspirin**	132 (24.4%)	193 (39.6%)	<0.001
**Betablocker**	217 (40.1%)	255 (52.0%)	<0.001

For peer review only

e-Table 2. Additional cardiovascular drugs including anti-hypertensive therapy.

	BAV patients (n=543)			TAV patients (n=491)		
	Dilated (n=246)	Non-Dilated (n=297)	P value	Dilated (n=142)	Non-Dilated (n=349)	P value
**ACE inhibitor**	46 (18.7%)	57 (19.3%)	0.941	45 (31.9%)	97 (27.9%)	0.434
**Angiotensin II blocker**	42 (17.1%)	45 (15.3%)	0.648	38 (27.0%)	78 (22.5%)	0.350
**Beta blocker**	92 (37.4%)	125 (42.4%)	0.277	76 (53.9%)	179 (51.3%)	0.672
**Calcium antagonist**	40 (16.3%)	41 (13.9%)	0.518	40 (28.4%)	87 (24.9%)	0.501
**Loop diuretics**	22 (8.94%)	35 (11.9%)	0.330	19 (13.5%)	65 (18.6%)	0.216
**Thiazide diuretics***	1 (0.81%)	1 (0.72%)	0.931	2 (2.99%)	5 (3.70%)	0.793
**Lipid lowering agent**	79 (32.1%)	107 (36.3%)	0.356	45 (32.1%)	193 (55.3%)	<0.001
**Anticoagulant**	28 (11.4%)	26 (8.81%)	0.396	12 (8.51%)	50 (14.4%)	0.107
**Insulin**	4 (1.63%)	13 (4.41%)	0.112	0 (0.00%)	17 (4.87%)	0.005
**Peroral antidiabetics***	4 (3.25%)	5 (3.60%)	0.878	2 (2.99%)	16 (11.9%)	0.069

*Results concerning these variables should be precocious due to high rate of missing value (n=464).

For peer review only

e-Table 3. Performance of the algorithms

BAV						
**Method**	**Accuracy**	**AUC**	**Sensitivity**	**Specificity**	**Positive Predictive Value**	**Negative Predictive Value**
**GLM**	0.528	0.529	0.444	0.609	0.526	0.528
**StepAIC**	0.506	0.535	0.422	0.587	0.500	0.509
**Lasso**	0.484	0.507	0.422	0.544	0.475	0.490
**Random Forest**	0.517	0.540	0.467	0.565	0.512	0.520
**Neural Network**	0.473	0.518	0.489	0.457	0.468	0.477
TAV						
**Method**	**Accuracy**	**AUC**	**Sensitivity**	**Specificity**	**Positive Predictive Value**	**Negative Predictive Value**
**GLM**	0.824	0.811	0.820	0.833	0.911	0.690
**StepAIC**	0.824	0.815	0.900	0.667	0.849	0.762
**Lasso**	0.824	0.854	0.860	0.750	0.878	0.720
**Random Forest**	0.797	0.882	0.840	0.708	0.857	0.680
**Neural Network**	0.770	0.825	0.820	0.667	0.837	0.640
BAV (Imputed data)						
**Method**	**Accuracy**	**AUC**	**Sensitivity**	**Specificity**	**Positive Predictive Value**	**Negative Predictive Value**
**GLM**	0.570	0.586	0.689	0.426	0.593	0.531
**StepAIC**	0.563	0.526	0.676	0.426	0.588	0.520
**Lasso**	0.578	0.593	0.676	0.459	0.602	0.539
**Random Forest**	0.556	0.556	0.662	0.426	0.583	0.548
**Neural Network**	0.541	0.540	0.581	0.492	0.581	0.492
TAV (Imputed data)						
**Method**	**Accuracy**	**AUC**	**Sensitivity**	**Specificity**	**Positive Predictive Value**	**Negative Predictive Value**
**GLM**	0.820	0.831	0.816	0.829	0.922	0.644
**StepAIC**	0.803	0.838	0.816	0.771	0.899	0.628
**Lasso**	0.812	0.842	0.793	0.857	0.932	0.625
**Random Forest**	0.820	0.857	0.828	0.800	0.911	0.651
**Neural Network**	0.812	0.799	0.862	0.686	0.872	0.667

For peer review only

e-Table 4. Predictors of aortopathy in BAV and TAV patients separately, Univariate and Multivariate logistic regression analysis: Imputed data

	**BAV imputed (N= 543)**						**TAV imputed (n= 491)**					
	unAdjusted Model		***Model 1***		***Model 2***		***unAdjusted Model***		***Model 1***		***Model 2***	
	OR (95% IC)*	***p***	***OR (95% IC)****	***p***	***OR (95% IC)****	***p***	***OR (95% IC)****	***p***	***OR (95% IC)****	***p***	***OR (95% IC)****	***p***
**Aortic Stenosis**	0.58 (0.39-0.85)	0.006	0.43 (0.27-0.67)	0.002	0.42 (0.22-0.79)	0.007	0.05 (0.03-0.07)	<0.001	0.04 (0.02-0.07)	<0.001	0.02 (0.01-0.05)	<0.001
**Aortic Regurgitation**	1.51 (1.04-2.19)	0.031	1.89 (1.24-2.88)	0.003	1.35 (0.75-2.44)	0.316	7.99 (5.19-12.45)	<0.001	7.27 (4.56-11.77)	<0.001	0.51 (0.20-1.18)	0.133
**Pulse Pressure**	0.99 (0.98-1.00)	0.009	0.99 (0.98-1.00)	0.013	0.98 (0.97-0.99)	<0.001	1.01 (1.00-1.02)	0.088	1.01 (1.00-1.02)	0.163	0.99 (0.98-1.00)	0.132
**Diabetes**	0.42 (0.22-0.76)	0.006	0.34 (0.18-0.65)	0.001	0.39 (0.19-0.74)	0.005	0.10 (0.02-028)	<0.001	0.10 (0.02-0.29)	<0.001	0.21 (0.05-0.71)	0.022

* Odds ratio and 95% confidence interval limits were obtained by logistic regression and were adjusted for age, sex, body surface area (BSA), low-density Lipoprotein (LDL), high-sensitivity C-reactive protein (hsCRP) and Diabetes

For peer review only

e-Table 5. Baseline characteristics of included and excluded study participants

	[ALL] N=1180	Data N=1041	NA N=139	p.overall	N
**Gender: Male**	807 (68.4%)	708 (68.5%)	99 (67.8%)	0.947	1180
**Age**	64.9 (12.4)	65.2 (12.0)	62.9 (14.5)	0.077	1180
**Height**	174 (9.60)	174 (9.65)	175 (9.15)	0.263	1171
**Weight**	81.8 (15.6)	81.7 (15.8)	82.6 (14.4)	0.514	1168
**BSA**	1.96 (0.21)	1.96 (0.22)	1.98 (0.19)	0.305	1141
**BMI**	27.0 (4.56)	27.0 (4.53)	27.1 (4.80)	0.746	1167
**Regular Smoker:**				0.549	1168
**No**	535 (45.8%)	463 (45.2%)	72 (50.0%)		
**Former**	541 (46.3%)	480 (46.9%)	61 (42.4%)		
**Yes**	92 (7.88%)	81 (7.91%)	11 (7.64%)		
**Cusps:**				0.951	1140
**BAV**	599 (52.5%)	543 (52.5%)	56 (52.8%)		
**TAV**	541 (47.5%)	491 (47.5%)	50 (47.2%)		
**Raphe:**				.	1158
**True BAV**	50 (4.32%)	47 (4.60%)	3 (2.19%)		
**Left-Non coronary**	6 (0.52%)	5 (0.49%)	1 (0.73%)		
**Right-Non coronary**	99 (8.55%)	90 (8.81%)	9 (6.57%)		
**Right-Left**	432 (37.3%)	388 (38.0%)	44 (32.1%)		
**Systolic Blood Pressure**	138 (19.7)	139 (19.8)	136 (18.8)	0.053	1172
**Diastolic Blood Pressure**	78.3 (12.7)	78.2 (12.7)	79.3 (12.7)	0.305	1171
**PP**	60.1 (19.2)	60.6 (19.4)	56.2 (17.2)	0.005	1171
**Leukocytes**	5.90 [4.90;7.10]	5.90 [4.90;7.10]	5.90 [4.90;6.90]	0.665	1168
**hsCRP**	1.40 [0.64;3.20]	1.40 [0.63;3.10]	1.60 [0.72;3.52]	0.153	1159
**Cholesterol**	4.80 [4.00;5.68]	4.80 [4.00;5.60]	5.10 [4.30;6.10]	0.004	1162
**LDL**	2.80 [2.20;3.60]	2.80 [2.10;3.50]	3.20 [2.30;3.90]	0.002	1146
**Aortic Stenosis:**	813 (69.1%)	710 (68.7%)	103 (72.0%)	0.472	1177
**Aortic Insufficiency:**	361 (30.7%)	318 (30.8%)	43 (30.1%)	0.944	1177
**Diameter of Aortic Anulus**	23.9 (3.40)	24.0 (3.46)	23.5 (2.87)	0.148	1154
**Diameter of Valsalva Sinus**	35.4 (6.27)	35.3 (6.32)	36.2 (5.83)	0.095	1157
**Diameter of Sinotubular Junction**	29.9 (6.05)	29.8 (6.13)	30.7 (5.34)	0.083	1112
**Diameter of Ascending Aorta**	38.3 (9.24)	38.2 (9.28)	38.8 (8.09)	0.686	1069
**Dilatation of Ascending Aorta:**	402 (37.6%)	388 (37.5%)	14 (40.0%)	0.904	1069
**Sibling MI history before 65-y**	86 (7.76%)	78 (8.02%)	8 (5.93%)	0.497	1108
**Mother MI history before 65-y**	63 (5.65%)	57 (5.84%)	6 (4.29%)	0.583	1116
**Father MI history before 65-y**	151 (14.0%)	132 (14.0%)	19 (14.2%)	0.959	1076
**Myocardial Infarction**	66 (5.64%)	61 (5.94%)	5 (3.47%)	0.313	1171
**Stroke**	99 (8.43%)	87 (8.45%)	12 (8.33%)	0.964	1174
**Abdominal Aorta Aneurysm**	58 (4.96%)	55 (5.37%)	3 (2.07%)	0.132	1170
**Angina Pectoris**	103 (8.80%)	96 (9.35%)	7 (4.86%)	0.105	1171
**Heart Failure**	112 (9.64%)	97 (9.52%)	15 (10.5%)	0.828	1162
**Hypertension**	635 (54.5%)	566 (55.4%)	69 (47.6%)	0.092	1166
**Diabetes**	136 (11.6%)	118 (11.5%)	18 (12.4%)	0.852	1172
**Anti-hypertensive drugs**					
**ACE inhibitor:**	277 (23.6%)	245 (23.8%)	32 (22.4%)	0.790	1173
**Aspirin:**	352 (30.1%)	325 (31.6%)	27 (18.9%)	0.003	1171
**Betablocker:**	530 (45.1%)	472 (45.8%)	58 (40.6%)	0.277	1174

For peer review only

e-Table 6. Contributors to the predictive classifiers in TAV

GLM		StepAIC (top 10)		Lasso (top 10)		RF (top 10)		Neural network (top 10)	
**AS**	100.000	**AS**	100.000	**AS**	100.000	**AS**	100.000	**AS**	100.00
**age**	27.762	**AI**	70.043	**Diabetes**	39.6265	**AI**	23.581	**age**	62.81
**Diabetes**	27.003	**age**	41.852	**Renal Failure**	26.2556	**Creatinine**	21.571	**Diabetes**	47.28
**AI**	21.226	**LDL**	31.027	**Father Stroke o r Mi or TIA65**	16.4437	**age**	21.181	**Beta blocker**	41.58
**BSA**	17.970	**Hemoglobin**	27.641	**MI**	15.3655	**PP**	16.418	**Creatinine**	38.18
**Pulse BP**	16.595	**ASA**	26.802	**ALAT**	14.7816	**Leukocytes**	14.558	**FatherMI65**	35.95
**LDL**	8.753	**Cholesterol**	26.657	**Sibling AP65**	14.7506	**hsCRP**	13.549	**Diastolic BP**	34.47
**Gender**	7.564	**Diabetes**	26.541	**HF**	12.8772	**Systolic BP**	13.540	**Deep VTPEM**	33.27
**hsCRP**	0.000	**hsCRP**	22.648	**Father MI65**	12.4264	**Height**	13.199	**Father Stroke or Mi or TIA65**	33.16

For peer review only

e-Figure 1: Flowchart of the study

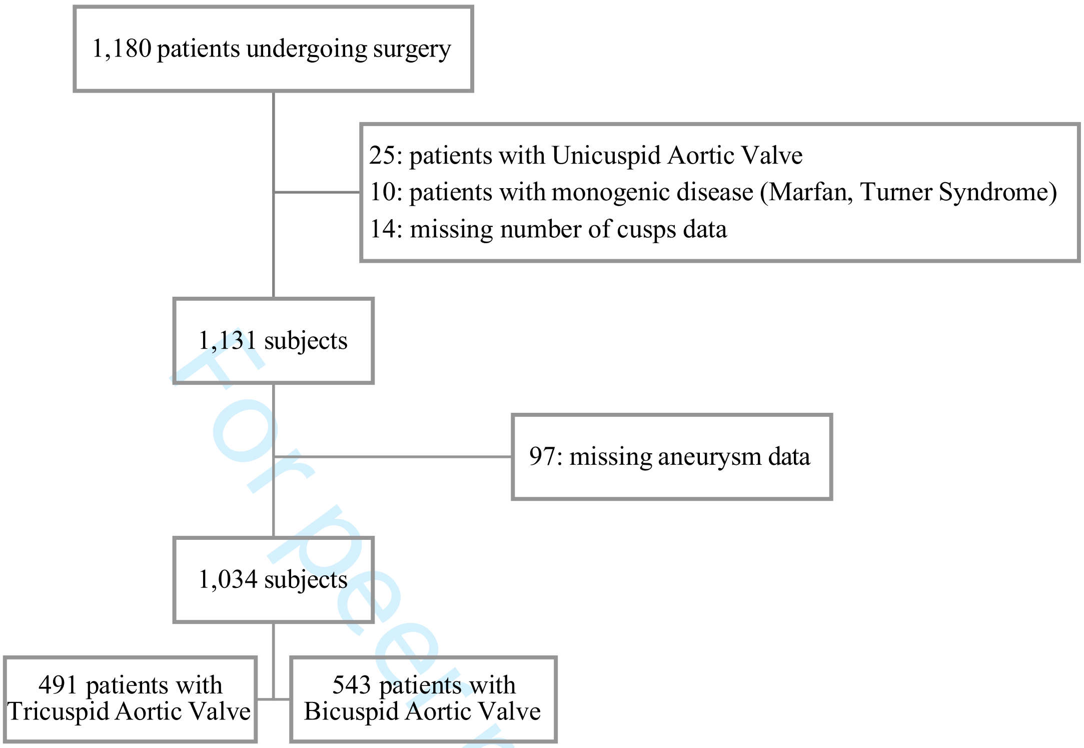

For peer review only

e-Figure 2. Plot of methodology used to choose variables

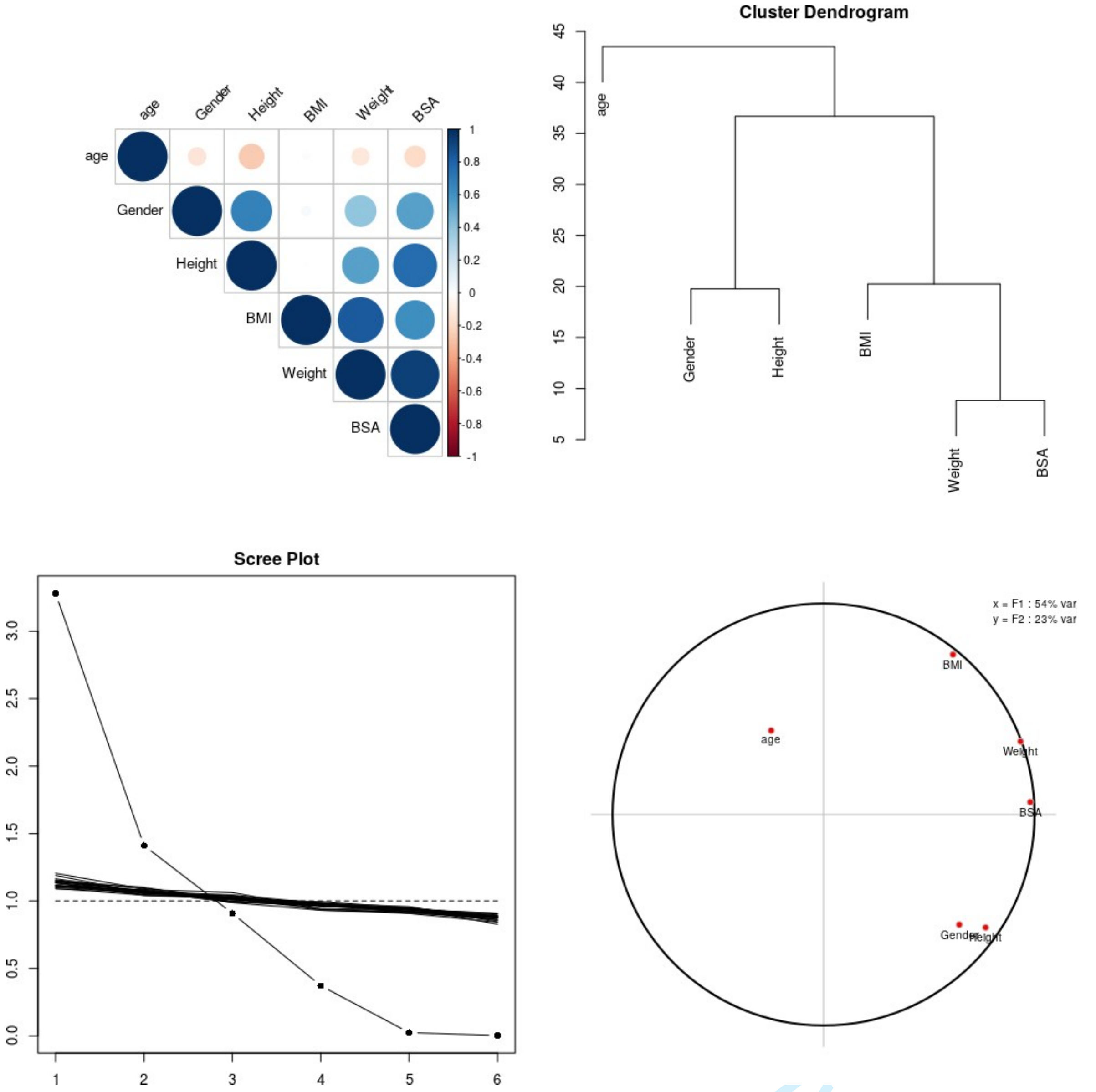

e-Figure 3. The ROC curve for our risk prediction model across the predictive methods using imputed data

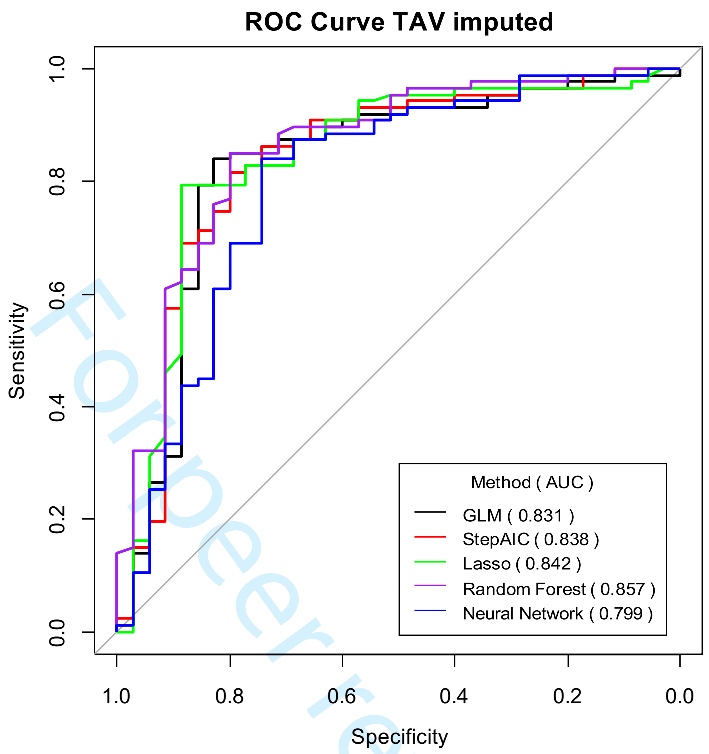

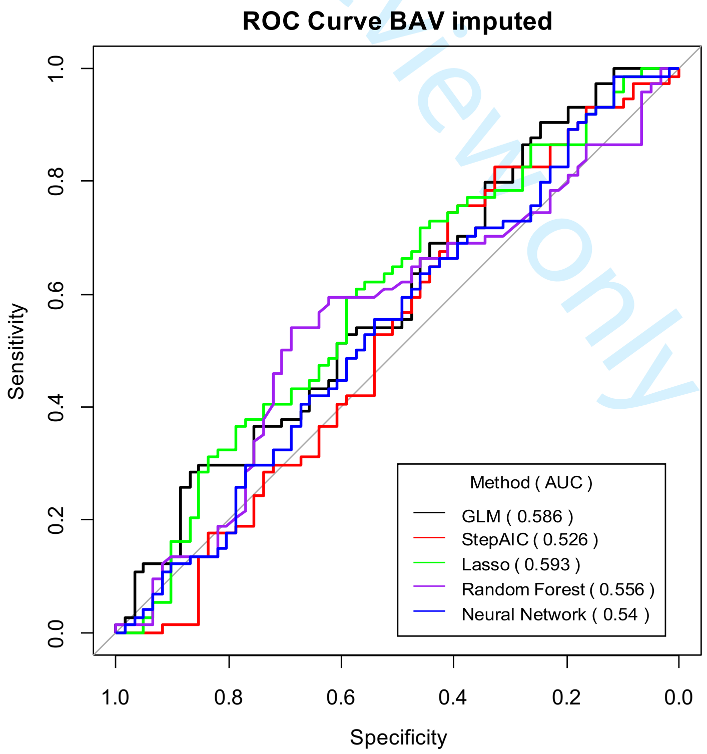

For peer review only

e-Figure 4. Principal component analysis (PCA) plot of TAV patients with or without aneurysm stratified by AS

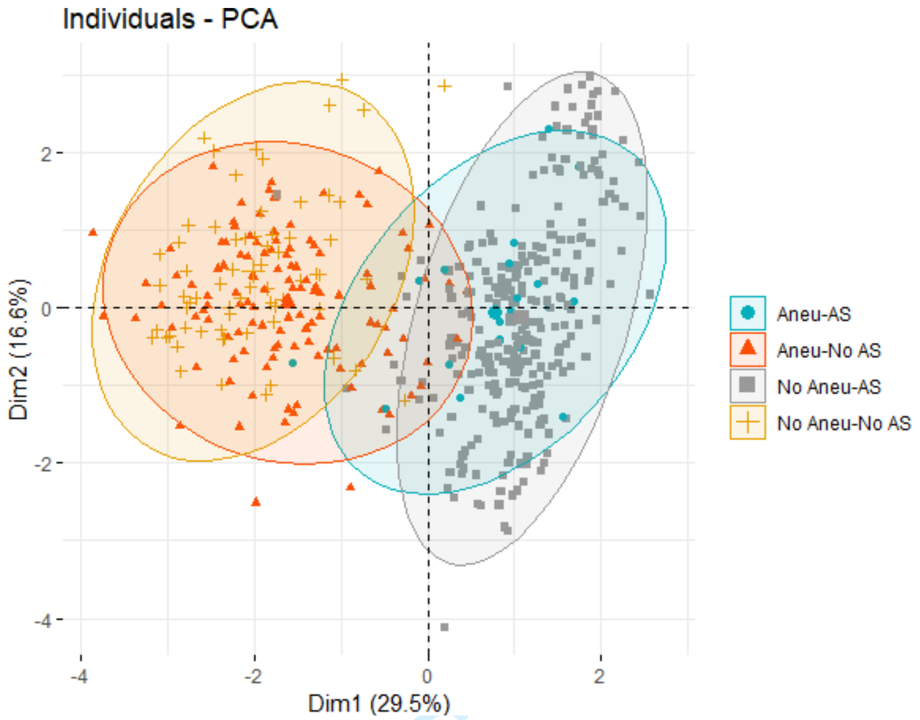

For peer review only

e-Figure 5. The ROC curve for our risk prediction model across the predictive methods without AS as predictor

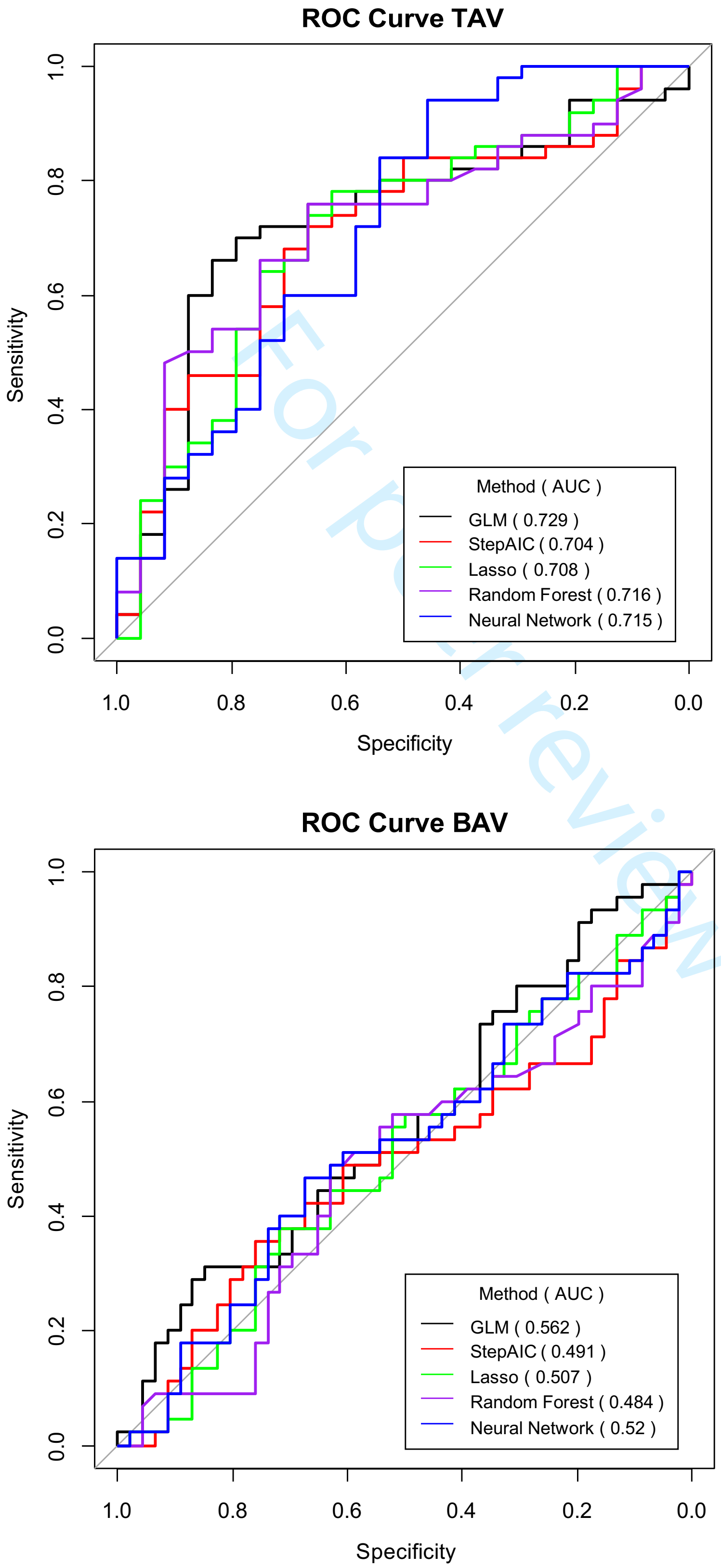

For peer review only

Measurements and definitions

Hypertension persons are defined as persons whose blood pressure measurement at the current year's health check-up is ≥ 140 mmHg systolic (1 mmHg = 0.1333 kPa) and/or 90 mmHg diastolic, or who have been diagnosed as hypertensive (1).

Diabetic patients are defined as patients whose fasting blood glucose is currently controlled at < 7.0 mmol/L or whose glycated haemoglobin is < 7% (2).

Dyslipidemia: those with triglycerides ≥ 2.26 mmol/L and/or total cholesterol ≥ 6.22 mmol/L and/or HDL cholesterol < 1.04 mmol/L and/or LDL cholesterol ≥ 4.14 mmol/L (3).

1. *Mancia G, Kreutz R, Brunström M, Burnier M, Grassi G, Januszewicz A, Muiesan ML, Tsioufis K, Agabiti-Rosei E, Algharably EAE, Azizi M, Benetos A, Borghi C, Hitij JB, Cifkova R, Coca A, Cornelissen V, Cruickshank JK, Cunha PG, Danser AHJ, Pinho RM, Delles C, Dominiczak AF, Dorobantu M, Doumas M, Fernández-Alfonso MS, Halimi JM, Járai Z, Jelaković B, Jordan J, Kuznetsova T, Laurent S, Lovic D, Lurbe E, Mahfoud F, Manolis A, Miglinas M, Narkiewicz K, Niiranen T, Palatini P, Parati G, Pathak A, Persu A, Polonia J, Redon J, Sarafidis P, Schmieder R, Spronck B, Stabouli S, Stergiou G, Taddei S, Thomopoulos C, Tomaszewski M, Van de Borne P, Wanner C, Weber T, Williams B, Zhang ZY, Kjeldsen SE. 2023 ESH Guidelines for the management of arterial hypertension The Task Force for the management of arterial hypertension of the European Society of Hypertension: Endorsed by the International Society of Hypertension (ISH) and the European Renal Association (ERA). J Hypertens. 2023 Dec 1;41(12):1874-2071. doi: 10.1097/HJH.0000000000003480. Epub 2023 Sep 26. PMID: 37345492.*
2. *Marx N, Federici M, Schütt K, Müller-Wieland D, Ajjan RA, Antunes MJ, Christodorescu RM, Crawford C, Di Angelantonio E, Eliasson B, Espinola-Klein C, Fauchier L, Halle M, Herrington WG, Kautzky-Willer A, Lambrinou E, Lesiak M, Lettino M, McGuire DK, Mullens W, Rocca B, Sattar N; ESC Scientific Document Group. 2023 ESC Guidelines for the management of cardiovascular disease in patients with diabetes. Eur Heart J. 2023 Oct 14;44(39):4043-4140. doi: 10.1093/eurheartj/ehad192. Erratum in: Eur Heart J. 2023 Dec 21;44(48):5060. PMID: 37622663.*
3. *Mach F, Baigent C, Catapano AL, Koskinas KC, Casula M, Badimon L, Chapman MJ, De Backer GG, Delgado V, Ference BA, Graham IM, Halliday A, Landmesser U, Mihaylova B, Pedersen TR, Riccardi G, Richter DJ, Sabatine MS, Taskinen MR, Tokgozoglu L, Wiklund O; ESC Scientific Document Group. 2019 ESC/EAS Guidelines for the management of dyslipidaemias: lipid modification to reduce cardiovascular risk. Eur Heart J. 2020 Jan 1;41(1):111-188. doi: 10.1093/eurheartj/ehz455. Erratum in: Eur Heart J. 2020 Nov 21;41(44):4255. PMID: 31504418.*

For peer review only

STROBE Statement—Checklist of items that should be included in reports of ***cohort studies***

	Item No	Recommendation	Page No
**Title and abstract**	1	(*a*) Indicate the study's design with a commonly used term in the title or the abstract	1
		(*b*) Provide in the abstract an informative and balanced summary of what was done and what was found	2
**Introduction**			
Background/rationale	2	Explain the scientific background and rationale for the investigation being reported	4
Objectives	3	State specific objectives, including any prespecified hypotheses	4
**Methods**			
Study design	4	Present key elements of study design early in the paper	5
Setting	5	Describe the setting, locations, and relevant dates, including periods of recruitment, exposure, follow-up, and data collection	5
Participants	6	(*a*) Give the eligibility criteria, and the sources and methods of selection of participants. Describe methods of follow-up	5
		(*b*) For matched studies, give matching criteria and number of exposed and unexposed	
Variables	7	Clearly define all outcomes, exposures, predictors, potential confounders, and effect modifiers. Give diagnostic criteria, if applicable	5, 6, 7,
Data sources/ measurement	8*	For each variable of interest, give sources of data and details of methods of assessment (measurement). Describe comparability of assessment methods if there is more than one group	5, 6, 7
Bias	9	Describe any efforts to address potential sources of bias	5, 6
Study size	10	Explain how the study size was arrived at	e-fig 1
Quantitative variables	11	Explain how quantitative variables were handled in the analyses. If applicable, describe which groupings were chosen and why	5, 6, 7
Statistical methods	12	(*a*) Describe all statistical methods, including those used to control for confounding	5, 6, 7
		(*b*) Describe any methods used to examine subgroups and interactions	5, 6, 7
		(*c*) Explain how missing data were addressed	
		(*d*) If applicable, explain how loss to follow-up was addressed	
		(*e*) Describe any sensitivity analyses	7, 8, 13
**Results**			
Participants	13*	(a) Report numbers of individuals at each stage of study—eg numbers potentially eligible, examined for eligibility, confirmed eligible, included in the study, completing follow-up, and analysed	8, 9
		(b) Give reasons for non-participation at each stage	
		(c) Consider use of a flow diagram	
Descriptive data	14*	(a) Give characteristics of study participants (eg demographic, clinical, social) and information on exposures and potential confounders	8, 9, 10
		(b) Indicate number of participants with missing data for each variable of interest	9
		(c) Summarise follow-up time (eg, average and total amount)	
Outcome data	15*	Report numbers of outcome events or summary measures over time	

For peer review only - http://bmjopen.bmj.com/site/about/guidelines.xhtml

Main results	16	(*a*) Give unadjusted estimates and, if applicable, confounder-adjusted estimates and their precision (eg, 95% confidence interval). Make clear which confounders were adjusted for and why they were included<br>(*b*) Report category boundaries when continuous variables were categorized<br>(*c*) If relevant, consider translating estimates of relative risk into absolute risk for a meaningful time period	11, 12, 13
Other analyses	17	Report other analyses done—eg analyses of subgroups and interactions, and sensitivity analyses	
**Discussion**			
Key results	18	Summarise key results with reference to study objectives	13, 14
Limitations	19	Discuss limitations of the study, taking into account sources of potential bias or imprecision. Discuss both direction and magnitude of any potential bias	14, 15, 19
Interpretation	20	Give a cautious overall interpretation of results considering objectives, limitations, multiplicity of analyses, results from similar studies, and other relevant evidence	15, 16, 17
Generalisability	21	Discuss the generalisability (external validity) of the study results	17, 18
**Other information**			
Funding	22	Give the source of funding and the role of the funders for the present study and, if applicable, for the original study on which the present article is based	19

*Give information separately for exposed and unexposed groups.

Note: An Explanation and Elaboration article discusses each checklist item and gives methodological background and published examples of transparent reporting. The STROBE checklist is best used in conjunction with this article (freely available on the Web sites of PLoS Medicine at http://www.plosmedicine.org/, Annals of Internal Medicine at http://www.annals.org/, and Epidemiology at http://www.epidem.com/). Information on the STROBE Initiative is available at http://www.strobe-statement.org.

For peer review only
